# IL-1β induced down-regulation of miR-146a-5p promoted pyroptosis and apoptosis of corneal epithelial cell in dry eye disease through targeting STAT3

**DOI:** 10.1186/s12886-024-03396-8

**Published:** 2024-03-29

**Authors:** Xuejiao Li, Hua Peng, Jianshu Kang, Xiaomei Sun, Jian Liu

**Affiliations:** 1 Department of Ophthalmology, Affiliated Hospital of Yunnan University, 650500 Kunming, Yunnan China; 2 Department of Ophthalmology, China Academy of C.M.S. Eye Hospital, NO. 33 Lugu Road, Shijingshan District, 100040 Beijing, China

**Keywords:** Dry eye disease, IL-1β, miR-146a-5p, STAT3, Pyroptosis

## Abstract

**Aim:**

To elaborate the underlying mechanisms by which IL-1β promote progression of Dry eye disease(DED) through effect on pyroptosis and apoptosis of corneal epithelial cells(CECs).

**Methods:**

400 mOsM solutions were used to establish the DED model (hCECs- DED). RT-qPCR was performed to measure IL-1β mRNA and miR-146a-5p in CECs. Western blotting was performed to measure STAT3, GSDMD, NLRP3, and Caspase-1 levels. Cell counting kit-8 assay was adopted to check cell viability. Apoptosis was detected by flow cytometry. ELISAs were performed to determine IL-18, IL-33 and LDH. The luciferase test detects targeting relationships.

**Results:**

After treatment with 400 mOsM solution, cell viability decreased and apoptosis increased. Compared with hCECs, IL-1β was increased and miR-146a-5p was decreased in hCECs-DED. At the same time, GSDMD, NLRP3, Caspase-1, IL-18, IL-33 and LDH were significantly higher in hCECs-DED than in hCECs, while IL-1β silencing reversed this effect. In addition, IL-1β negatively regulated miR-146a-5p. MiR-146a-5p mimics eliminated the inhibition of hCECs-DED pyroptosis and apoptosis caused by IL-1β silencing. At the same time, miR-146a-5p reduced STAT3 levels in hCECs.

**Conclusion:**

Highly expressed IL-1β promoted pyroptosis and apoptosis of hCECs- DED through downregulated miR-146a-5p and inhibited STAT3.

**Supplementary Information:**

The online version contains supplementary material available at 10.1186/s12886-024-03396-8.

## Introduction

Dry eye disease (DED) is very common and widespread around the world, with a prevalence of up to 50% [1, 2]. Clinical manifestations of DED are persistent irritation, and can be subdivided into aqueous-deficient DED and hyperevaporative DED. If untreated, it can lead to inflammatory damage to the cornea or conjunctiva, which can have an adverse impact on patients’ quality of life and work productivity. Due to damage to corneal epithelial cells (CECs), it is one of the most important events in the progression of DED [3], and there was a study found that pyroptosis of CECs plays a pivotal role in DED [4]. Therefore, the aim of this paper is to investigate the mechanism of pyroptosis of CECs in dry eyes.

DED is associated with inflammation of the ocular surface [5]. Studies have found that levels of inflammatory cytokines are mostly elevated in patients with DED [6]. As one of the most important pro-inflammatory cytokines, the role of interleukin-1 beta (IL-1β) in DED has attracted much attention of researchers [7, 8]. There was a report indicated that inhibiting IL-1β relieves DED by suppresses cular surface injury [9]. While the mechanism of IL-1β promote the progress of DED is not clear.

miRNAs are small noncoding RNAs that regulate various physiological events or diseases [10]. First discovered in the 1990s, the function of miRNAs in DED has been explored [11]. Among them, miR-146a-5p (previously miR-146a) kindled our interest. It is in the second exon of the human LOC285628 gene. It suppresses the immune system and inflammation. It has also been shown to be associated with the pathogenesis of DED [12]. Nowadays, increasing evidences indicated that the expression of some miRNAs can be regulated by IL-1β [13]. Furthermore, miR-146a-5p is also an IL-1β-responsive miRNA [14]. Here, we studied the function of IL-1β and miR-146a-5p in DED, and the underlying mechanism by which they affect DED.

In order to provides a potential therapeutic target for DED, in this study, the mechanism that IL-1β regulate pyroptosis and apoptosis of CECs through miR-146a-5p/STAT3 axis were explored.

## Methods

### Cell treatment

Human corneal epithelial cell line (hCECs, #6510, Sciencell, USA) from ATCC was maintained in DMEM with 10% FBS, EGF, insulin, and antibiotics at 37 °C. Hyperosmotic treatment was used to establish hyperosmotic DED model in vitro. In brief, NaCl was used to prepare 400 mOsM solution, then hCECs were treated for 24 h as hCECs-DED). At the same time, 312 mOsM NaCl was used as the control group (hCECs group).

### Transfection

Empty vector (Vector), IL-1β silencing vector (si-IL-1β), miR-146a-5p inhibitor, miR-146a-5p mimics and STAT3 overexpression vector (OE-STAT3) were obtained from Beyotime and transfected into cell by using lipo2000. In this study, according to the experimental group needs, miR-146a-5p was overexpressed in hCECs-DED 1 h before 24 h IL-1β treatment.

### RT-qPCR

RNA was collected using RNeasy Mini Kit (QIAGEN). For mRNA quantification, the TruScript Reverse Transcriptase (#NGB-54440, Norgen Biotek, Canada) was used to reverse the total RNA to cDNA. Subsequently, RT-qPCR were performed by using real-time PCR 7500 (#4351104, Thermofisher, Singapore). For miRNA quantification, the miRNA first strand Cdna synthesis kit (PC4801, Aidlab Biotechnologies, China) was used to reverse the total RNA to cDNA. Real-time quantitative PCR was performed using the miRNA-Real Time PCR Assay kit (PC4901, Aidlab Biotechnologies, China). The primers (5’-3’) were: IL-1β F: ACTGAGGACGTTCACCGTCTA, R GTGGGTGAATCTTAACTGGTT; miR-146a-5p F: CTGAGAACTGAATTCCATGGGTT, R GTGCAGGGTCCGAGGT; GAPDH F: TCCACGGTAAGCGGCATATGCTCT, R GCGCATTACCACGAACTCCATTCA; U6 F: CTTGCATCCGCATCAGA, R AATGCATCATGAAGTTCCGA. The internal controls were GADPH and U6. Gene fold change was calculated by 2^−ΔΔCt^ [15].

### Western blotting

Proteins were isolated with a commercial kit (Millipore), and quantified with a BCA kit (FuShen, Shanghai). Proteins in equal volumes were separated using 10% SDS-PAGE (Ybscience) and electrophoretically blotted to PVDF, #YB101123-1, Ybscience) membrane. The blots were performed with 1st antibodies listed as follow, anti-GSDMD (1:2000, #XGK103775, Xige, Shanghai, China), anti-NLRP3 (1:2000, #XGK99167, Xige), anti-Caspase-1 (1:2000, #XGKflu99500, Xige) and anti-GAPDH (1:3000, #XGK104170, Xige). After treated with secondary antibody (1:1000, #XG-X10933, Xige), respective target proteins of the antibodies were developed by using electrochemiluminescence (ECL, #abs920, Absin, Shanghai, China). The amount of target proteins calculated by gray scanning [16].

### Cell counting kit-8 assay

Cell counting kit-8 assay (CCK-8, Takara Bio, China) was used to measure cell growth [17]. Cells were cultured in 96-well plates, CCK-8 (10µL) was provided to each well and kept for 2 h incubation, and OD450 was read with a microplate reader.

### ELISA

Human interleukin 18 (IL-18) ELISA Kit (#CK-E10092, Sino best, Wuhan, China), human interleukin 33 (IL-33) ELISA Kit (#EH0198, Fine test, Wuhan, China) and human lactate dehydrogenase (LDH) ELISA Kit (#CK-E10891, Sino best) were used to measure the level of IL-18, IL-33 and LDH in each group.

### Double luciferase reporter genes assay

Briefly, wild type/ mutant 3’UTR of STAT3 (STAT3-WT, STAT3-MUT) were ligated into vectors, and then co-transfected each kind of them with miR-146a-5p mimics into hCECs using lipo2000. Luciferase was quantified by Nano-Glo® Dual-Luciferase® System (Promega) 48 h later [18].

### Apoptosis analysis

Apoptosis was detected using flow cytometry. After treatment of cells according to different groupings, cells were stained with the Annexin V/FITC Kit (BD Biosciences, USA) according to the kit instructions.

### Statistical analysis

Experiments in this study were independently performed 3 times, and data was shown as means ± SD. Data was analyzed by SPSS 22.0, and figure was mapped by using GraphPad Prism 7. Differences were analyzed by Student’s t test or one-way ANOVA. *P* < 0.05 was defined statistically significant.

## Results

### DED causes pyroptosis of hCECs

**Fig. 1:**
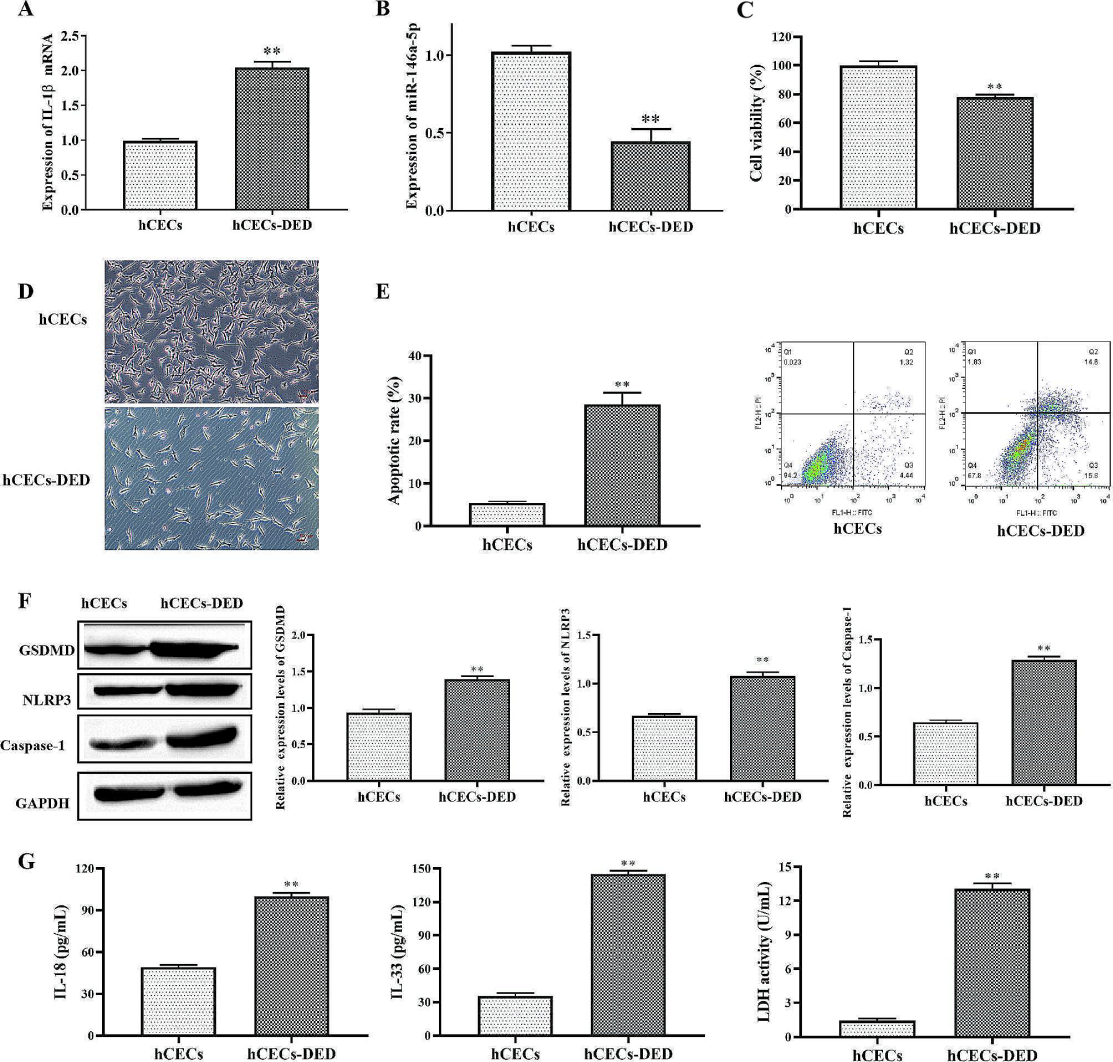
DED causes pyroptosis of hCECs. A-B RT-qPCR to measure the expression of IL-1β and miR-146a-5p; C The viability was determined by CCK-8 assay; D Microscopic observation of cell morphology and numbe; E Apoptosis was detected by flow cytometry; F Western blot analysis to measure GSDMD, NLRP3 and Caspase-1. G IL-18, IL-33 and LDH were determined by ELISA Kit. Asterisks indicate statistical significance (*p < 0.05, **p < 0.01, ***p < 0.001)

To establish the CEC DED model, hCECs were treated with 400 mOsM solutions and then labeled hCECs-DED. RT-qPCR results showed that compared with hCECs, expression IL-1β mRNA was increased in hCECs-DED (Fig. 1A), whereas miR-146a-5p were down-regulated in hCECs-DED (Fig. 1B). CCK-8 assay of cell viability revealed that treatment with 400 mOsM NaCl solution significantly inhibited cell viability (Fig. 1C). The same results were obtained by microscopic observation of cell numbers (Fig. 1D). Flow cytometry detection of apoptosis revealed a significant increase in apoptosis in the hCECs-DED group (Fig. 1E). Pyroptosis is characterized by Caspase-1-induced GSDMD-driven cell lysis and NLRP3 inflammasome activation. Therefore, we detected the pyroptosis-related proteins GSDMD, NRLP3 and Caspase-1 by Western blotting. The results showed that compared with the hCECs group, the expression of GSDMD, NRLP3 and Caspase-1 proteins in the hCECs-DED group was significantly increased (Fig. 1F). In addition, ELISA for IL-18, IL-33 and LDH concentrations showed a significant increase in IL-18 and IL-33 concentrations and LDH release in the hCECs-DED group (Fig. 1G). In summary, the above experiments showed that dry eyes increased the apoptosis of hCECs and promoted the expression of pyroptosis-related proteins.

### si-IL-1β transfection inhibits pyroptosis and apoptosis of hCECs

**Fig. 2:**
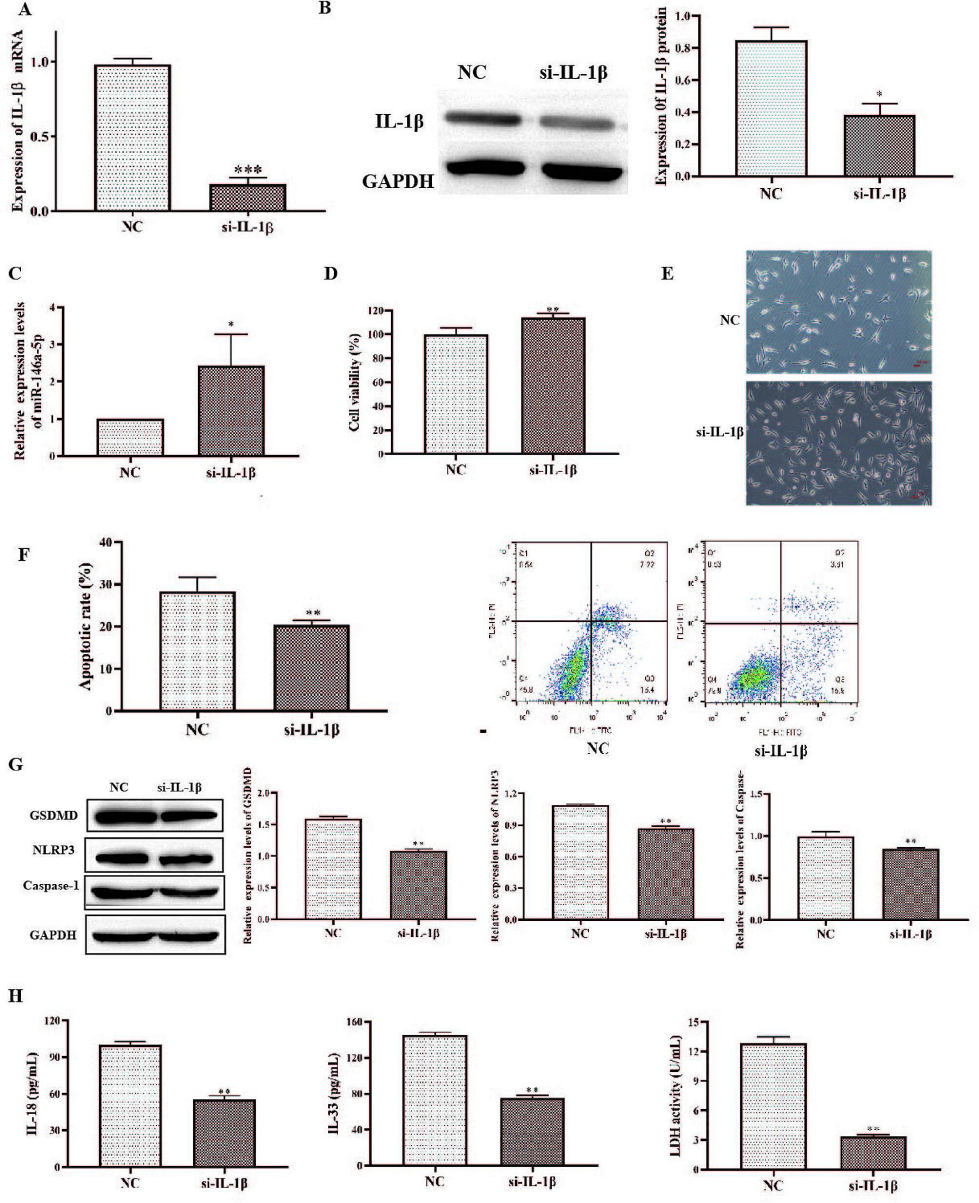
si-IL-1β transfection inhibits pyroptosis of hCECs. A RT-qPCR to measure the expression of IL-1β; B Western blot to measure the expression of IL-1β; C RT-qPCR to measure the expression of miR-146a-5p; D The viability was determined by CCK-8 assay; E Microscopic observation of cell morphology and number; F Apoptosis was detected by flow cytometry; G Western blot analysis to measure GSDMD, NLRP3 and Caspase-1; H IL-18, IL-33 and LDH were determined by ELISA Kit. Asterisks indicate statistical significance (*p < 0.05, **p < 0.01, ***p < 0.001)

To investigate whether si-IL-1β is associated with pyroptosis and apoptosis of hCECs, we transfected si-IL-1β in hCECs-DED. After transfected si-IL-1β into hCECs-DED, expression of IL-1β mRNA and protein were drastically declined in si-IL-1β group (Fig. 2A and B). Data also indicated that IL-1β silence elevated miR-146a-5p in hCECs-DED (Fig. 2C). Both CCK-8 and microscopic observation of cell numbers revealed significantly higher cell numbers in the si-IL-1β group than in the NC group (Fig. 2D and E). Cell apoptosis was detected by flow cytometry and the results showed that transfection with si-IL-1β significantly inhibited apoptosis of hCECs-DED (Fig. 2F). Similar results were obtained by Western blotting for pyroptosis-related proteins (Fig. 2G). Measurement of inflammatory factor (IL-18, IL-33) concentrations and LDH release revealed a decrease in inflammatory factor concentrations and relief of cellular damage (Fig. 2H). Therefore, the ability of IL-1β to affect pyroptosis and apoptosis of hCECs-DED.

### IL-1β effected on pyroptosis and apoptosis of hCECs-DED through miR-146a-5p

**Fig. 3:**
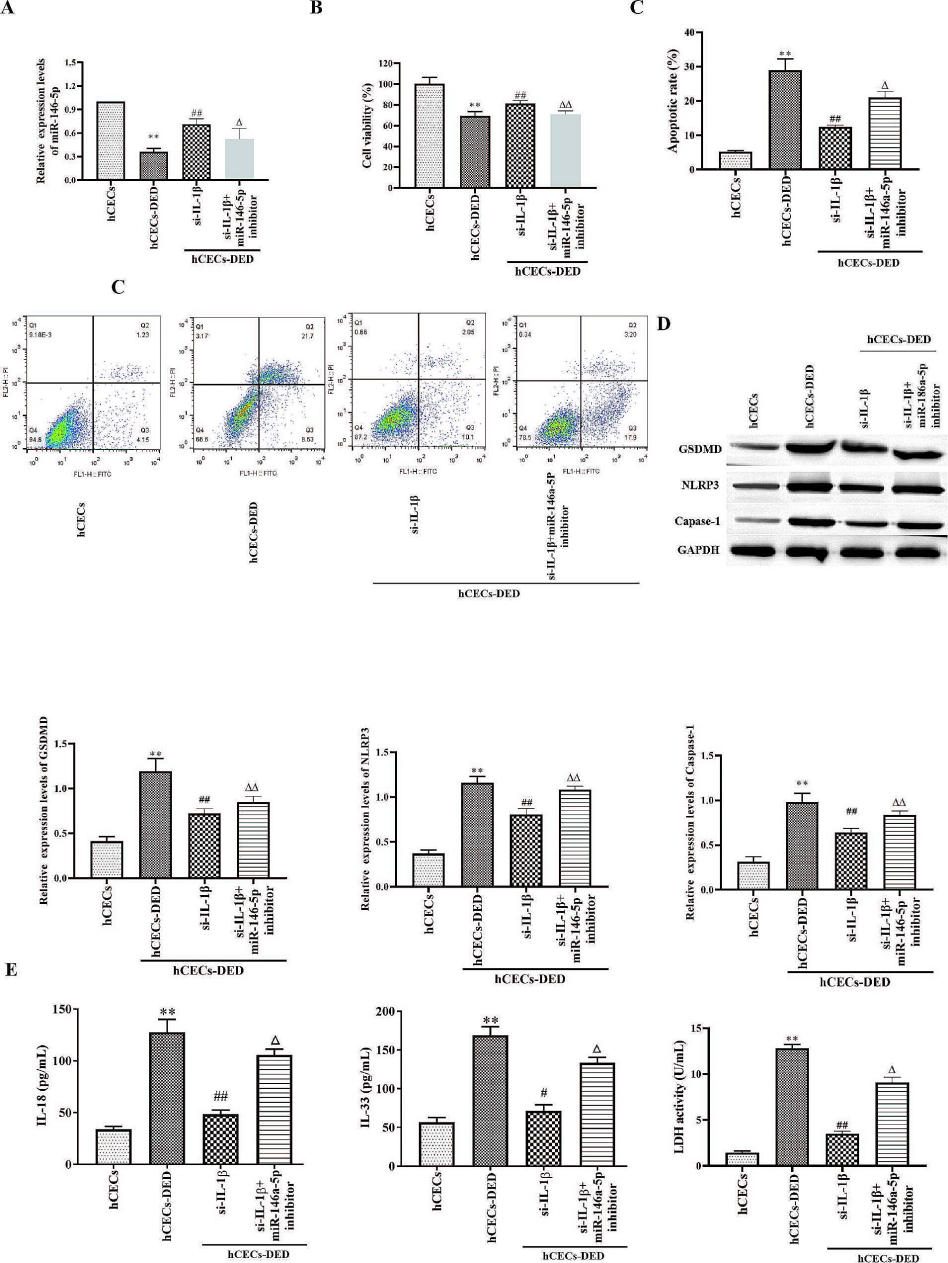
IL-1β effected on pyroptosis of hCECs-DED through miR-146a-5p. A RT-qPCR to measure the expression of miR-146a-5p; B The viability was determined by CCK-8 assay; C Apoptosis was detected by flow cytometry; D Western blot analysis to measure GSDMD, NLRP3 and Caspase-1. E IL-18, IL-33 and LDH were determined by ELISA Kit. Compared with hCECs, *p < 0.05, **p < 0.01; Compared with NC, #*p* < 0.05, ##*p* < 0.01; Compared with si-IL-1β, #*p* < 0.05, ##*p* < 0.01

RT-qPCR data demonstrated that miR-146a-5p was markedly decreased in si-IL-1β and miR-146a-5p inhibitor group compared with si-IL-1β group (Fig. 3A). CCK-8 results suggested that compared with hCECs, cell viability memorably decreased in hCECs-DED, while IL-1β silence raised cell viability of hCECs-DED. However, cell viability of hCECs-DED in si-IL-1β + miR-146a-5p inhibitor treatment was suppressed compared to si-IL-1β treatment (Fig. 3B). Apoptosis results showed that miR-146a-5p inhibitor treatment significantly inhibited the effect of si-IL-1β (Fig. 3C). The same effect was found for Western blotting detection of pyroptosis-related proteins (Fig. 3D). ELISA results indicated that IL-18, IL-33 and LDH substantially increased in hCECs-DED group compared with hCECs, IL-1β silence decreased IL-18, IL-33 and LDH, but the levels of IL-18, IL-33 and LDH were higher in si-IL-1β + miR-146a-5p inhibitor treatment compared with si-IL-1β group (Fig. 3E). Thus, pyroptosis and apoptosis existed in hCECs-DED, IL-1β silence inhibited pyroptosis and apoptosis of hCECs-DED, while miR-146a-5p inhibitor abolished IL-1β silence caused suppression of hCECs-DED pyroptosis and apoptosis.

### Targeting relationship between miR-146a-5p and STAT3

**Fig. 4:**
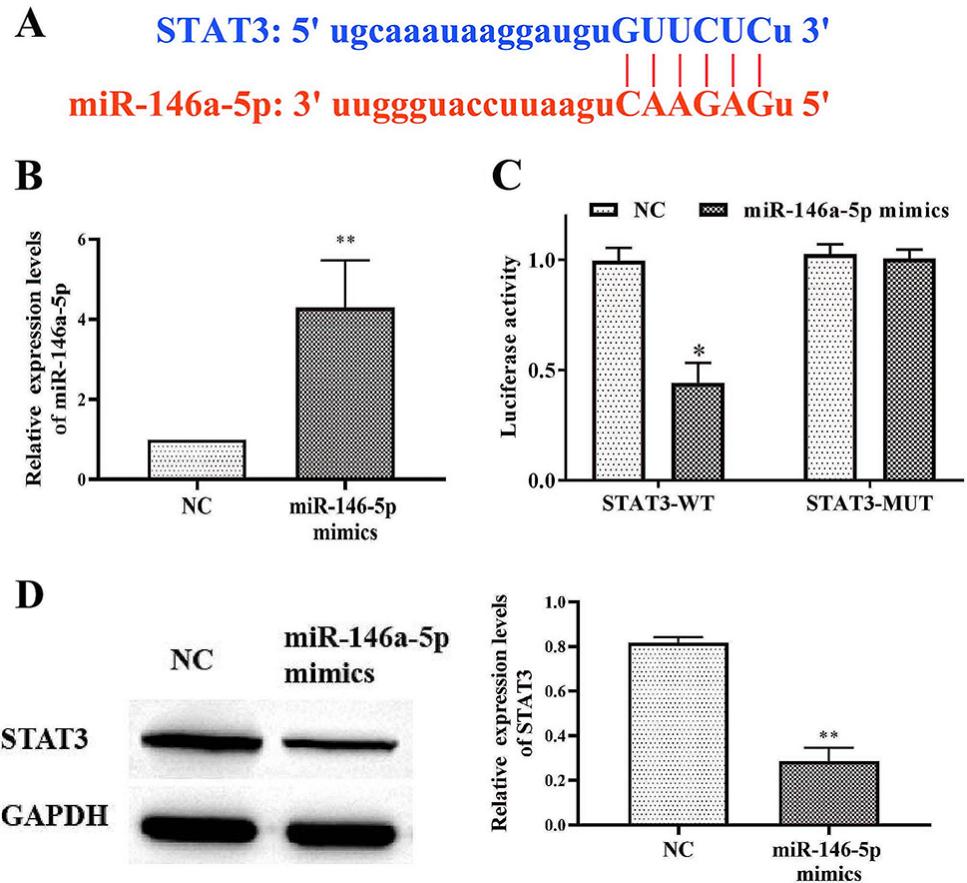
Targeting relationship between miR-146a-5p and STAT3. A Binding sites of miR-146a-5p in STAT3; B RT-qPCR to measure the expression of miR-146a-5p; C Luciferase was quantified by Nano-Glo® Dual-Luciferase® System (Promega) 48 h later; D Western blots and quantification of STAT3. Asterisks indicate statistical significance (*p < 0.05, **p < 0.01, ***p < 0.001)

The binding sites of miR-146a-5p in STAT3 were shown in Fig. 4A. To verify their relationship, miR-146a-5p mimics were introduced into hCECs (Fig. 4B). Luciferase assay results showed that miR-146a-5p mimics significantly inhibited STAT3-WT but not STAT3-MUT activity compared to hCECs-400 mOsM (Fig. 4C). Moreover, miR-146a-5p mimics dramatically suppressed STAT3 in hCECs (Fig. 4D). Therefore, miR-146a-5p inhibited STAT3 in hCECs.

### IL-1β/miR-146a-5p/STAT3 influenced pyroptosis and apoptosis of hCECs-DED

**Fig. 5:**
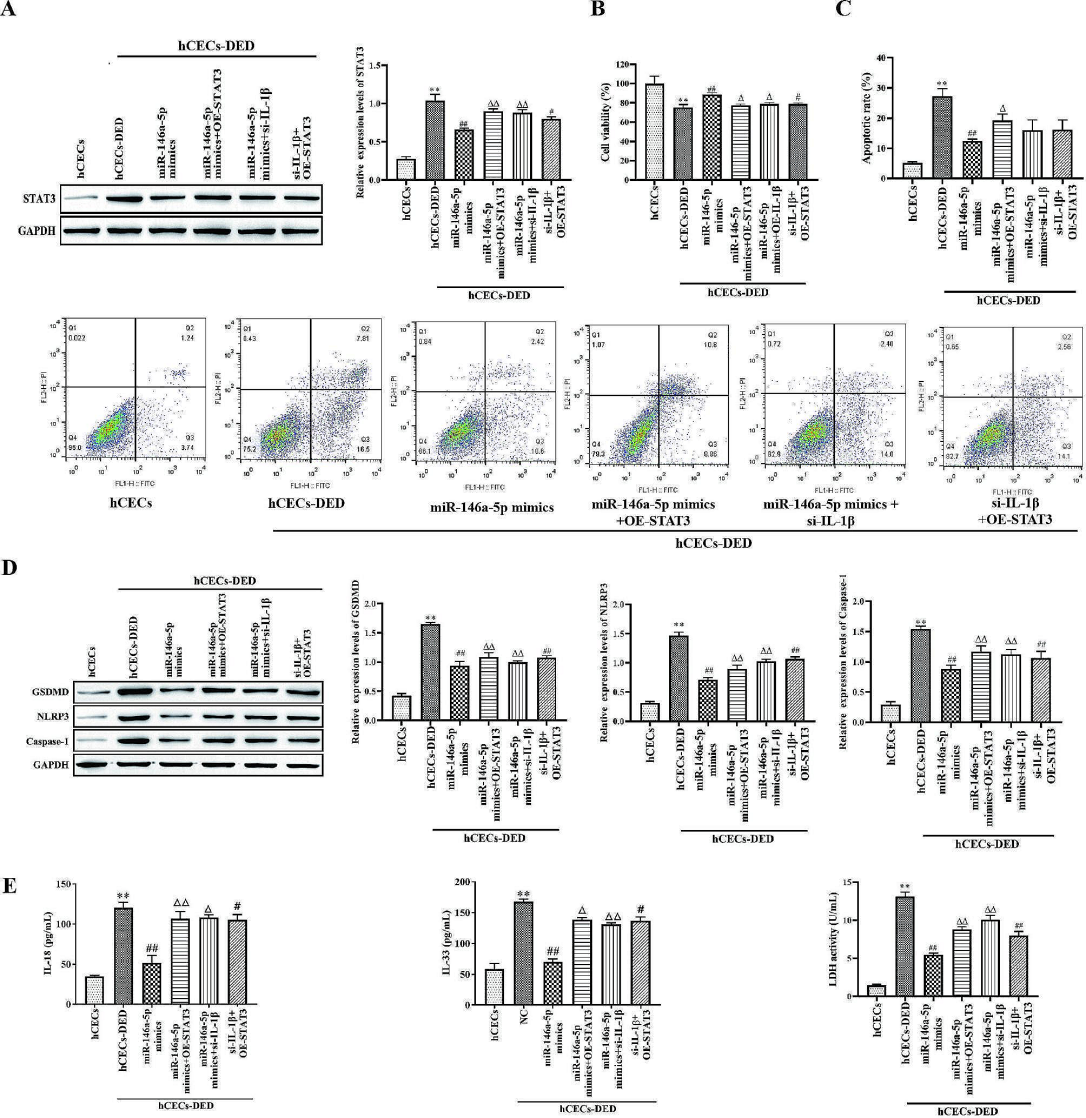
IL-1β/miR-146a-5p/STAT3 influenced pyroptosis of hCECs-400 mOsM. A Western blot analysis to measure STAT3; B The viability was determined by CCK-8 assay; C Apoptosis was detected by flow cytometry; D Western blot analysis to measure GSDMD, NLRP3 and Caspase-1; E IL-18, IL-33 and LDH were determined by ELISA Kit. Compared with hCECs, *p < 0.05, **p < 0.01; Compared with NC, #*p* < 0.05, ##*p* < 0.01; Compared with miR-146a-5p mimics, #*p* < 0.05, ##*p* < 0.01

To verify that IL-1β can mediate miR-146a-5p/STAT3 to affect pyroptosis and apoptosis of hCECs-DED. Western blotting indicated that STAT3 was increased in hCECs-DED compared with hCECs, it was inhibited by miR-146a-5p overexpression, while this inhibition reversed by STAT3 overexpression or IL-1β treatment (Fig. 5A). Cell viability as measured by CCK-8 indicated that miR-146a-5p mimics promoted cell viability compared to hCECs-DED, and OE-STAT3 or si-IL-1β treatment inhibited this promotion (Fig. 5B). The results of flow cytometric detection of apoptosis were opposite to those of CCK-8 (Fig. 5C). Western blot detection of pyroptosis-related proteins revealed that miR-146a-5p mimics significantly inhibited the expression of pyroptosis-related proteins, while miR-146a-5p mimics + OE-STAT3 or miR-146a-5p mimics + si-IL-1β treatment was able to promote the expression of pyroptosis-related proteins compared with miR-146a-5p mimics (Fig. 5D). Meanwhile, trends in IL-18, IL-33 and LDH content were the same as those of pyroptosis-related proteins (Fig. 5E). Consequently, IL-1β promoted pyroptosis and apoptosis of hCECs-DED through downregulated miR-146a-5p and inhibited STAT3.

## Discussion

DED is a major ophthalmic disease worldwide, characterized by loss of tear film homeostasis leading to eye discomfort and even visual impairment, which seriously affects the quality of life of patients [19]. A growing number of studies suggest that chronic immune processes play a key role in the pathogenesis of DED, characterized by elevated levels of pro-inflammatory cytokines and inflammation, which further leads to disruption of the corneal epithelial barrier [20]. Tear hyperpermeability leads to symptoms of dry eye affecting epithelial cell function. The osmolality of normal subjects ranged from 290 to 330 mOsm / L, while the osmolality of dry eye patients ranged from 315 to 365 mOsm/L [21]. Studies have shown that the use of 350–500 mOsM hypertonic medium to construct hCECs-DED [22].In our study, 400 mOsM hypertonic medium modeling effect is better. At the same time, this study found that high osmotic pressure can cause pyroptosis and apoptosis of hCECs (Fig. 1).

Pyroptosis is also known as cellular inflammatory necrosis and is a programmed cell death mediated by caspase-1. Caspase-1 is instrumental in the process of transforming two proinflammatory cytokines, IL-1β and IL-18, into mature forms within the inflammasome pathway. Caspase-1 also cleaves the pore-forming protein GSDMD into its active form N-GSDMD, which triggers a cascade of events that culminates in plasma membrane rupture and intracellular content release, resulting in a localized or systemic inflammatory response [23]. IL-1β is a key mediator of the inflammatory response [24]. It is recognized that inflammation such as high levels of IL-1β, is an underlying cause of DED. On the other hand, pyroptosis also leads to an increase in the expression of IL-1β [25, 26]. There were many researches demonstrated that IL-1β was up-regulated in DED patients [9, 27, 28]. In addition to pyroptosis, caspase-1 is also involved in apoptosis in inflammatory situations [29]. Our results showed that IL-1β was highly expressed in DED model hCECs, and IL-1β silencing inhibited the thermal degeneration of DED model hCECs (Fig. 2). Therefore, high level of IL-1β in DED patients promoted pyroptosis and apoptosis of hCECs, and then pyroptosis hCECs released pro-inflammatory cytokines that further worsen the condition of DED. This process formed an “inflammatory vicious cycle” in DED.

miRNAs are involved in a variety of physiological processes in cells. miR-146a-5p is a key regulator of inflammatory responses. In addition, miR-146a-5p is associated with a variety of diseases, such as cancer [30], renal ischemia/reperfusion injury [31], intracranial aneurysms [32], sjögren’s syndrome [33]. One study found that miR-146a-5p expression was reduced in dry eye patients [34]. In the present study miR-146a-5p was also found to be significantly reduced in hCECs-DED. The use of miR-146a-5p mimics treatment can increase cell viability and inhibit the occurrence of inflammation (Fig. 3).

STAT3 was identified as a target gene of miR-146a-5p by bioinformatics analysis, while luciferase assay and protein blotting confirmed this [35]. Signal transducer and STAT3 is a latent cytoplasmic protein associated with inflammation and cellular pyroptosis [36, 37]. One of the pathogenic mechanisms of Yorkren syndrome is STAT3-mediated epithelial cell dysfunction [38]. In addition, recent studies have found that IL-1β regulates wound healing in the corneal epithelium via p16Ink4a-STAT3 signaling [39]. STAT3 inhibitor ameliorates dry eye symptoms in mice [40]. In this study, we found that miR-146a-5p significantly decreased STAT3 expression (Fig. 4). Overexpression of STAT3 significantly inhibited the effects of miR-146a-5p on the expression of inflammatory factors and the proliferation of hCECs and pyroptosis and apoptosis (Fig. 5).

## Conclusion

In summary, this study found that IL-1β promotes pyroptosis and apoptosis in the DED model by downregulating miR-146a-5p and promoting STAT3. Our findings could provide a theoretical basis for the treatment of DED.

### Electronic supplementary material

Below is the link to the electronic supplementary material.

Supplementary Material 1

## Data Availability

All data is real and guarantee the validity of experimental results. If you need the results you can send an email (18987464191@163.com) to the corresponding authors.
