# Supplementary material for: IL-1β induced down-regulation of miR-146a-5p promoted pyroptosis and apoptosis of corneal epithelial cell in dry eye disease through targeting STAT3
Source: BMC Ophthalmol. 2024 Mar 29;24:144. doi: 10.1186/s12886-024-03396-8 (PMC10981279; doi:10.1186/s12886-024-03396-8)

Figure 1F NLRP3









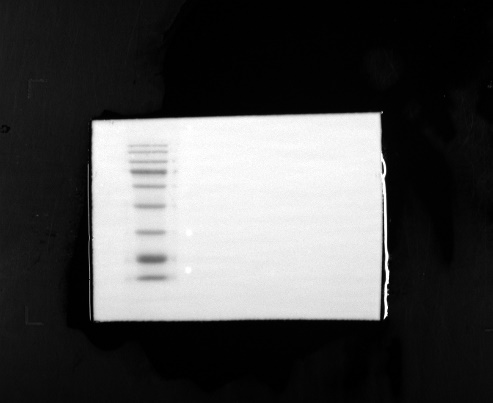


Figure 1F GSDMD


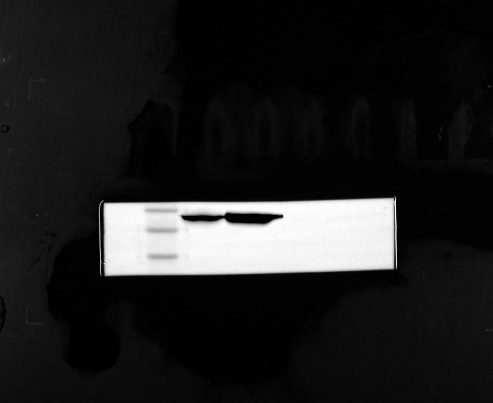




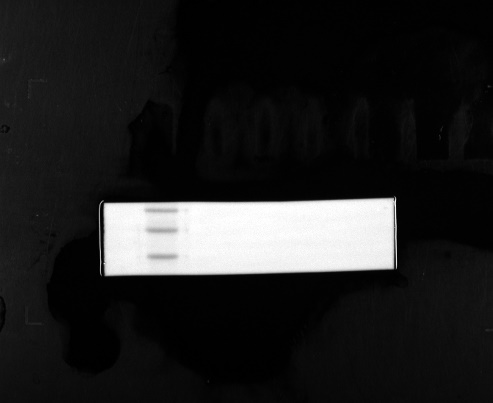

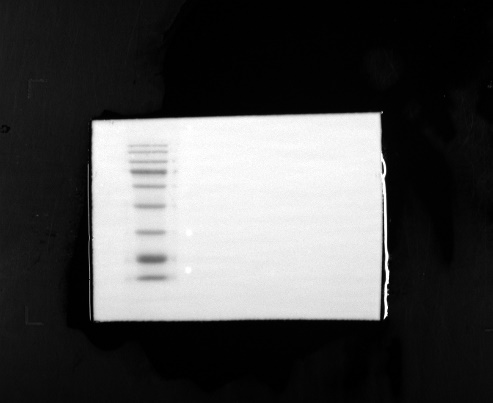


Figure 1F GAPDH









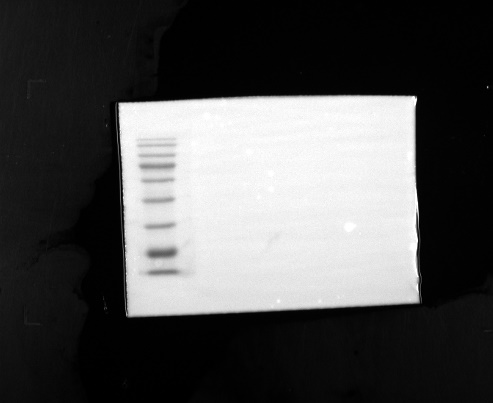


Figure 1F Capase-1









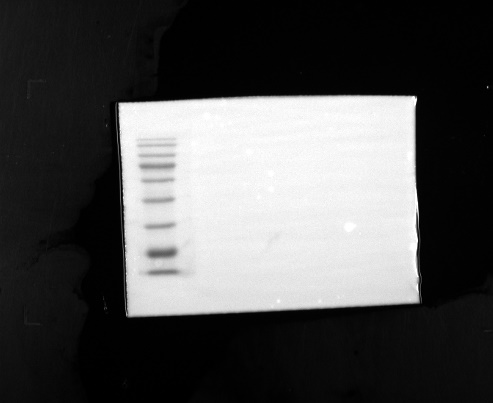


Figure 2B IL-β


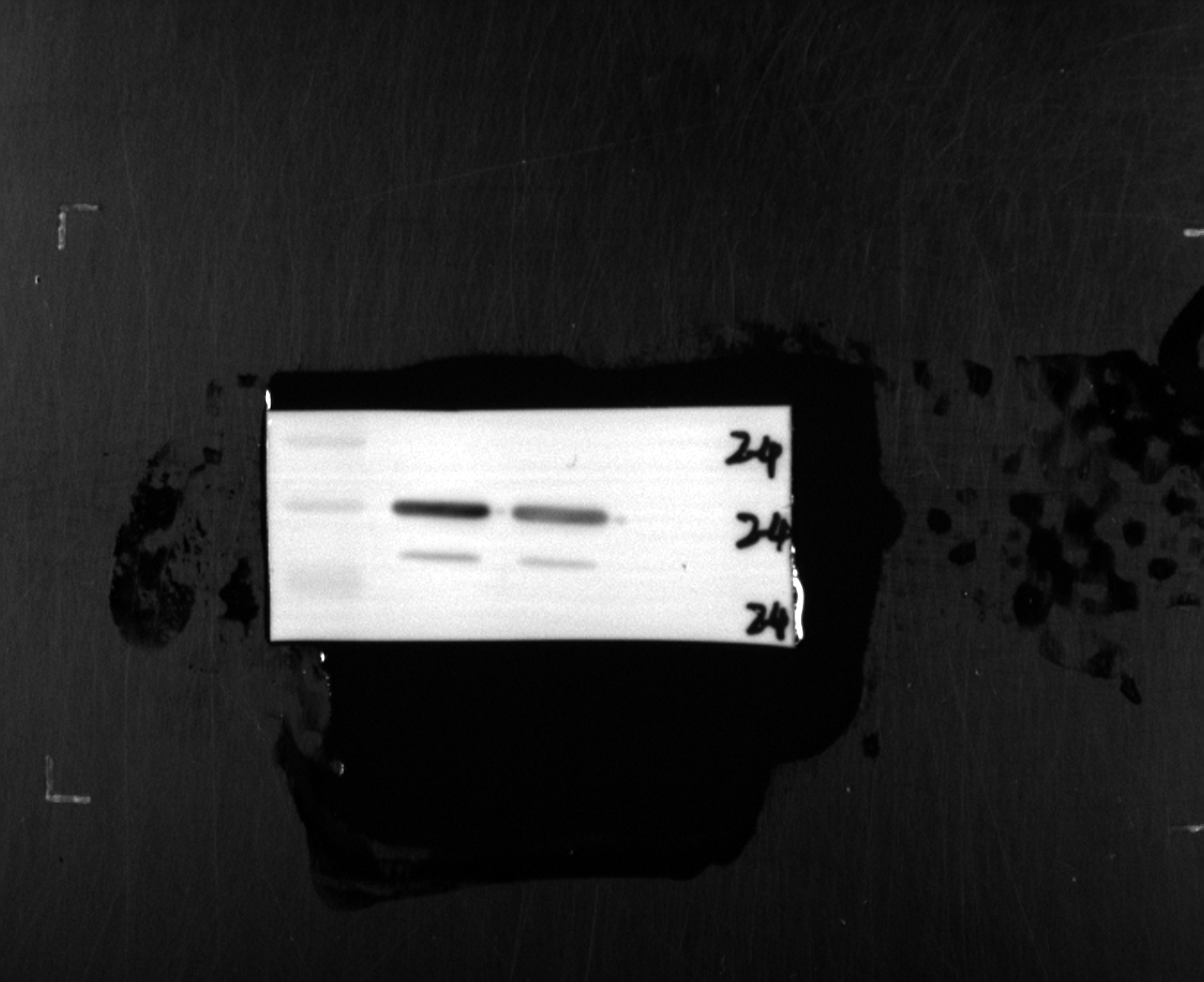

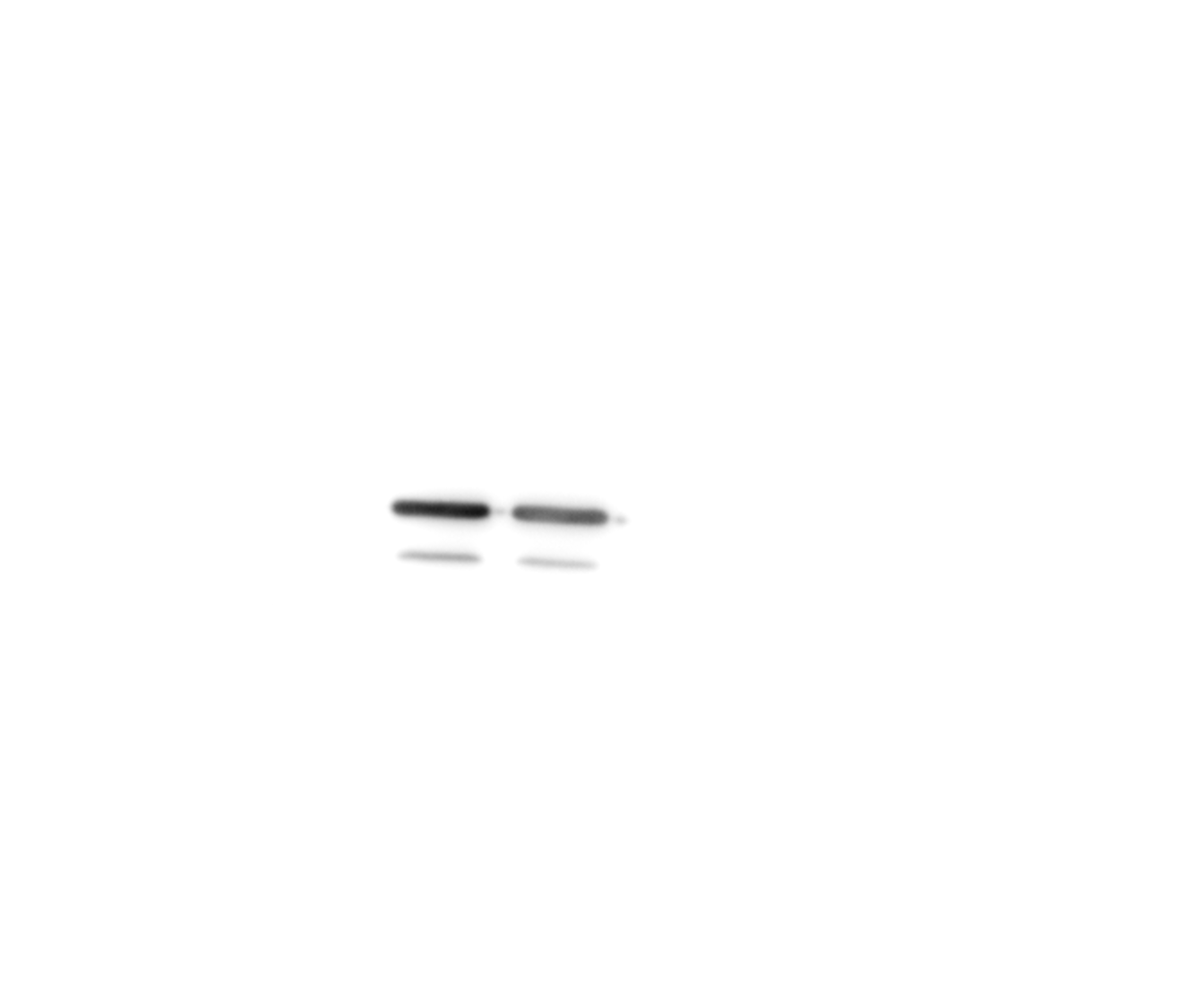

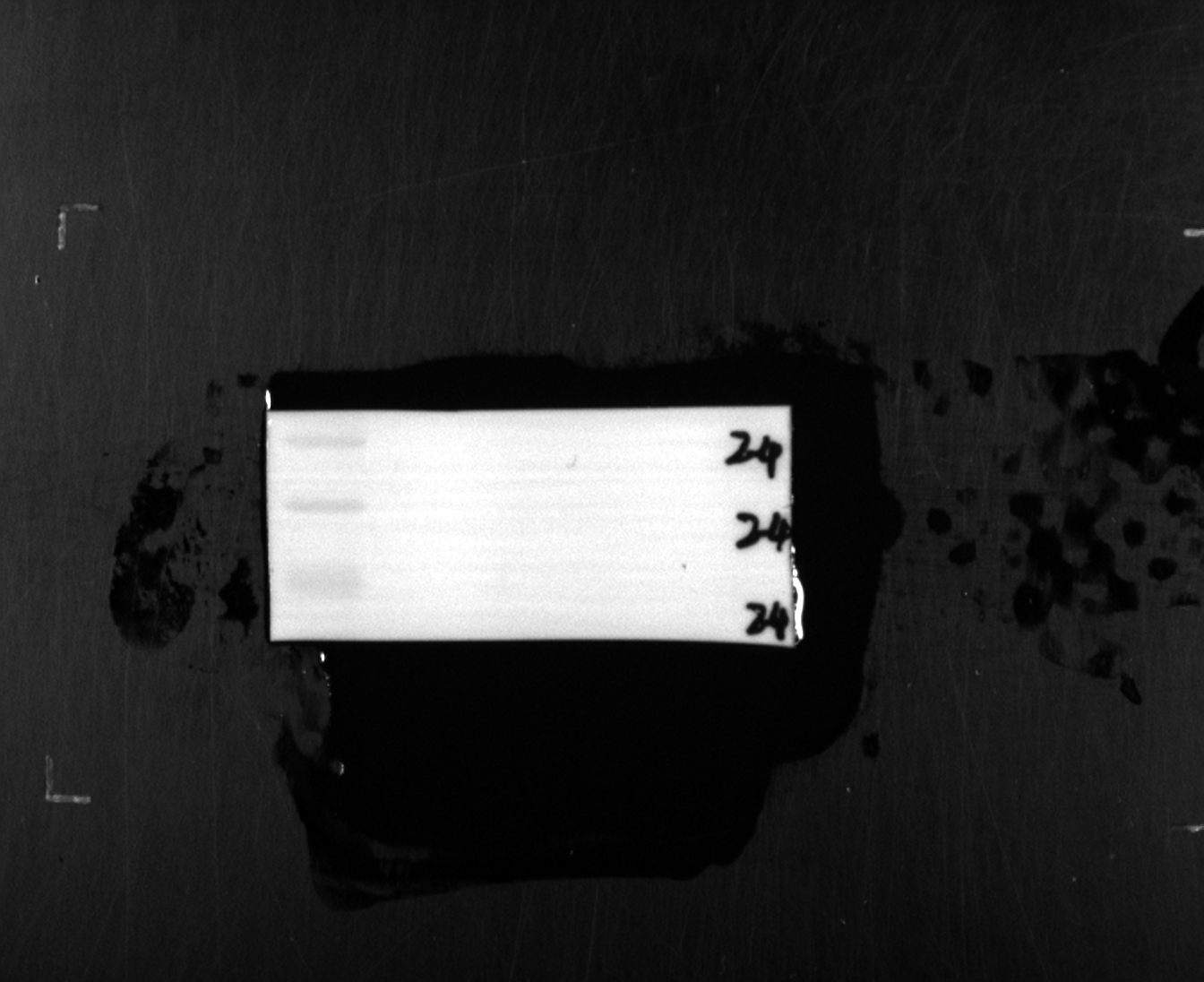

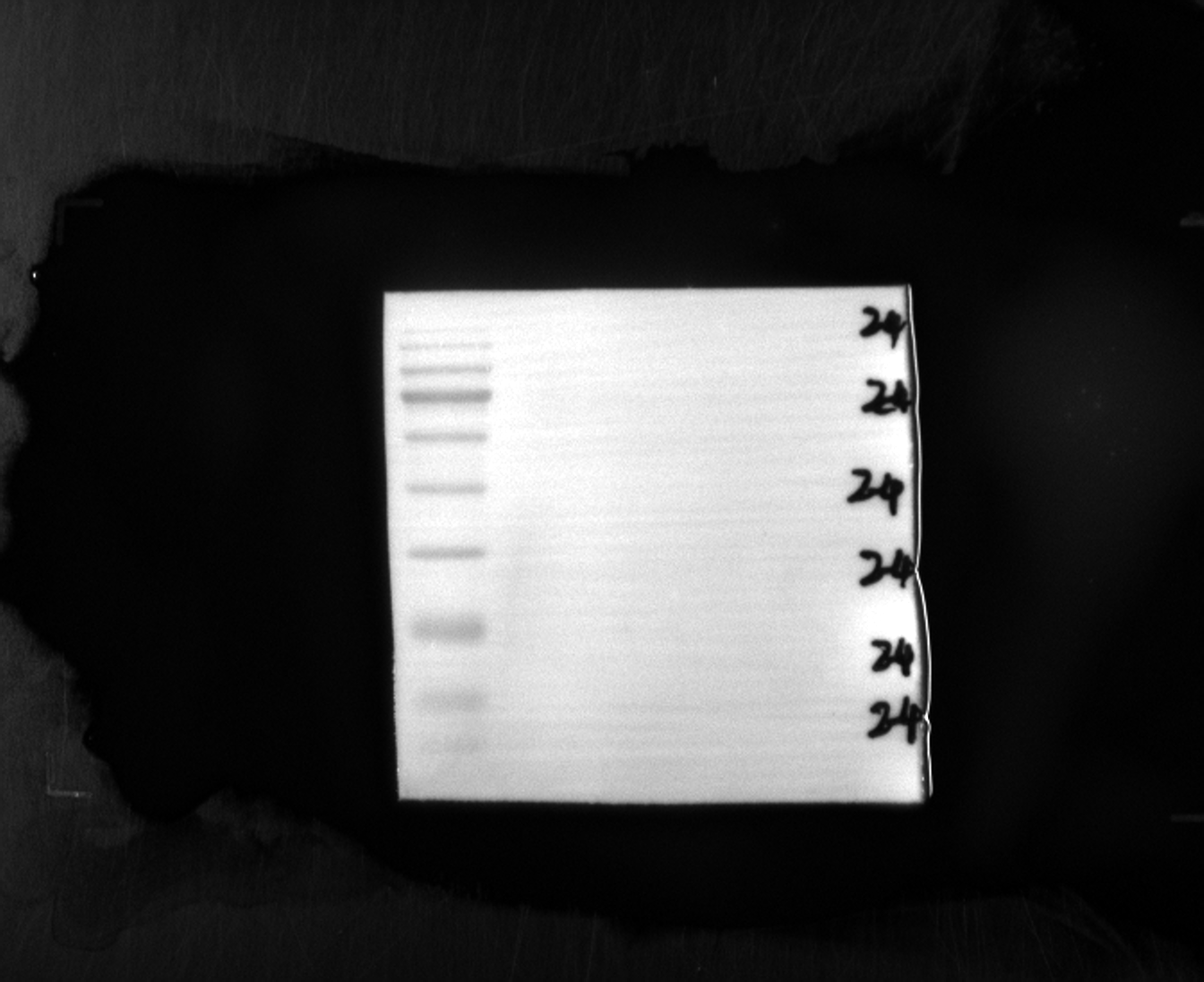


Figure 2B GAPDH


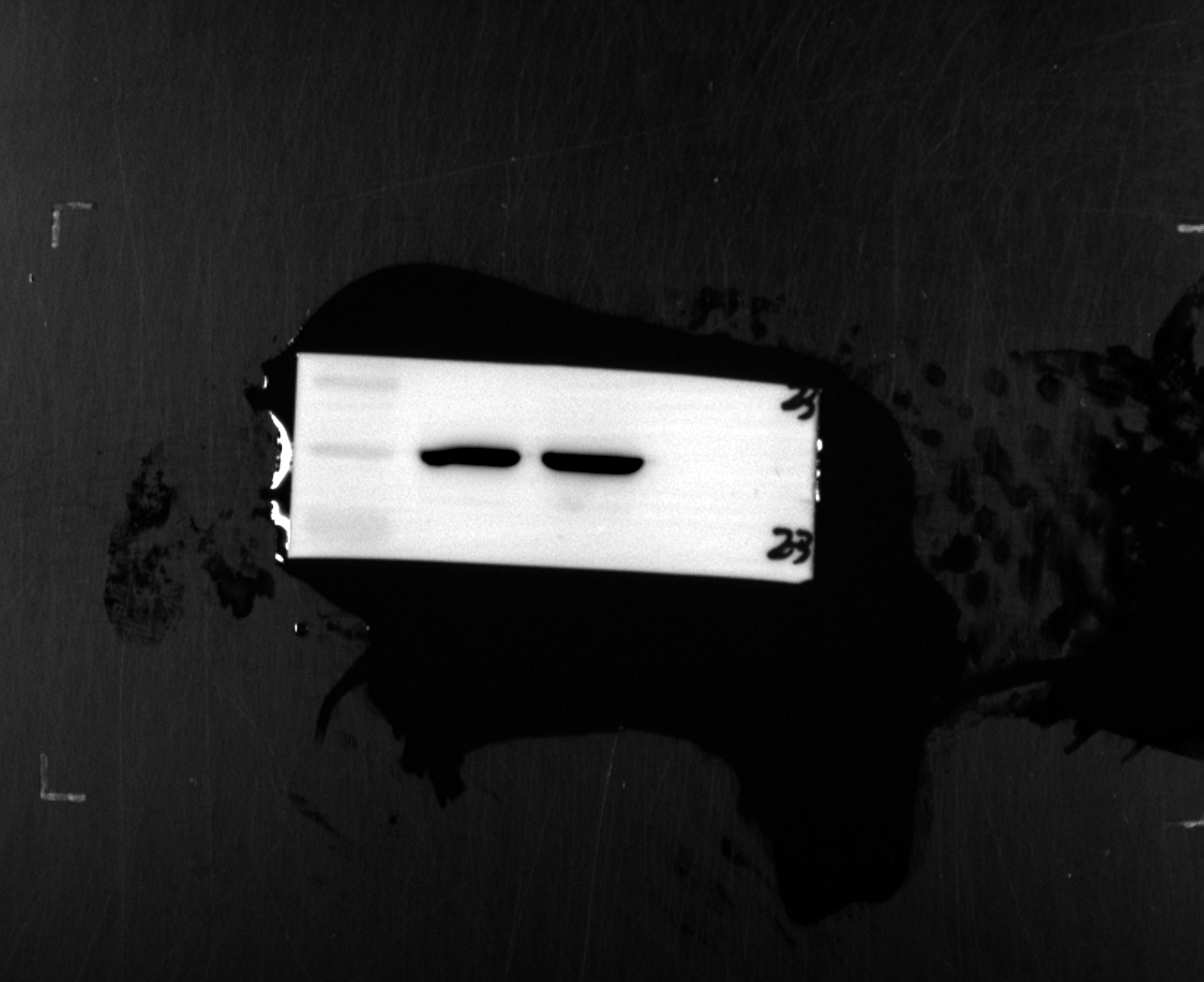

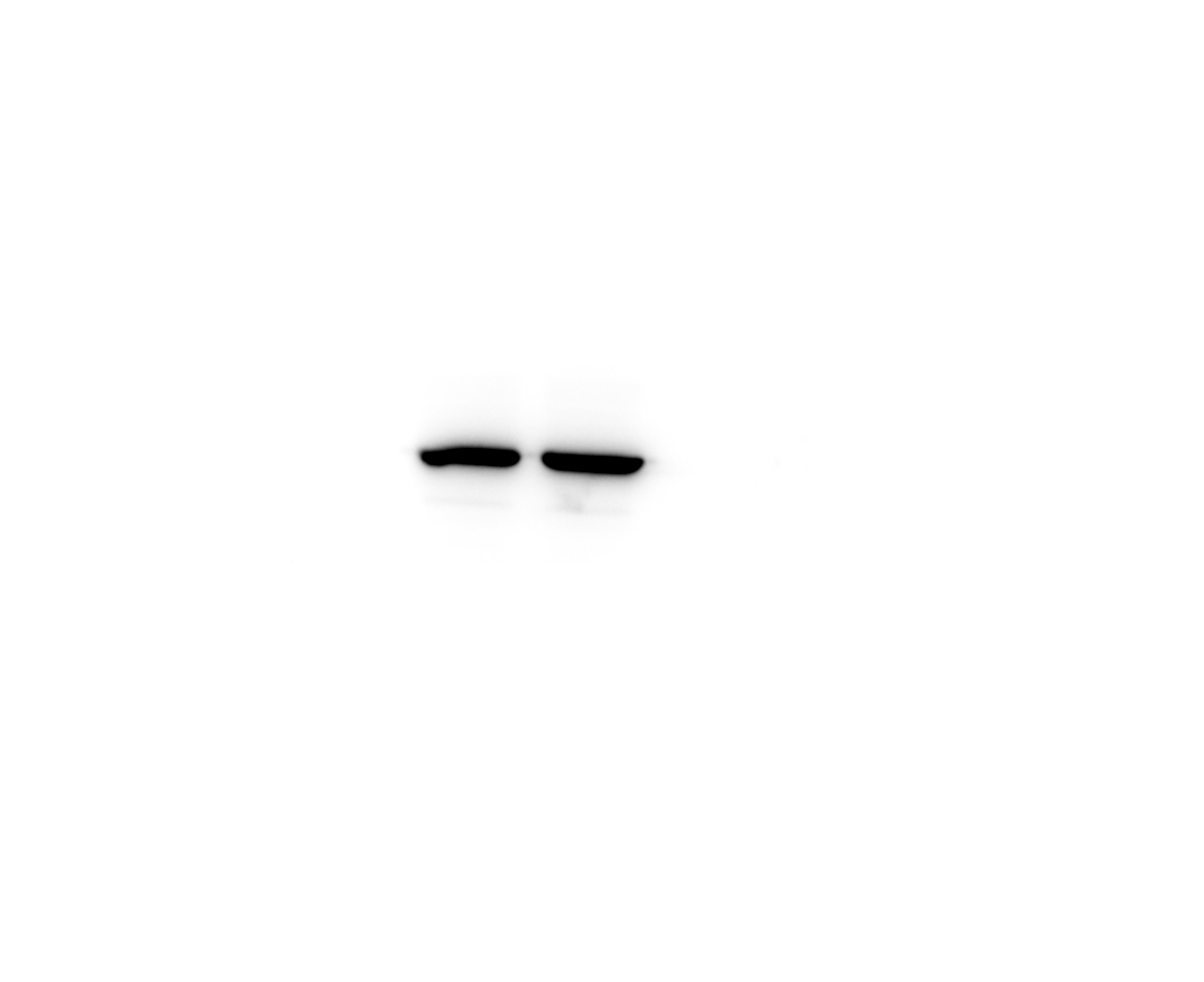

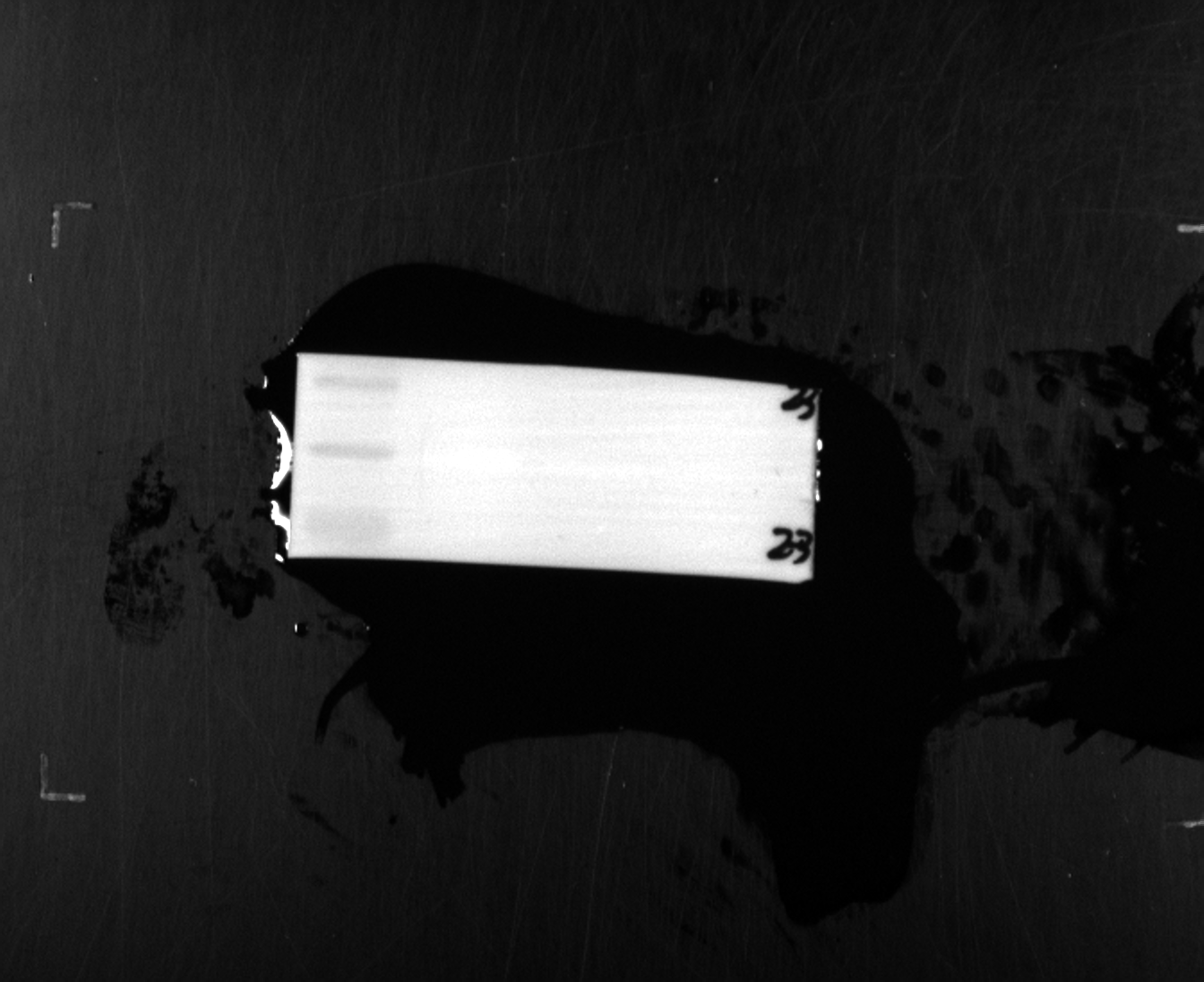

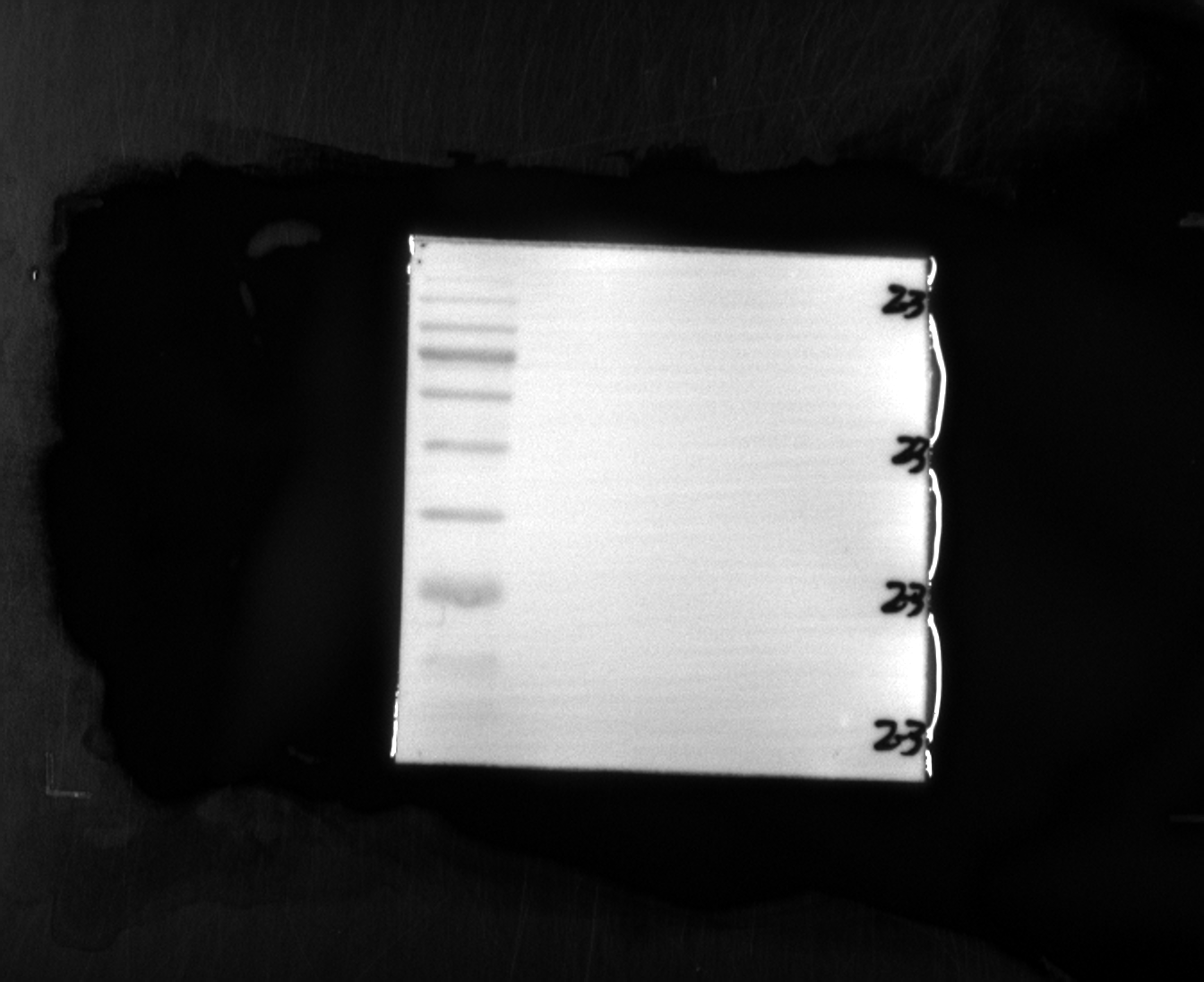


Figure 2G NLRP3


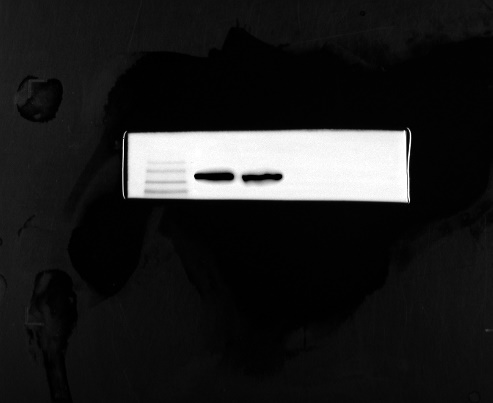




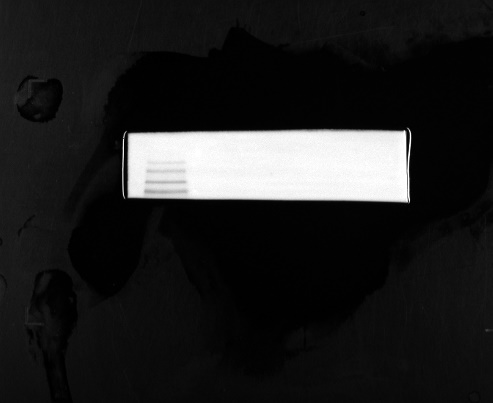

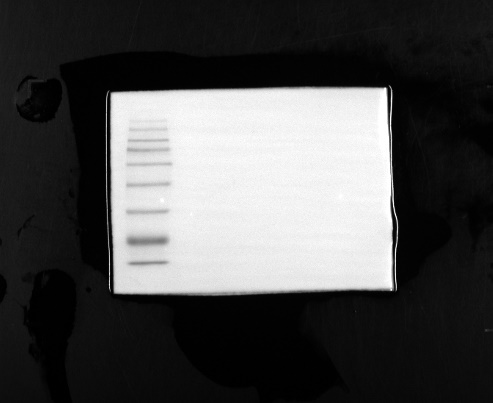


Figure 2G GSDMD









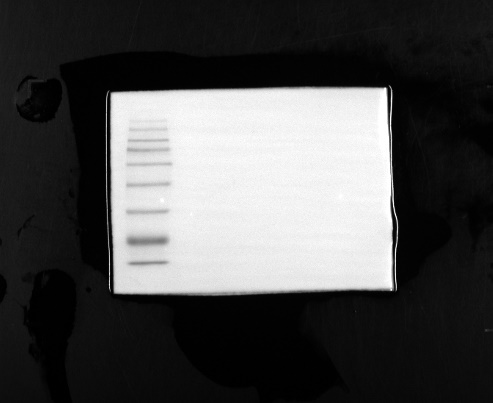


Figure 2G GAPDH


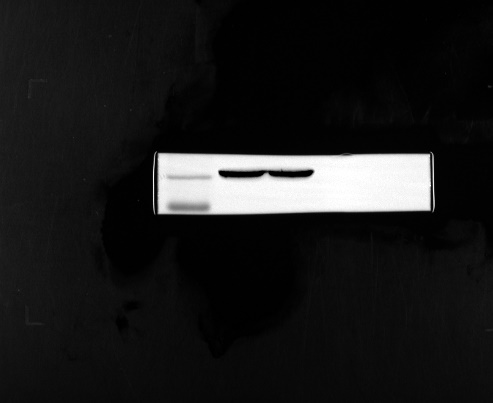




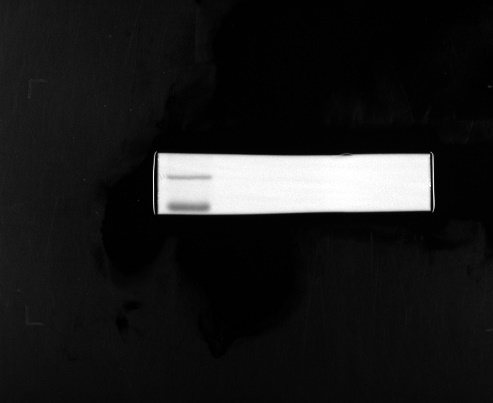

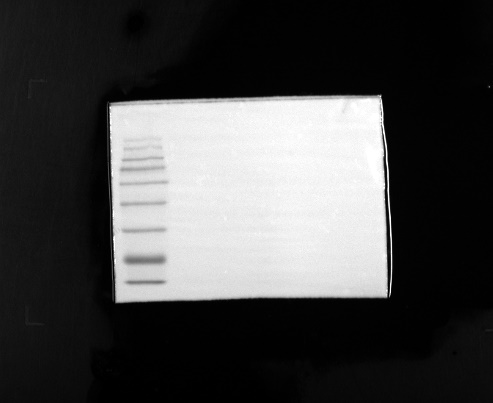


Figure 2G Caspase-1









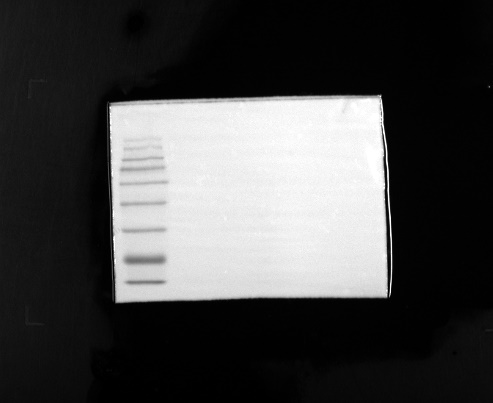


Figure 3D NLRP3


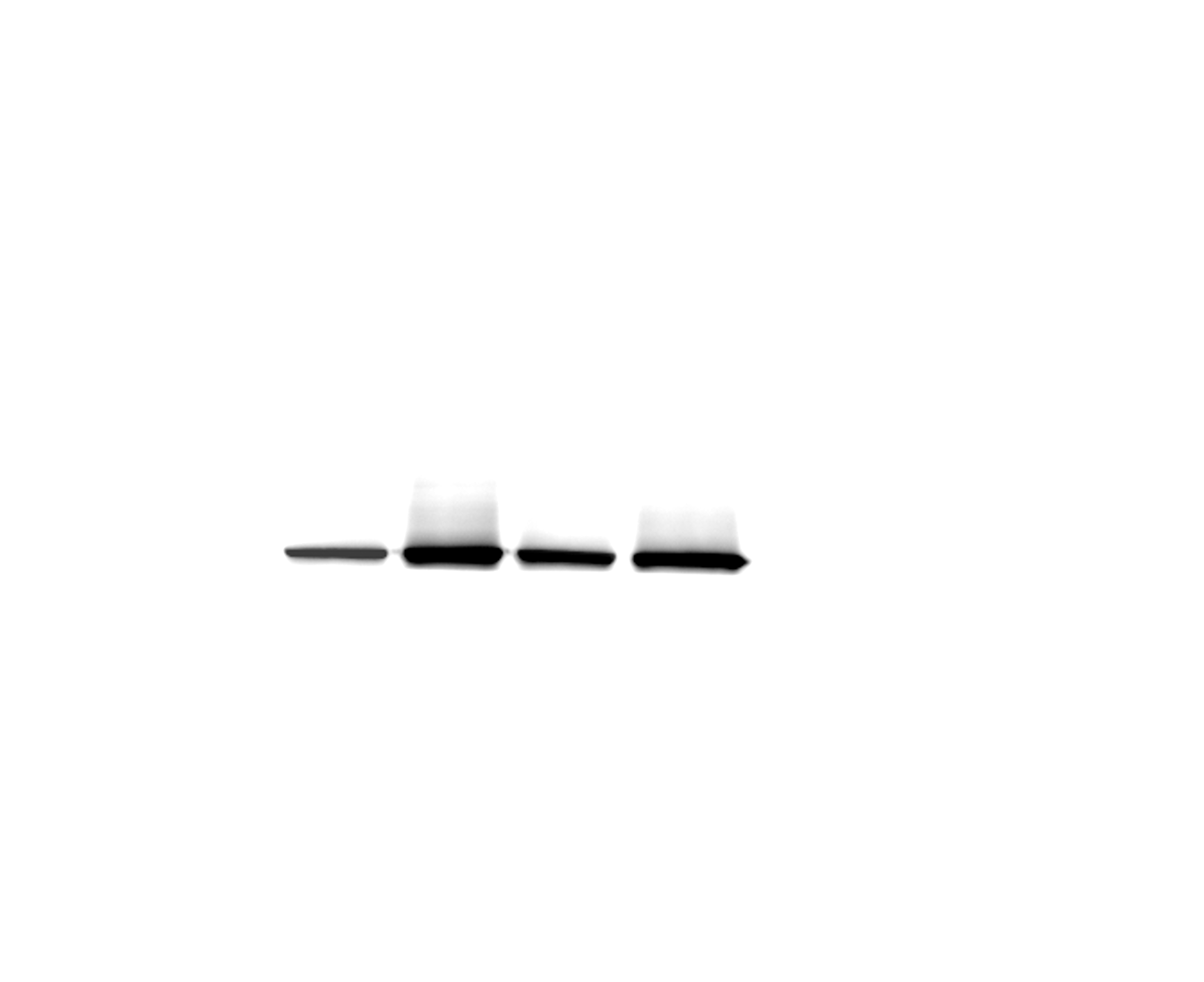

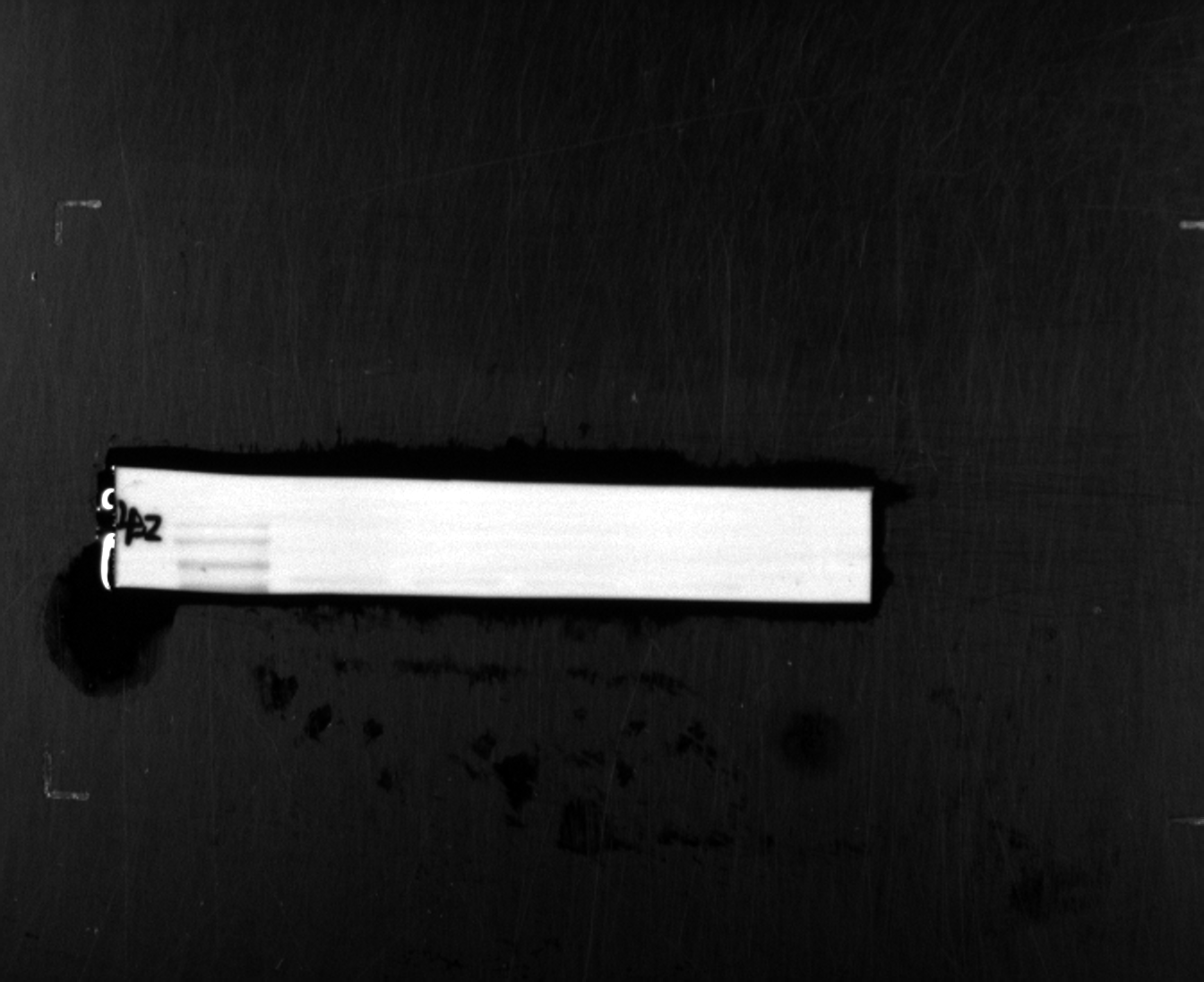

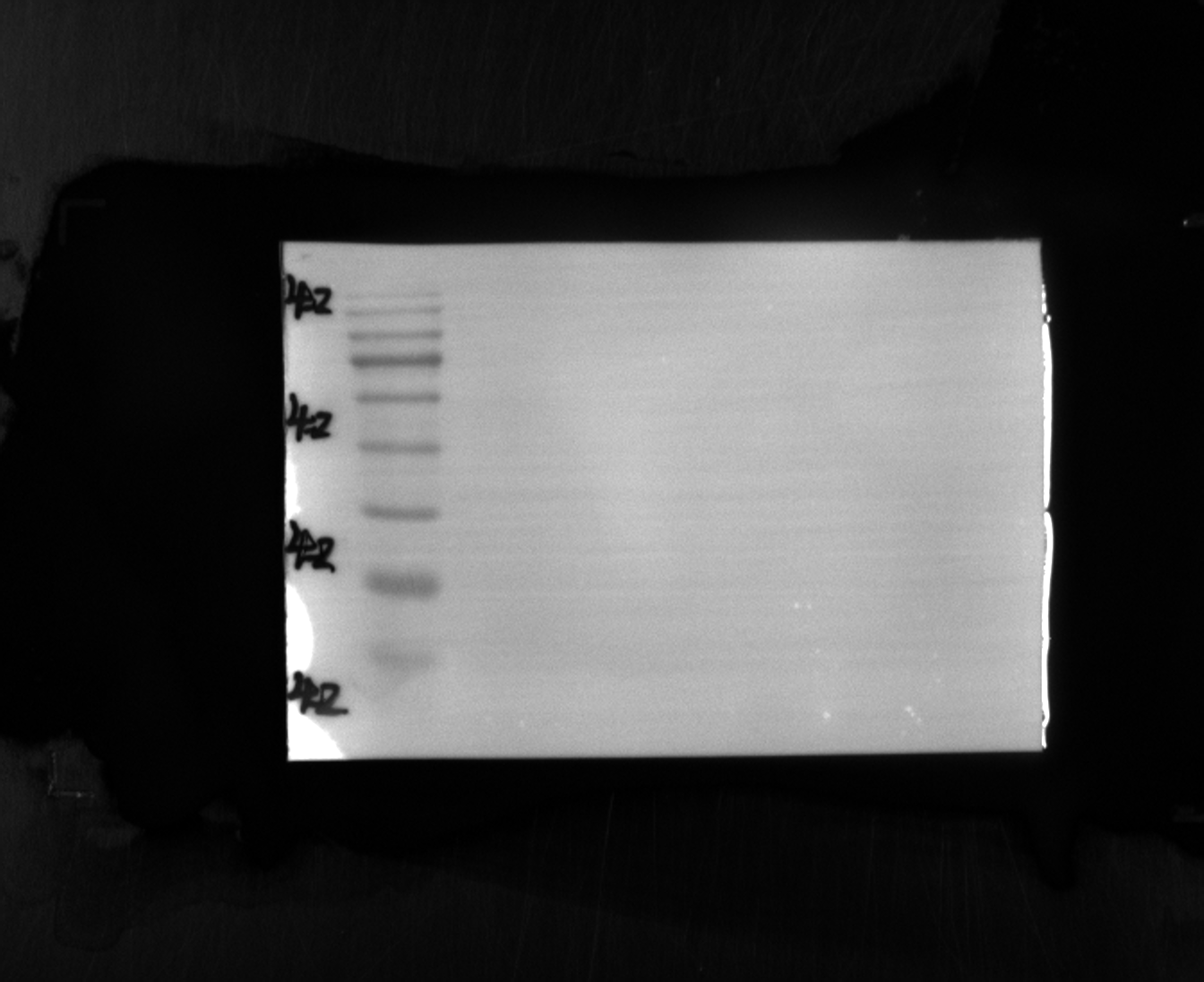

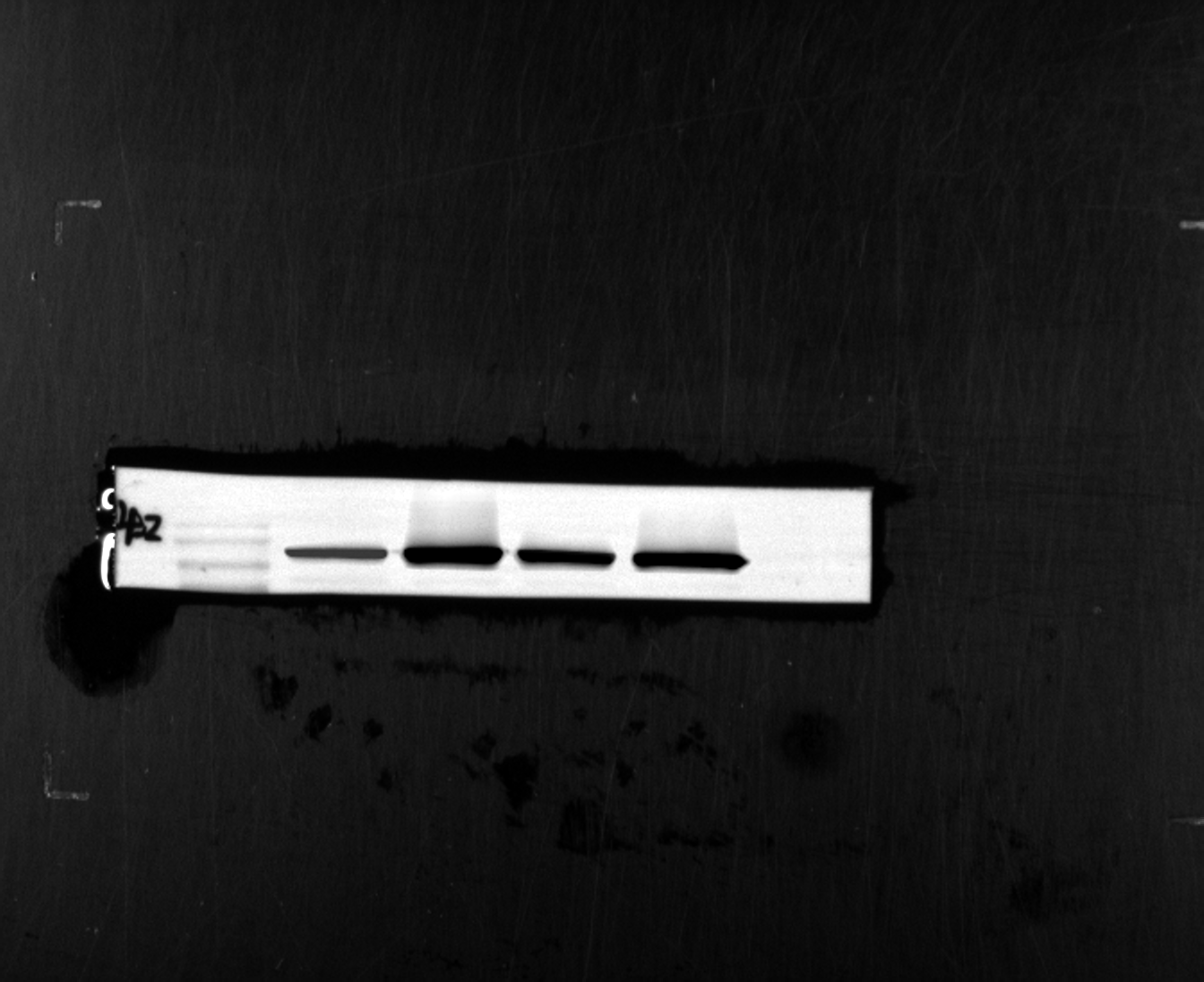


Figure 3D GSDMD


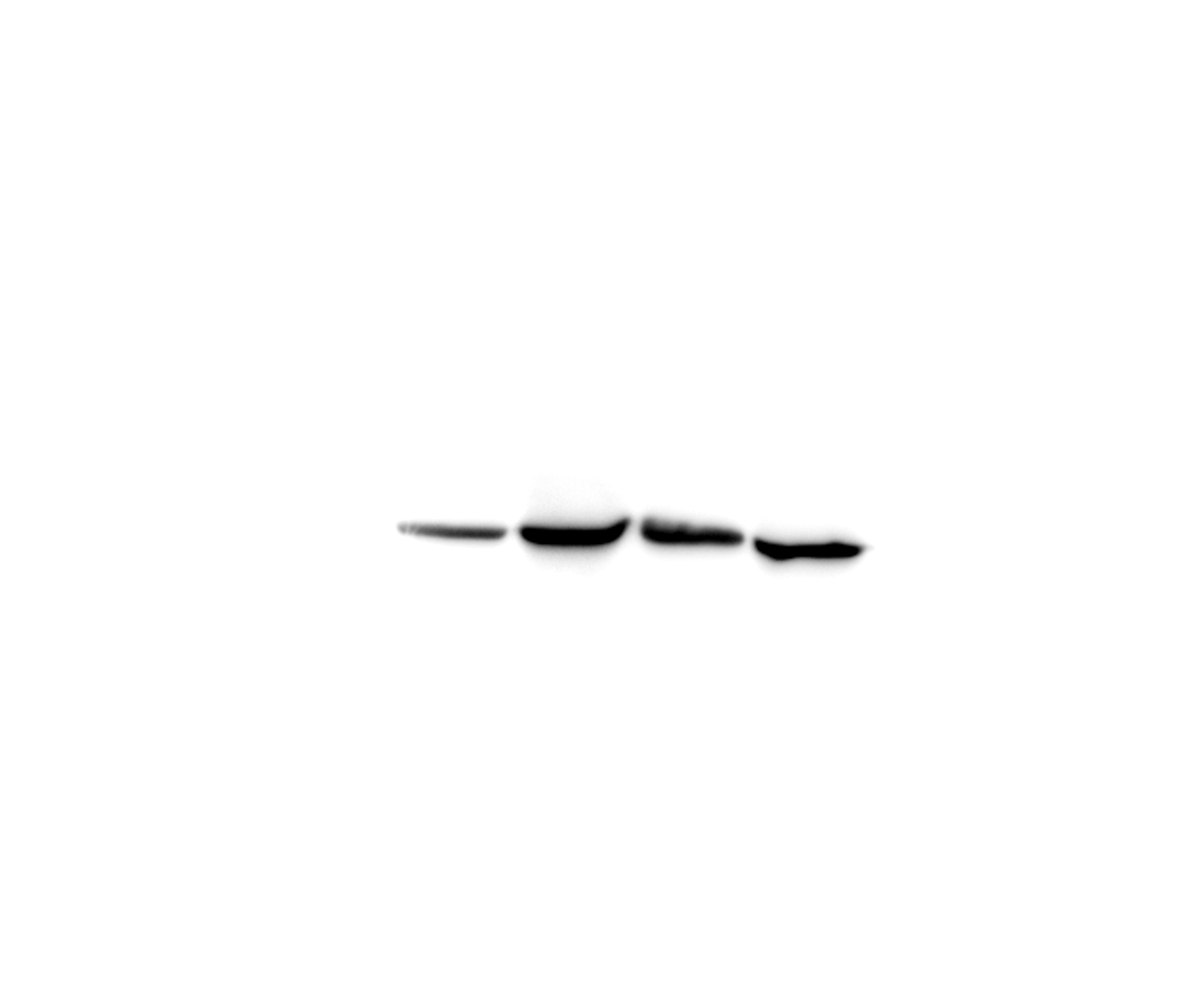

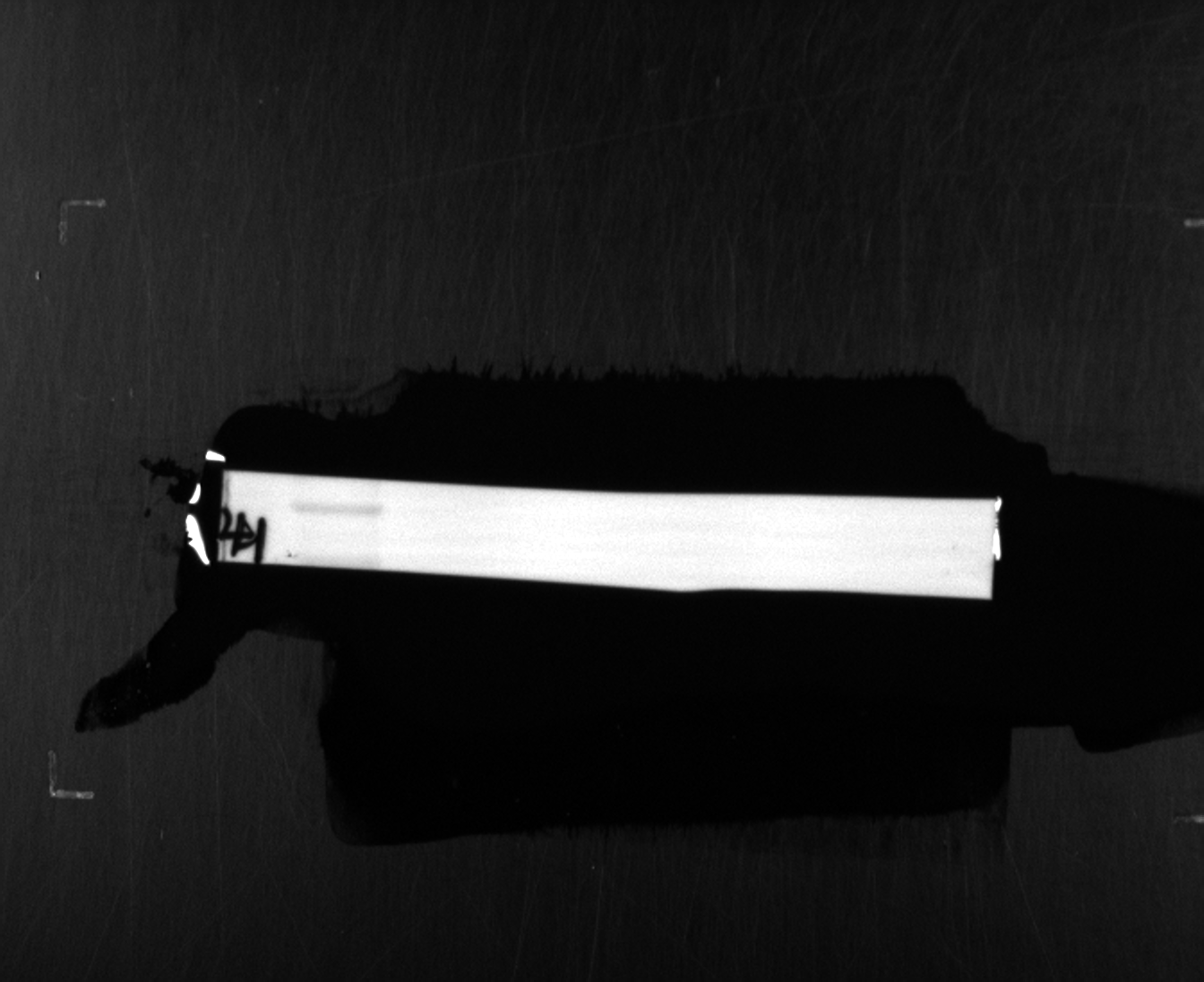

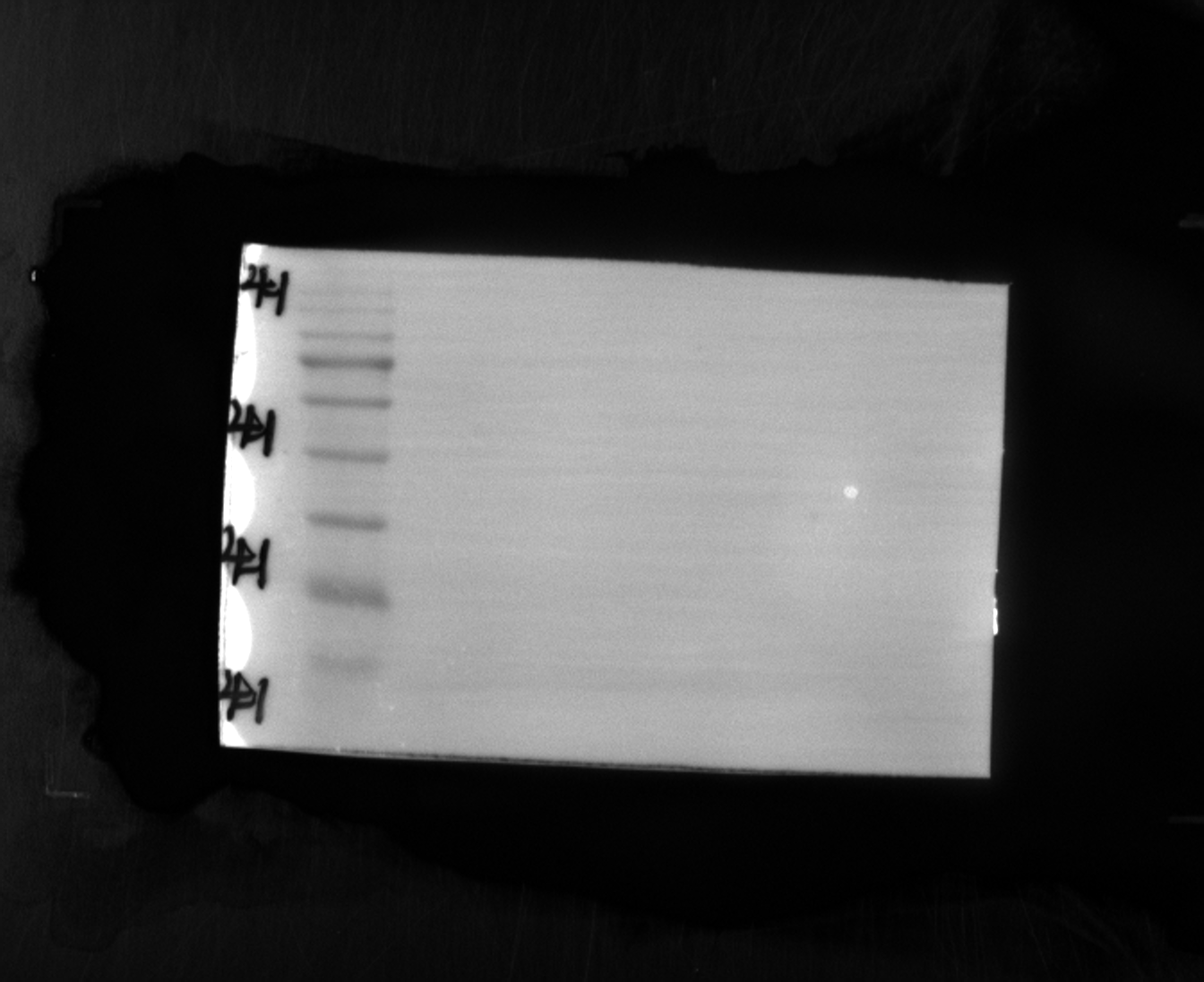

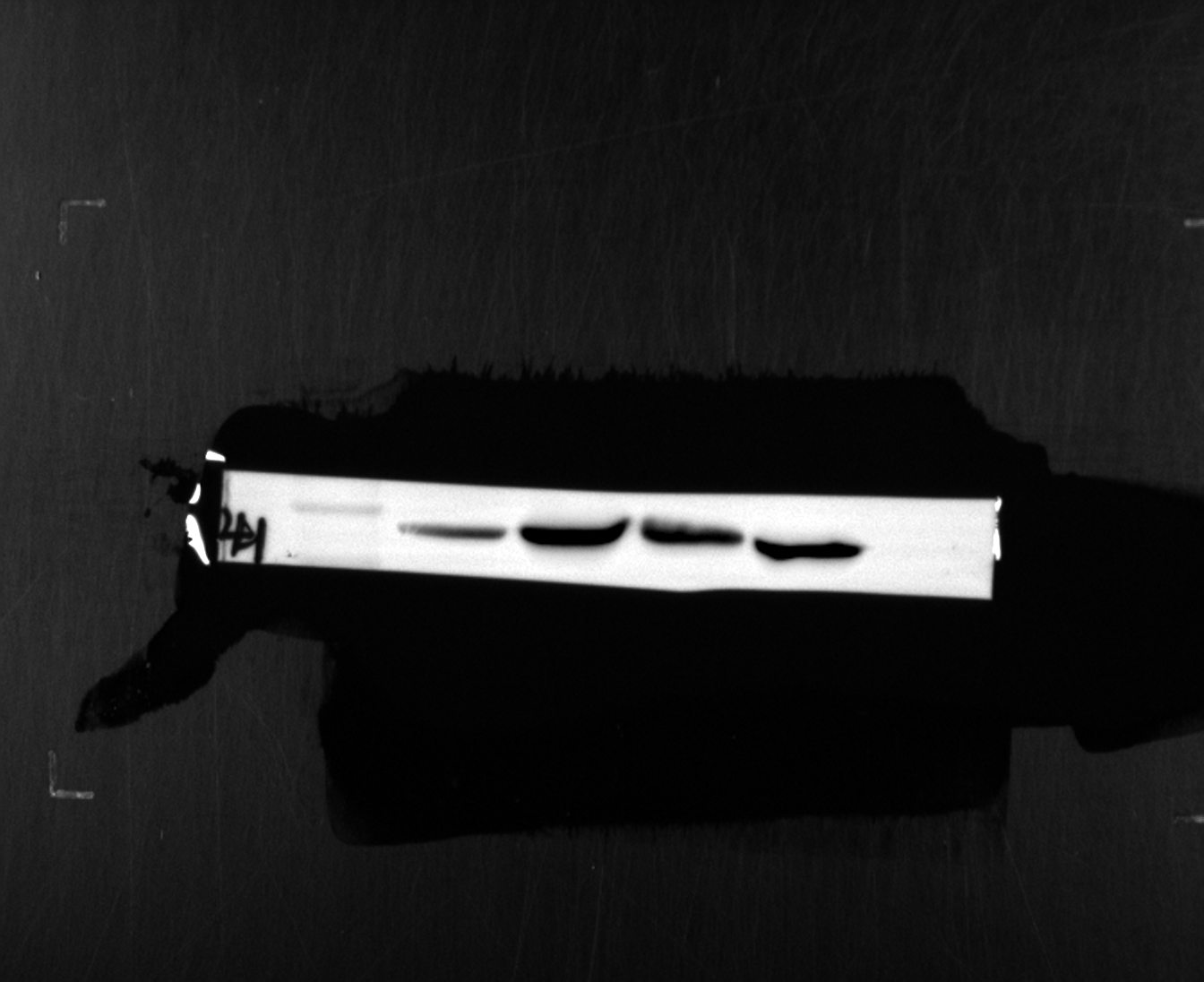


Figure 3D GAPDH 1


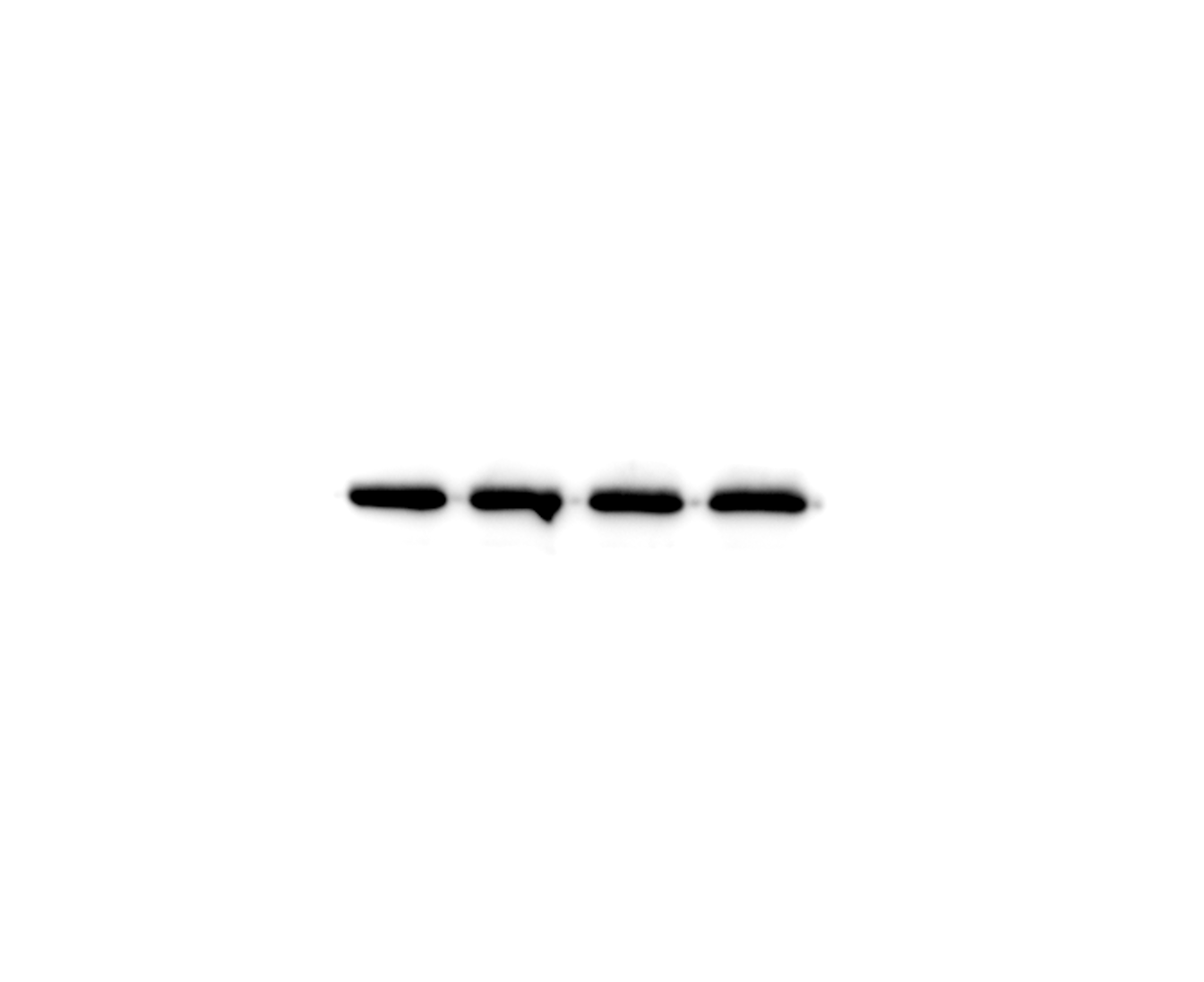

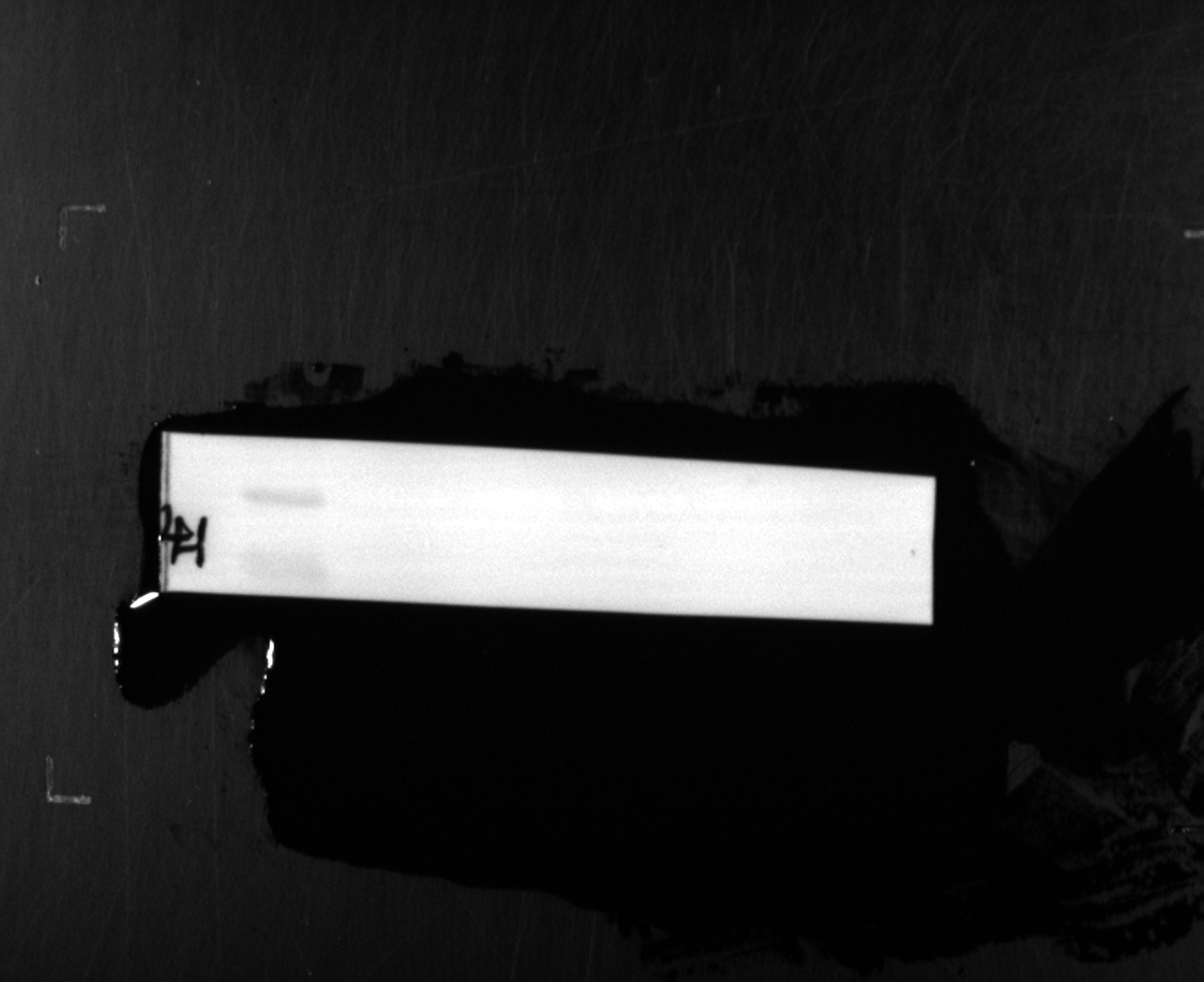

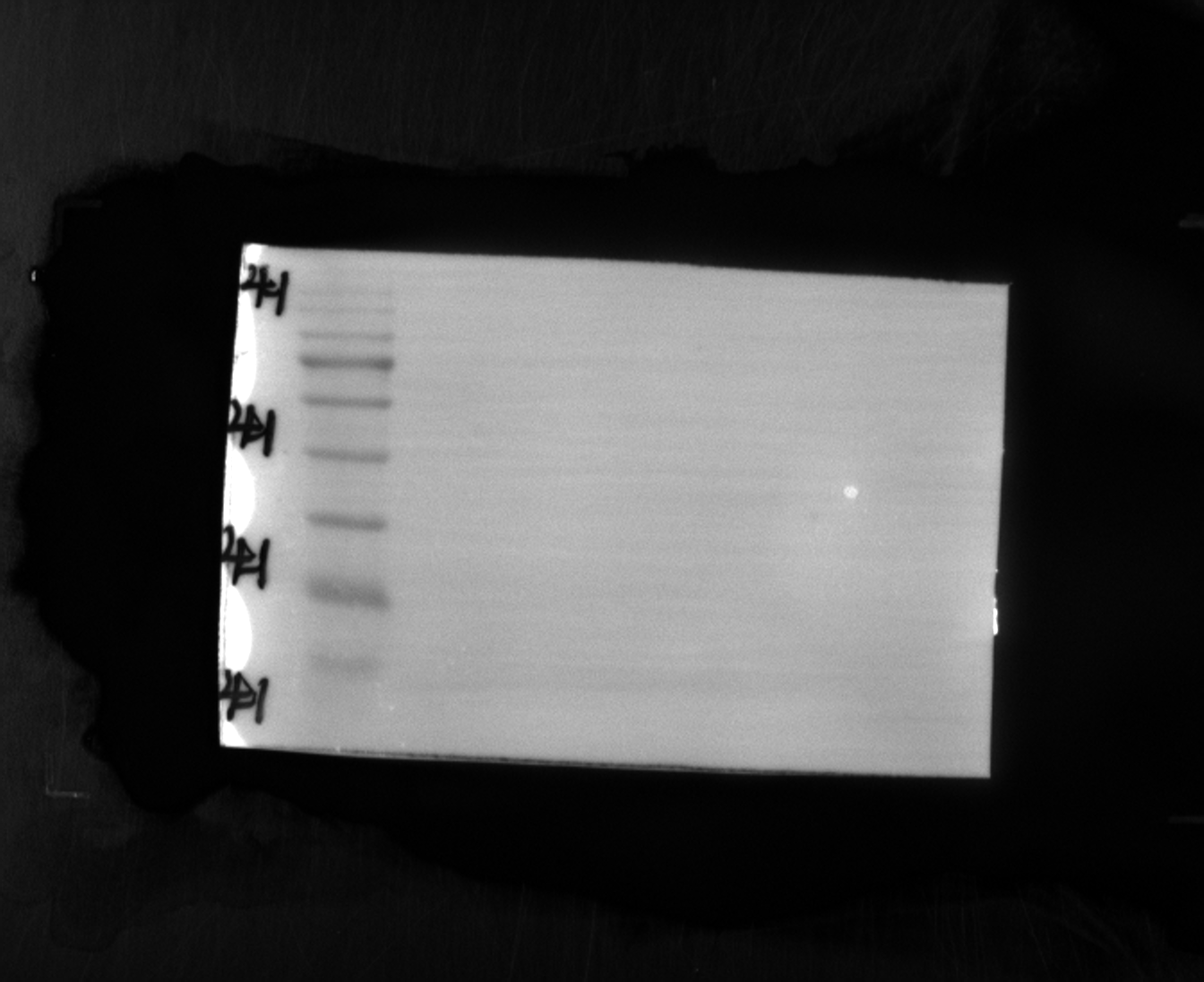

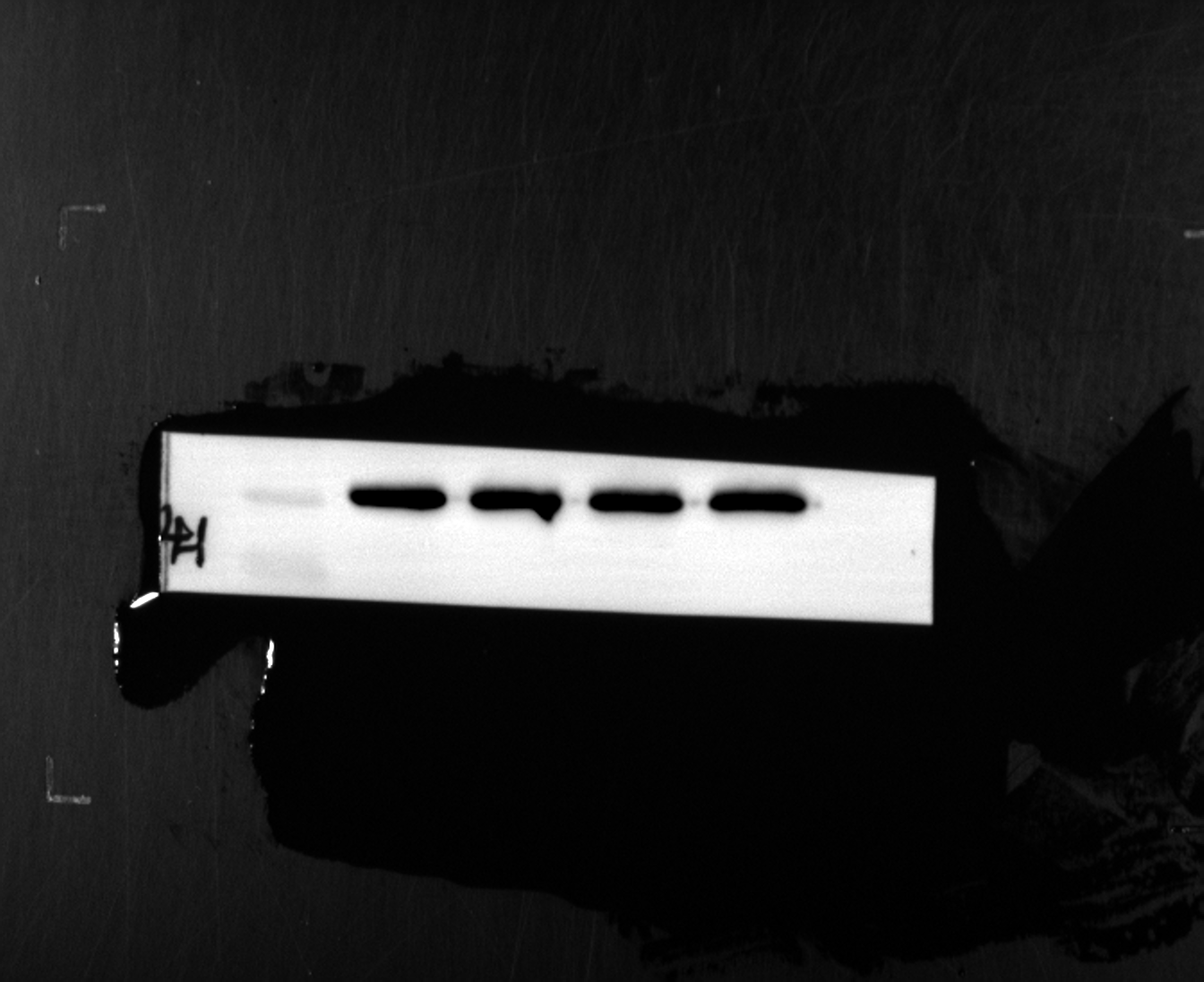


Figure 3D GAPDH 2


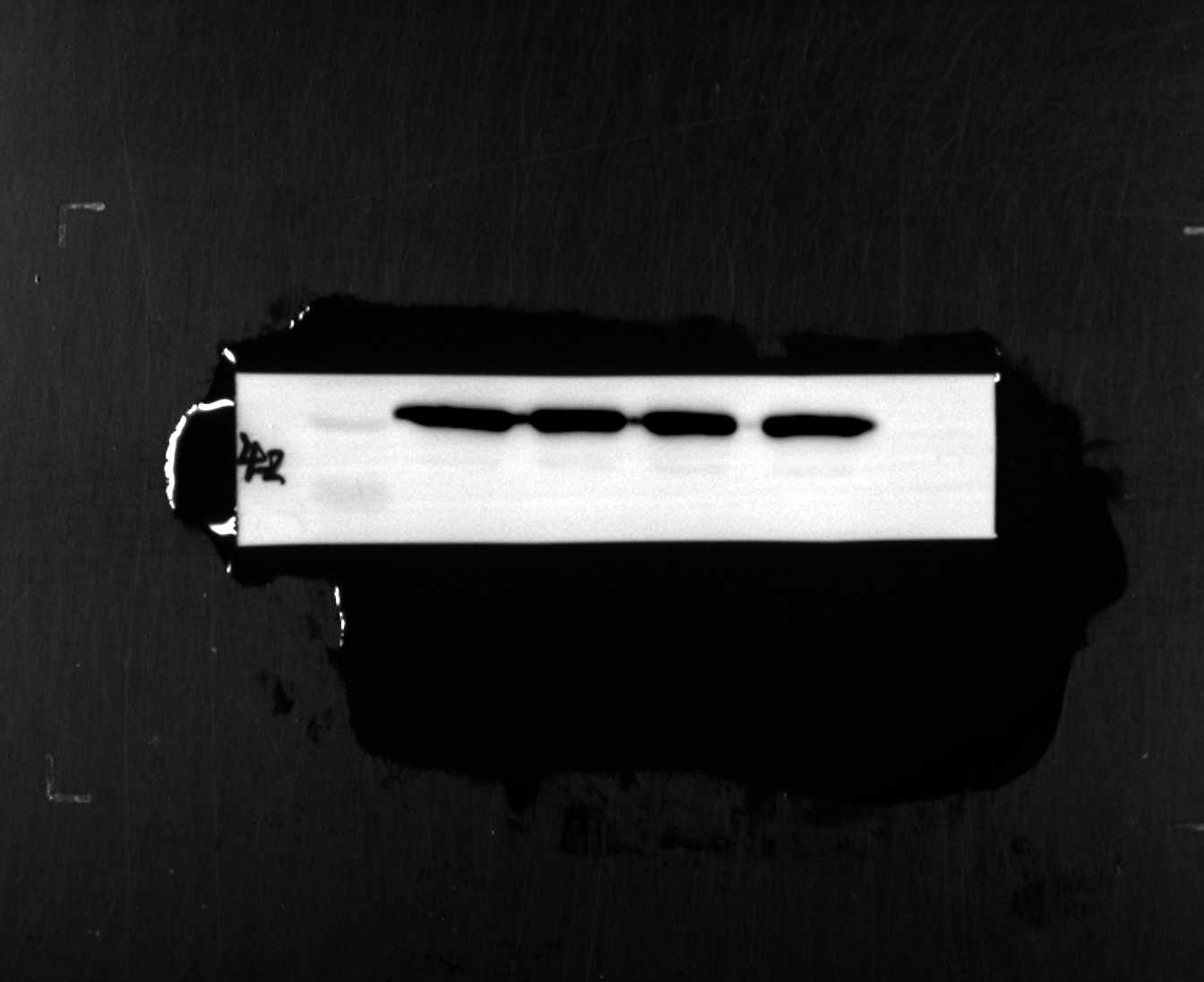

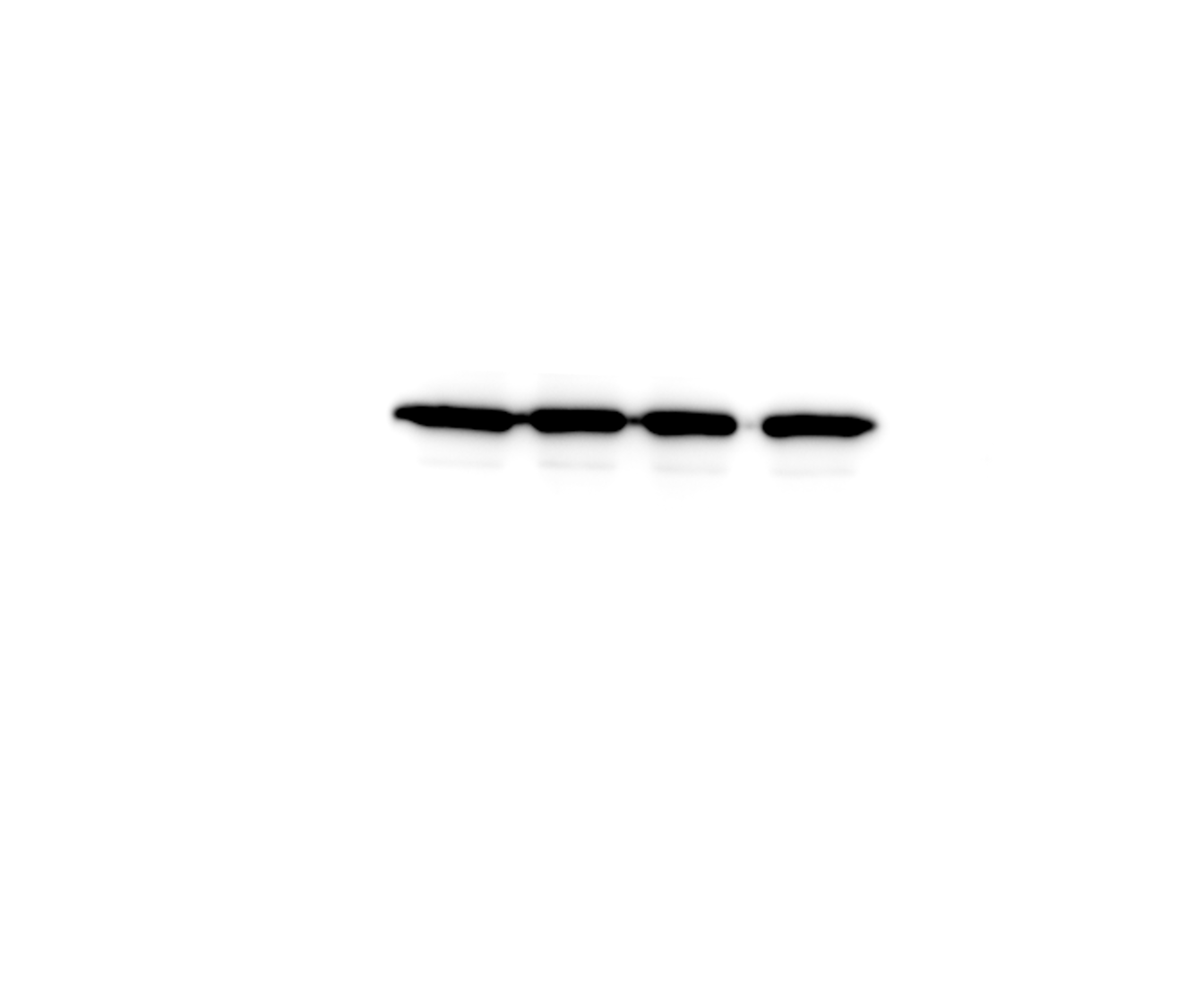

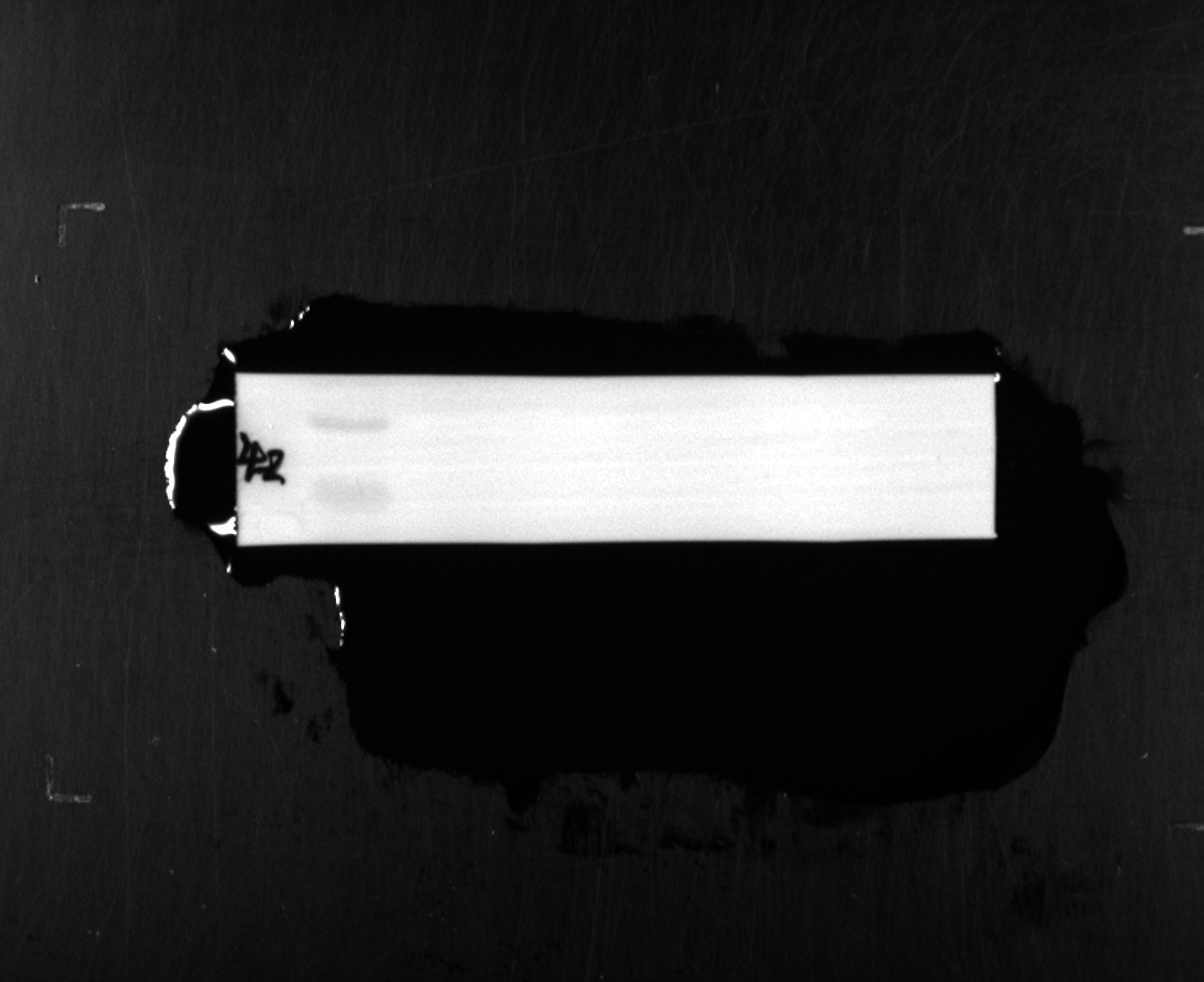

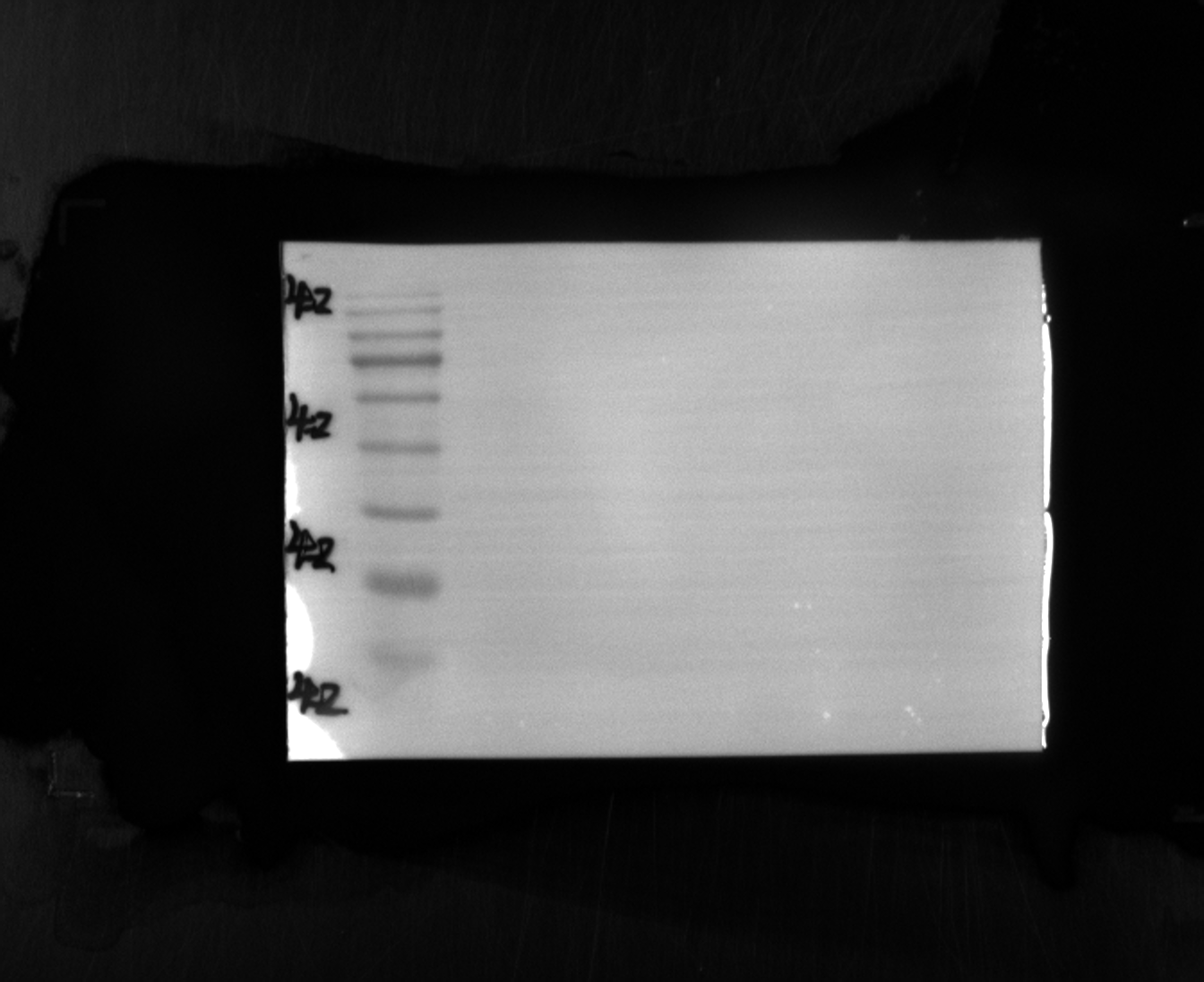


Figure 3D Capase-1


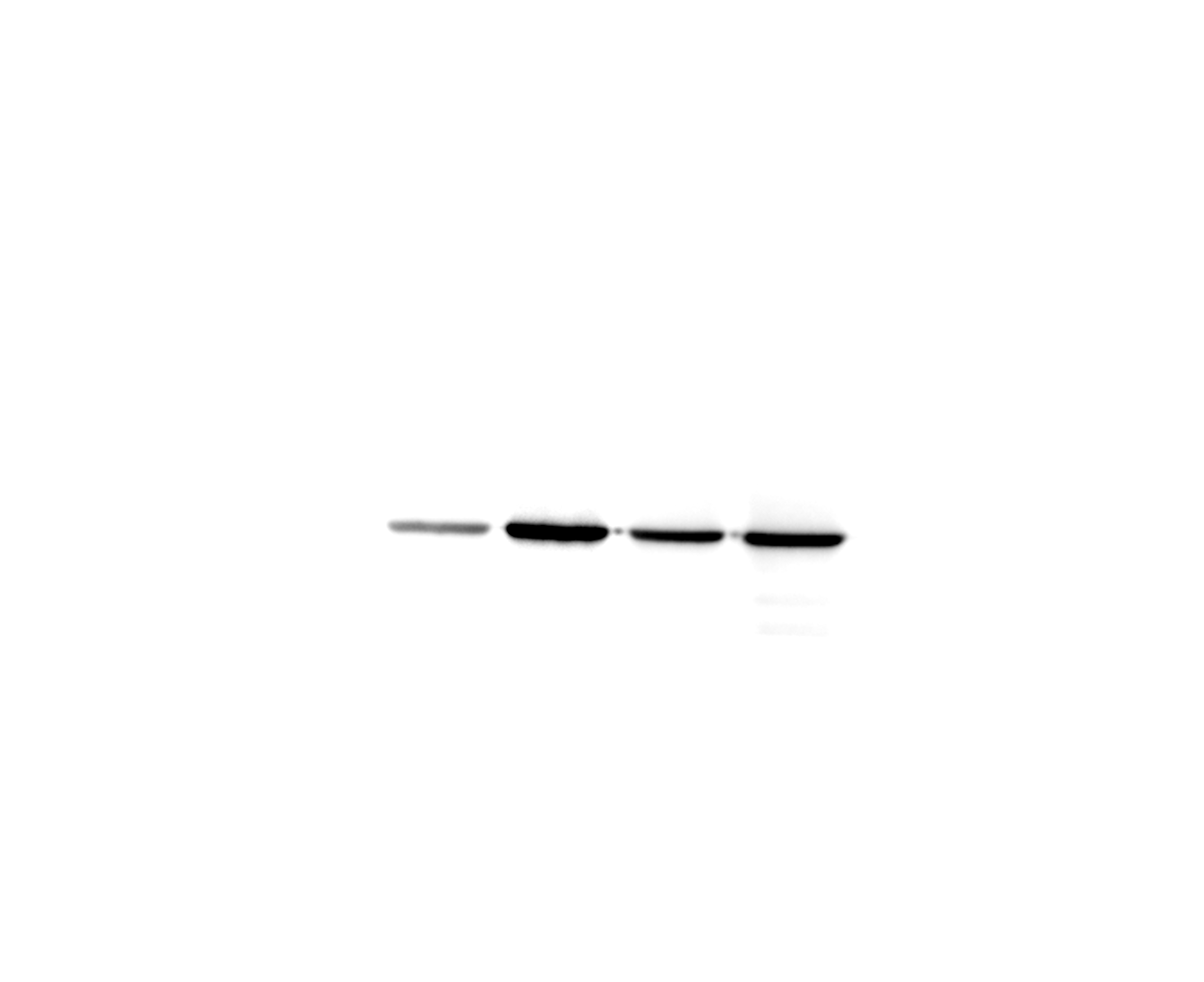

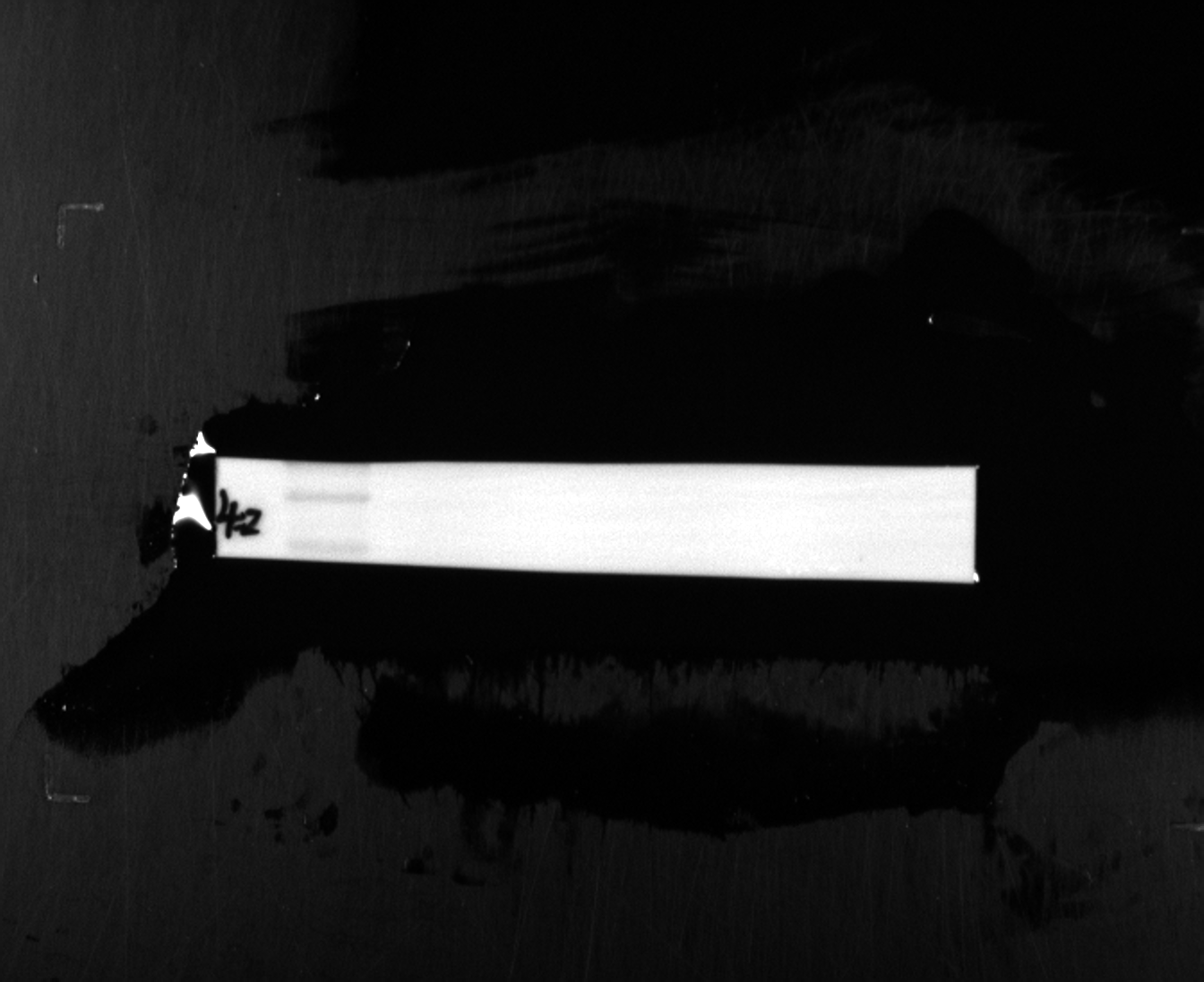

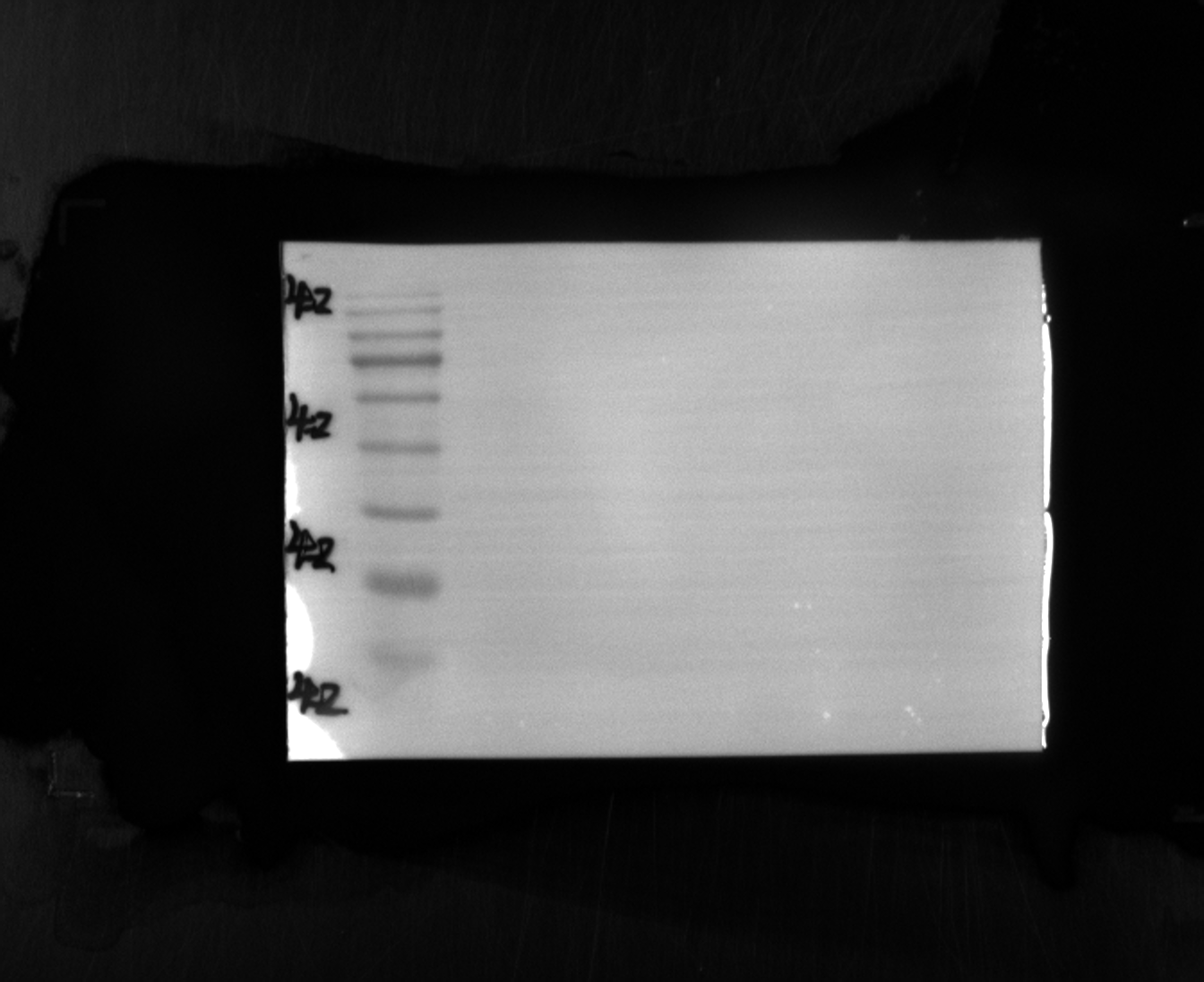

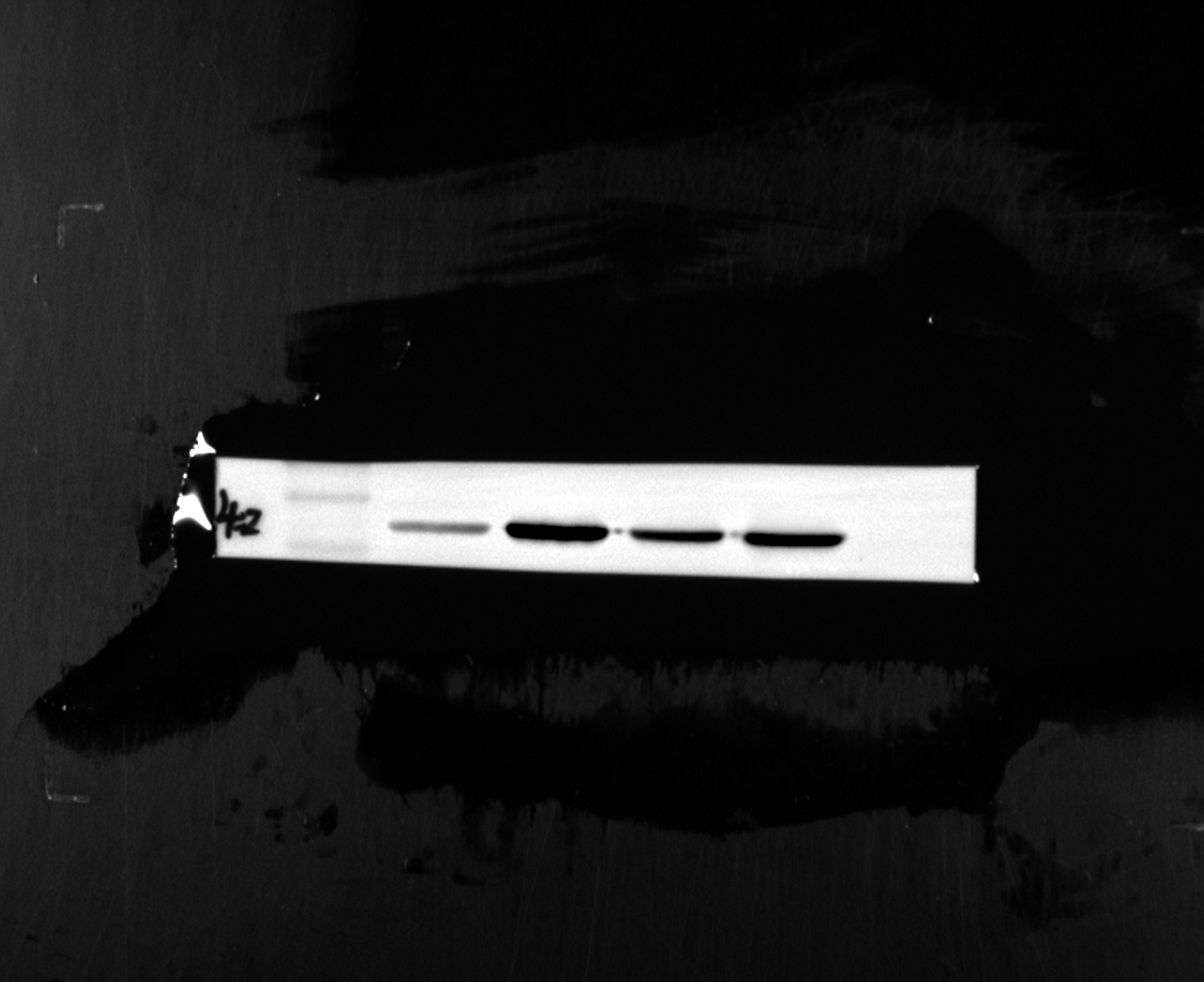


Figure 4D STAT3


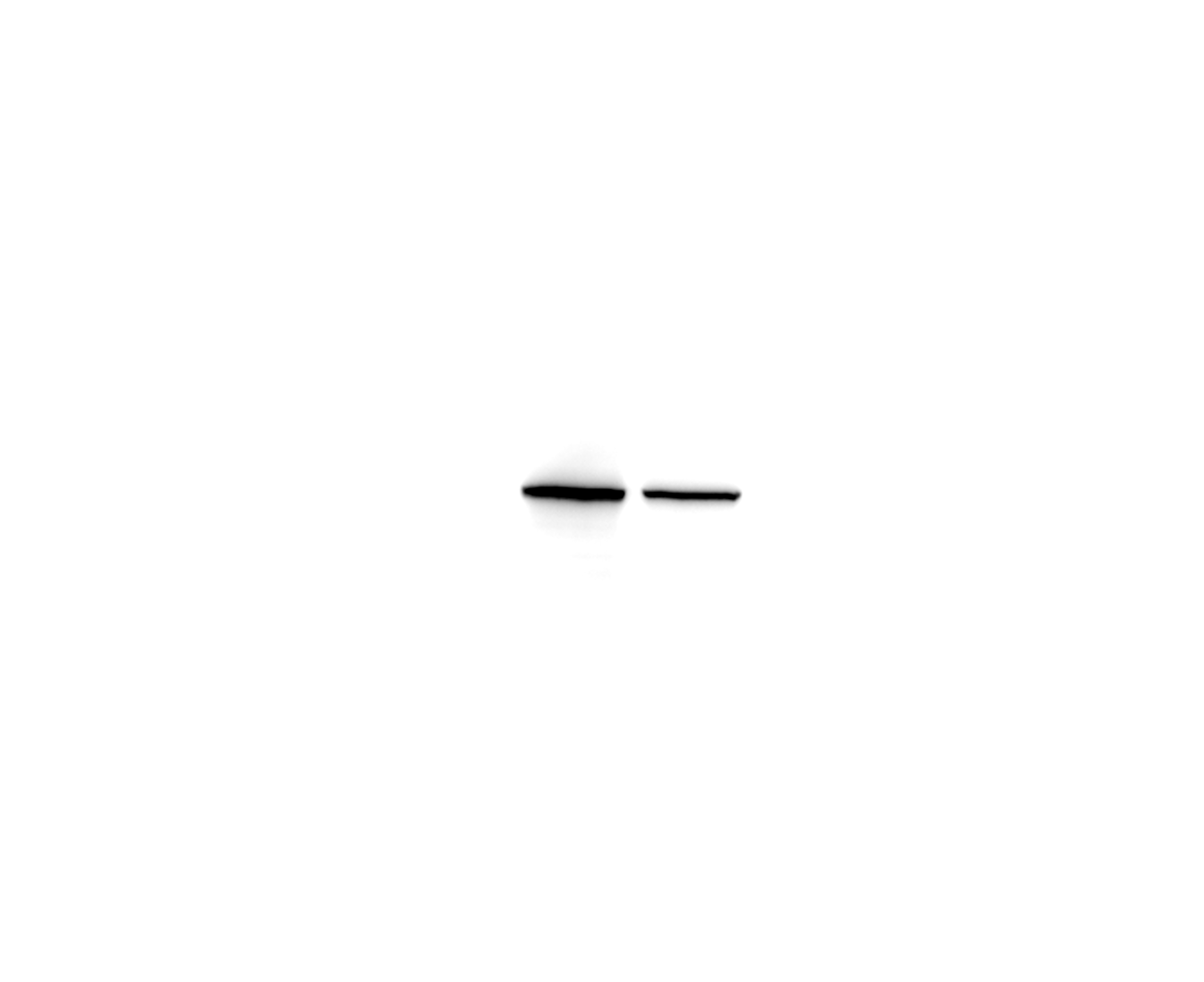

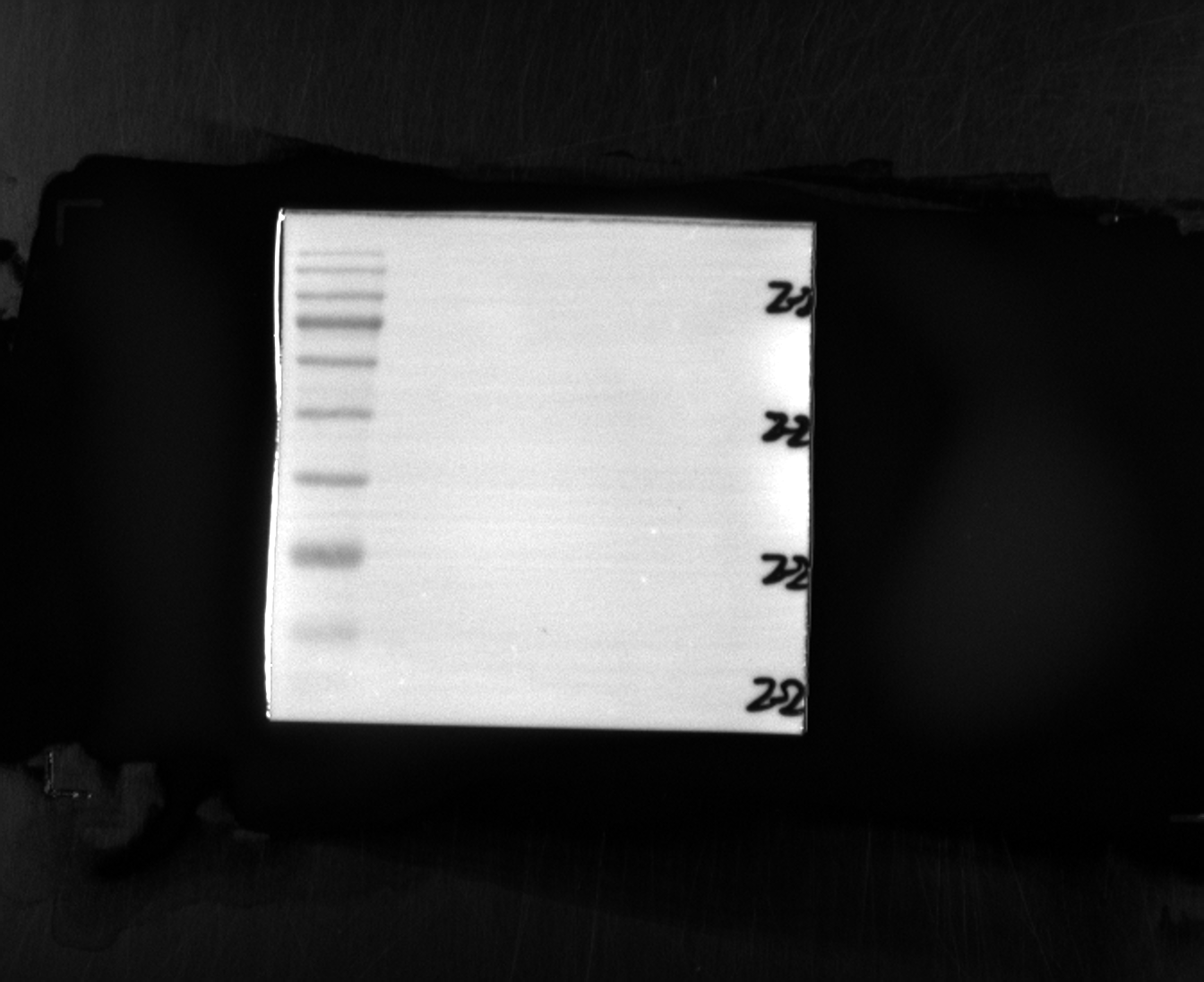

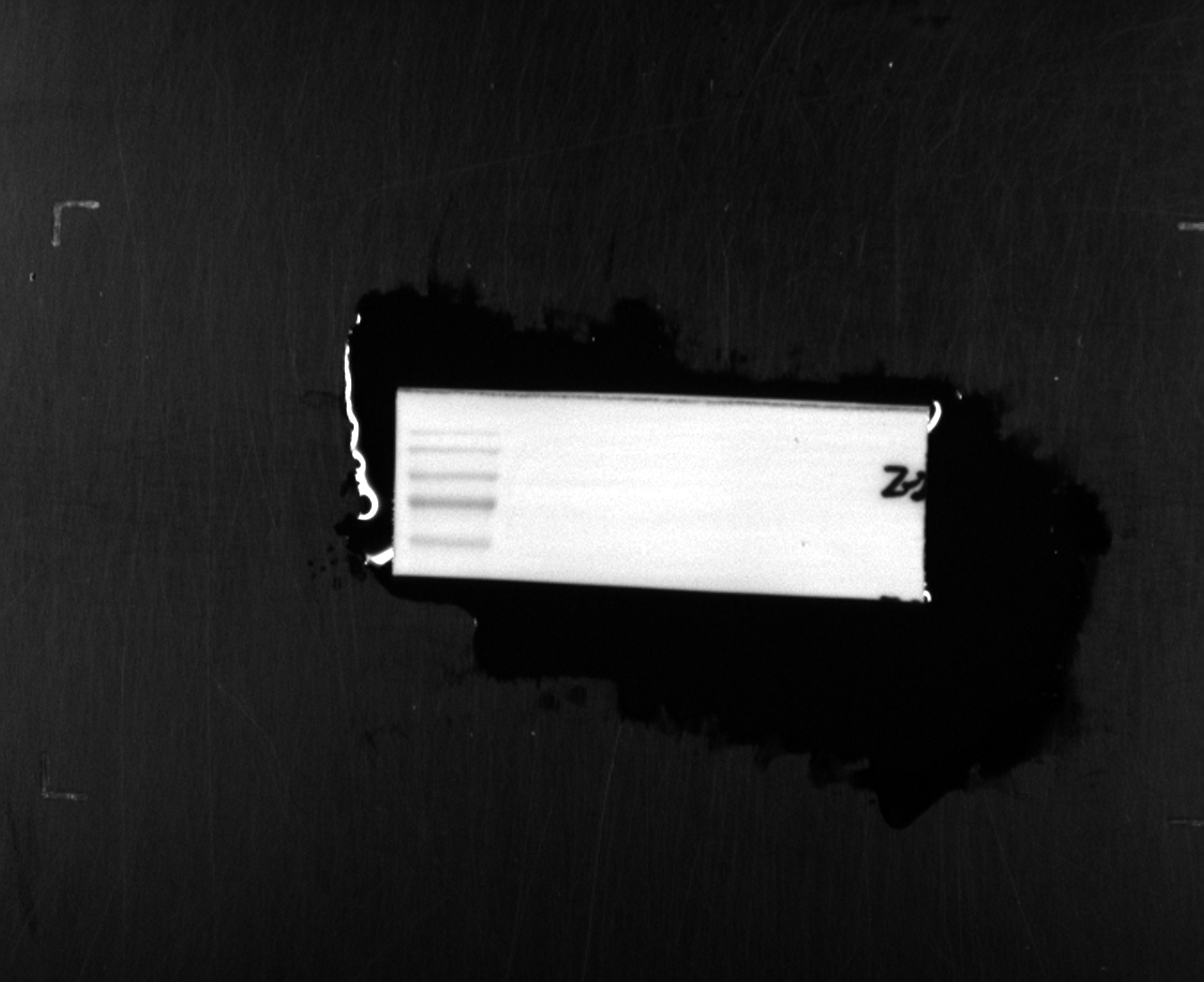

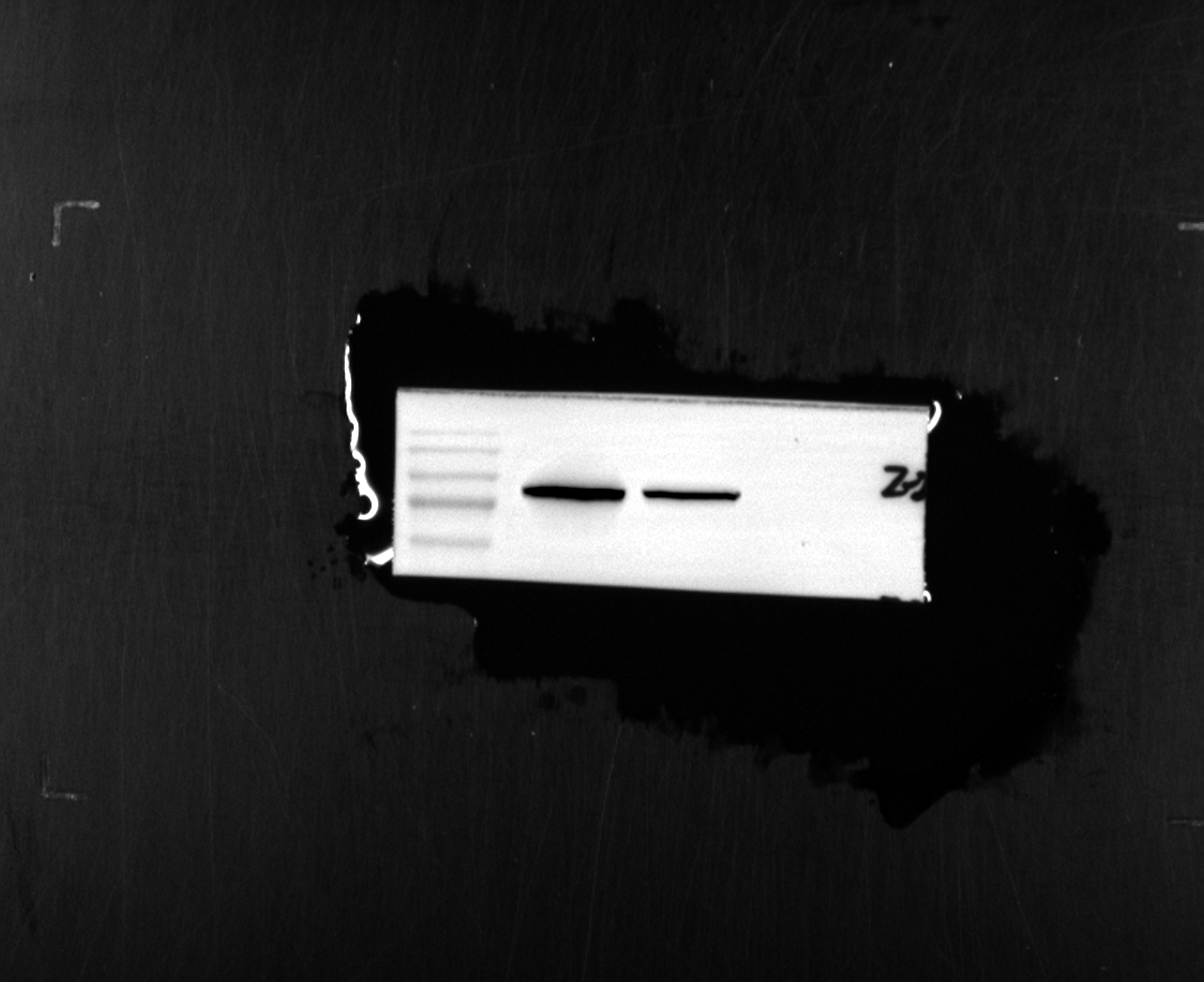


Figure 4D GAPDH


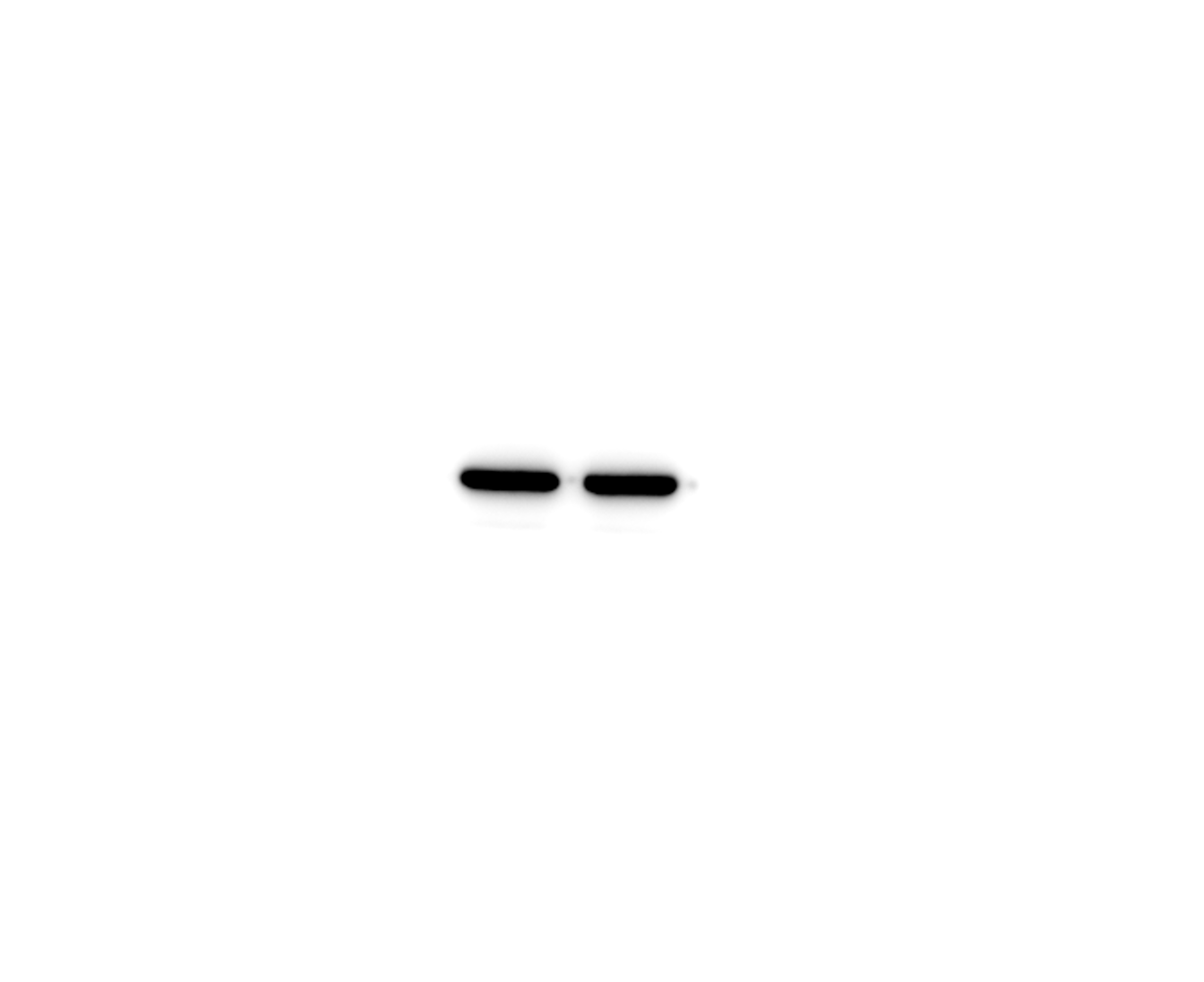

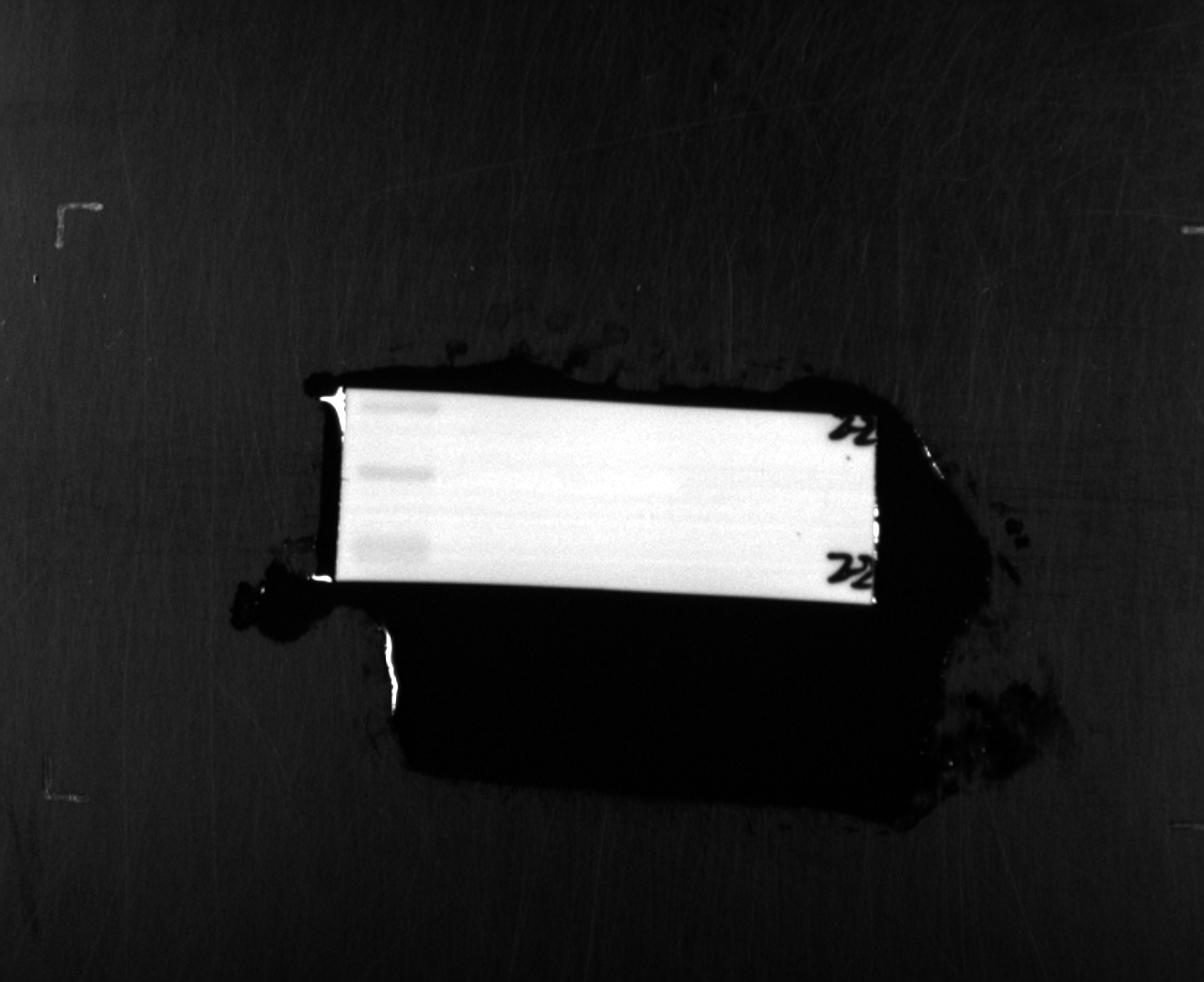

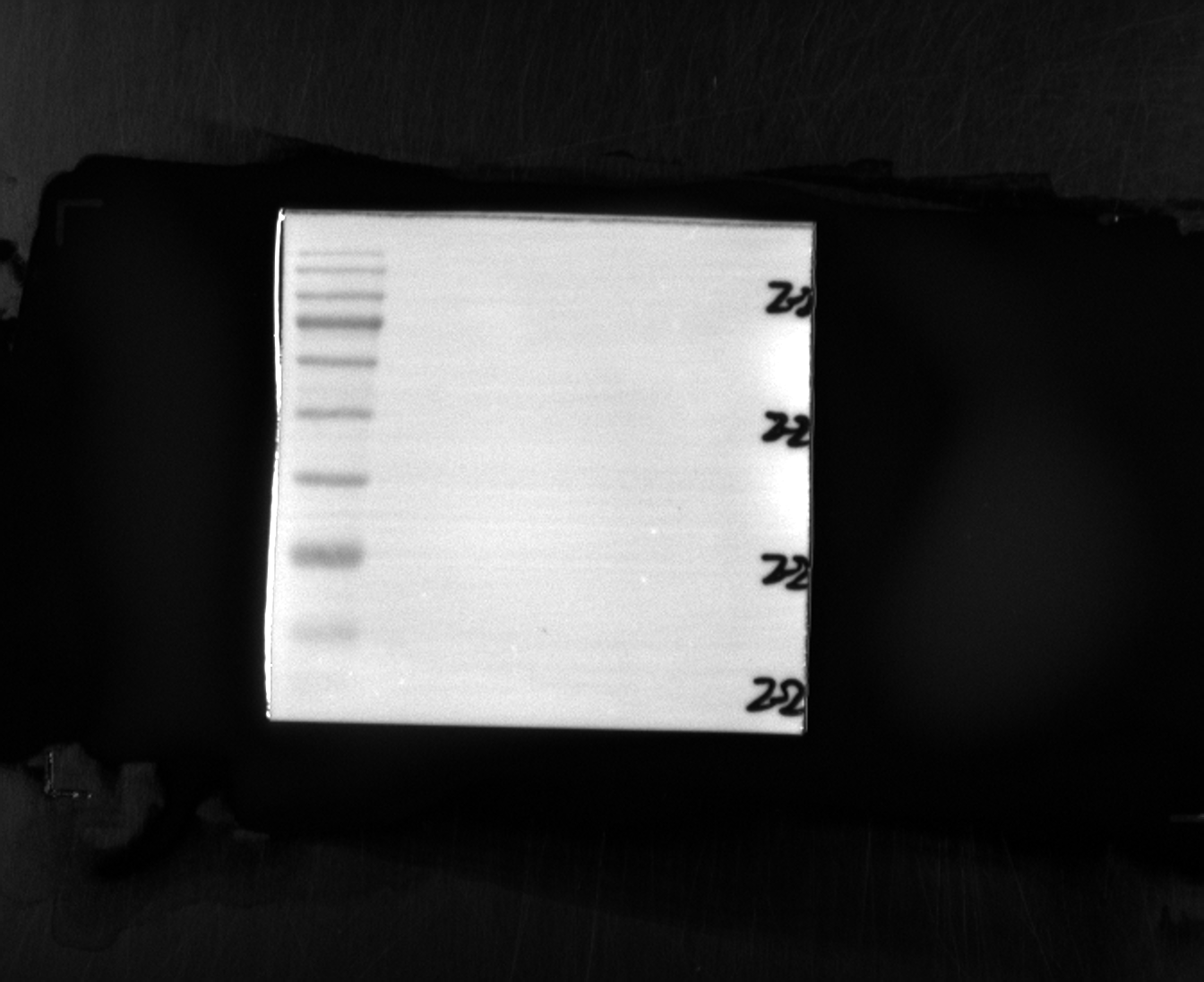

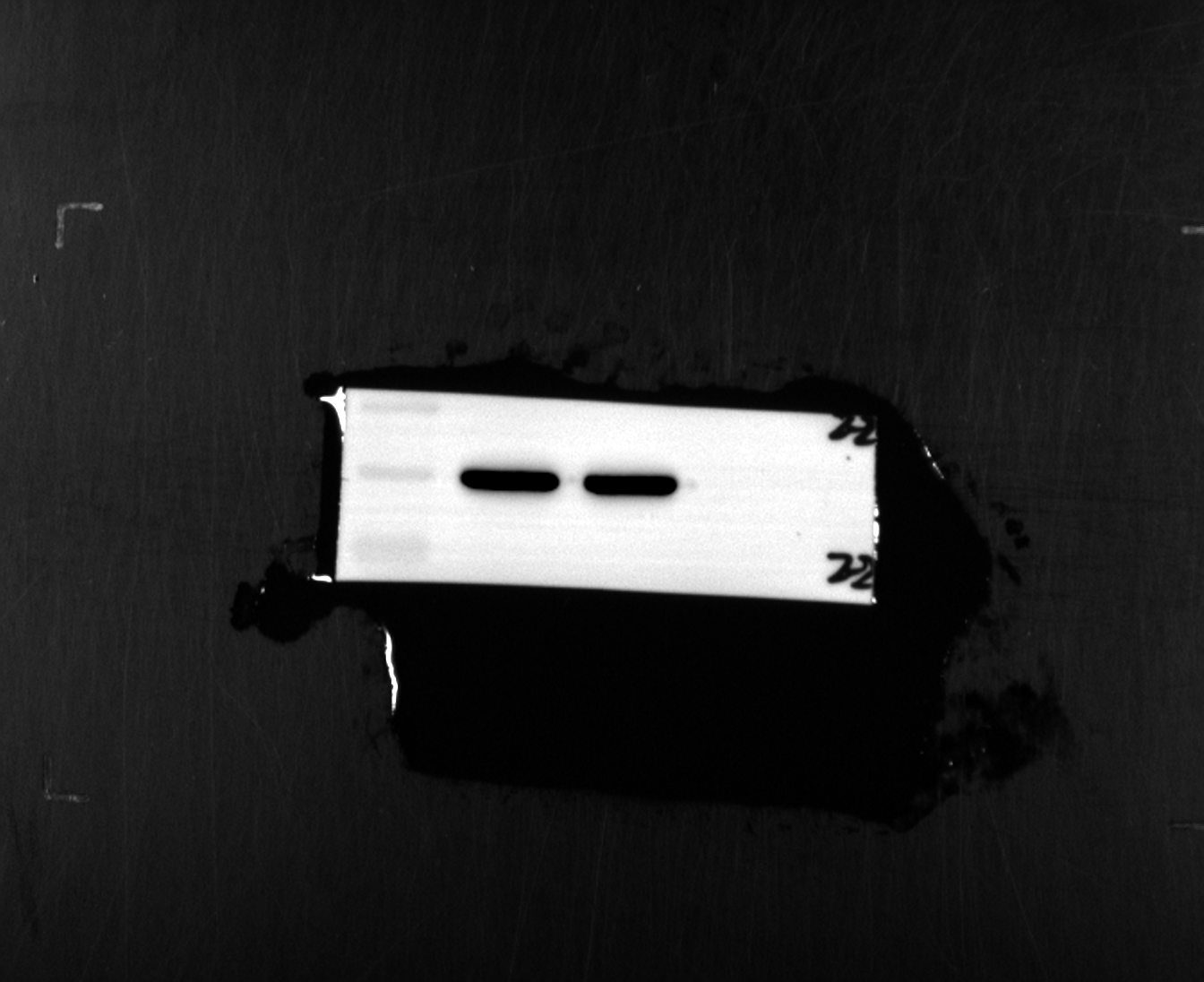


Figure 5A STAT3


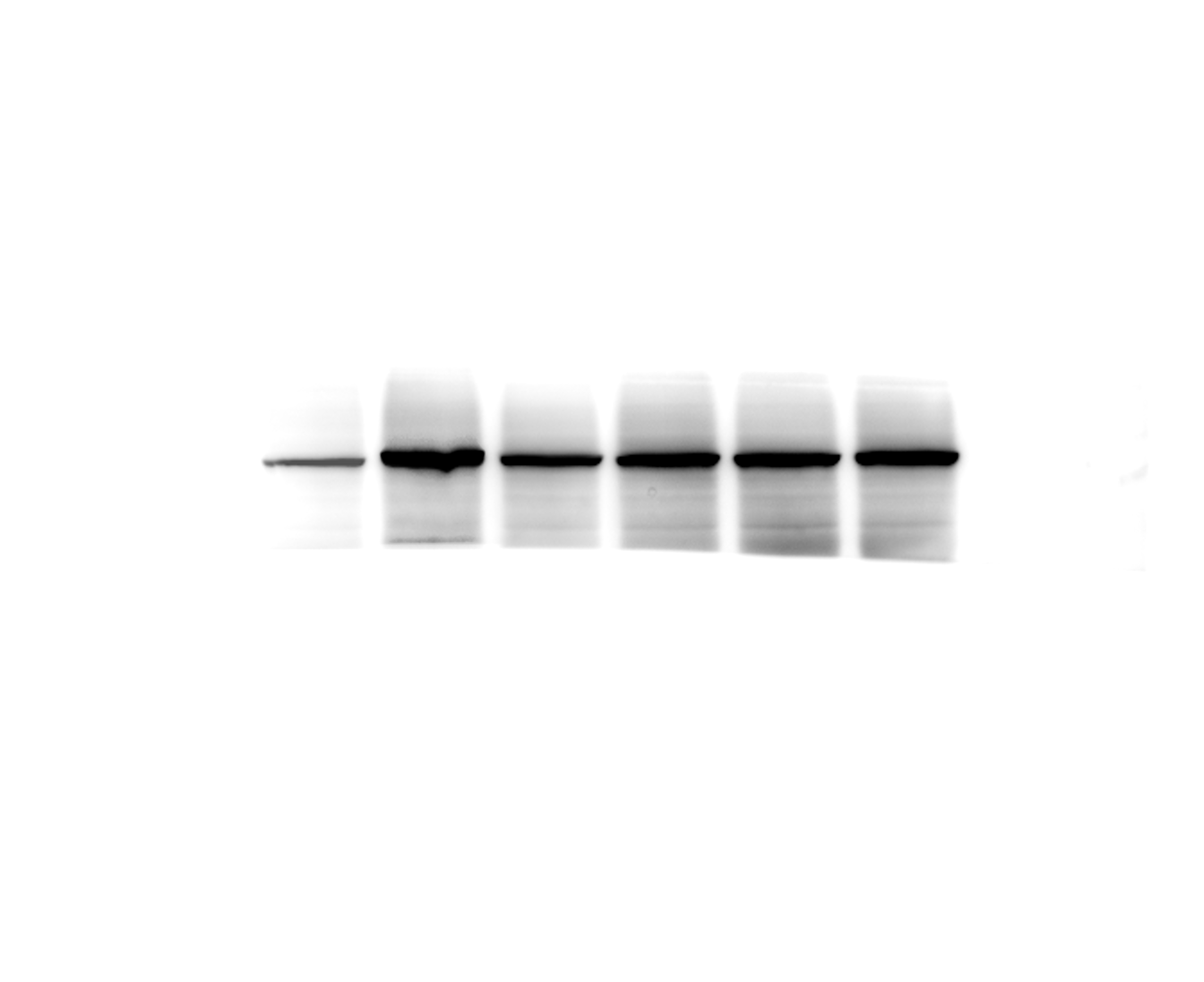

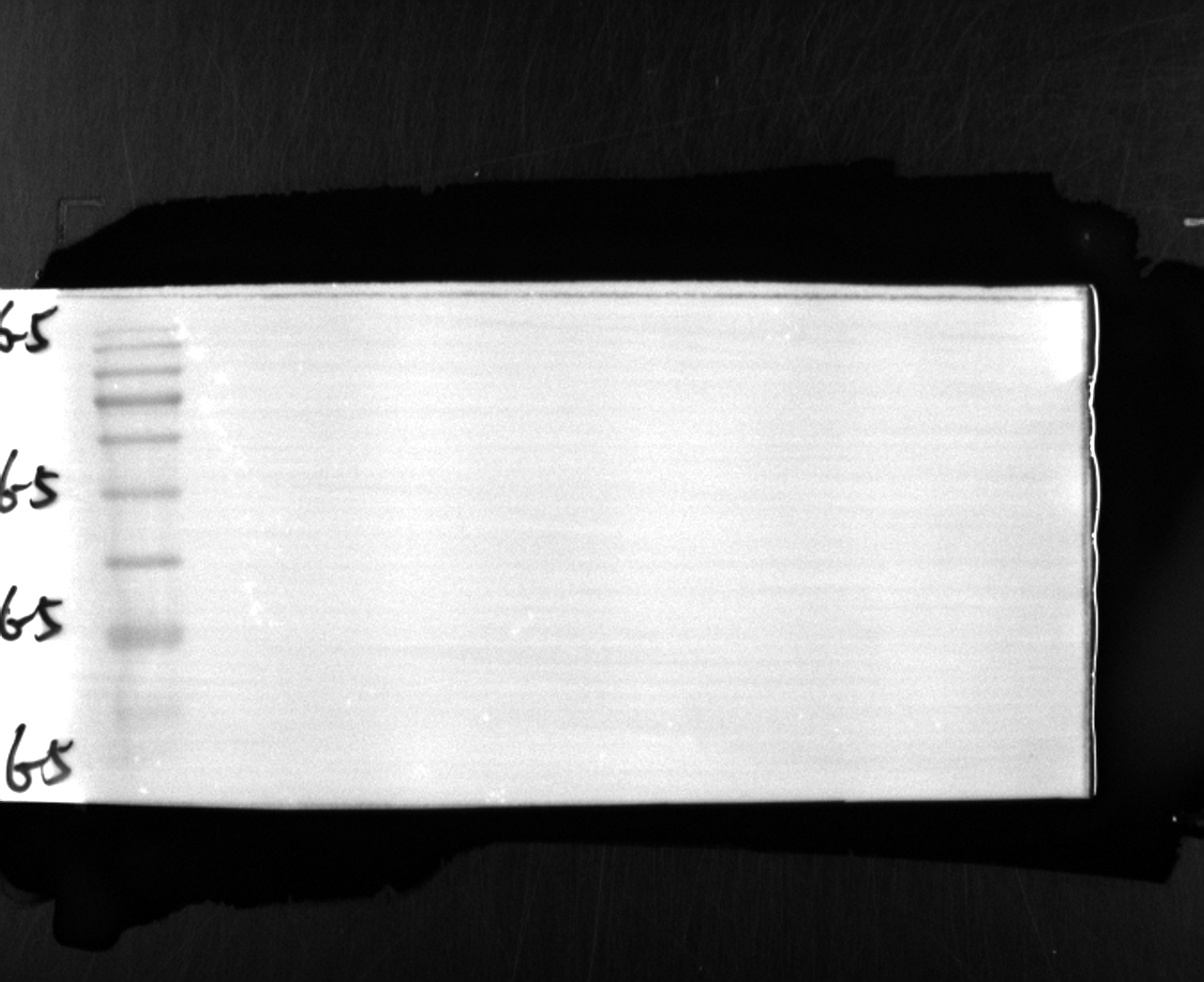

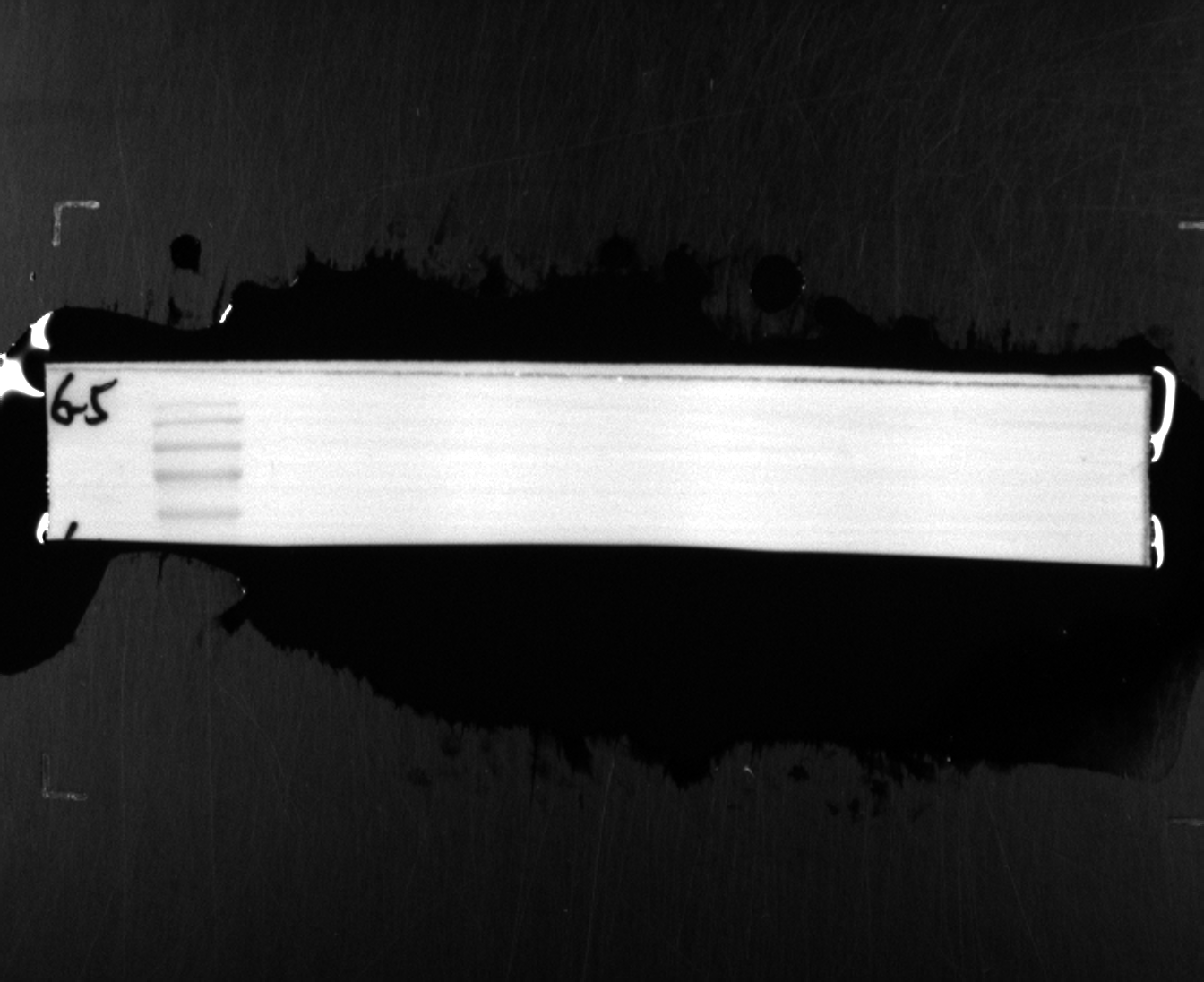

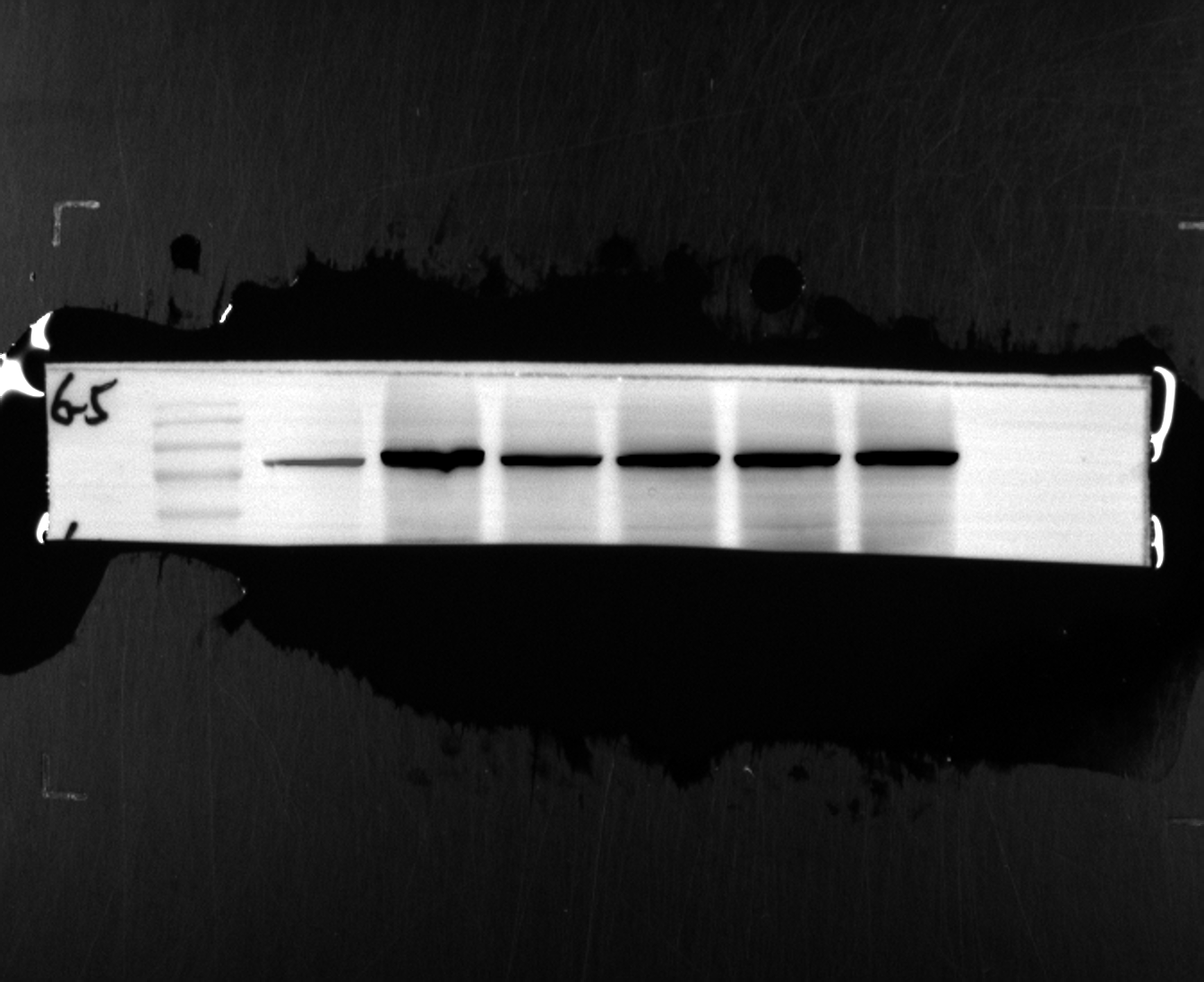


Figure 5A GAPDH


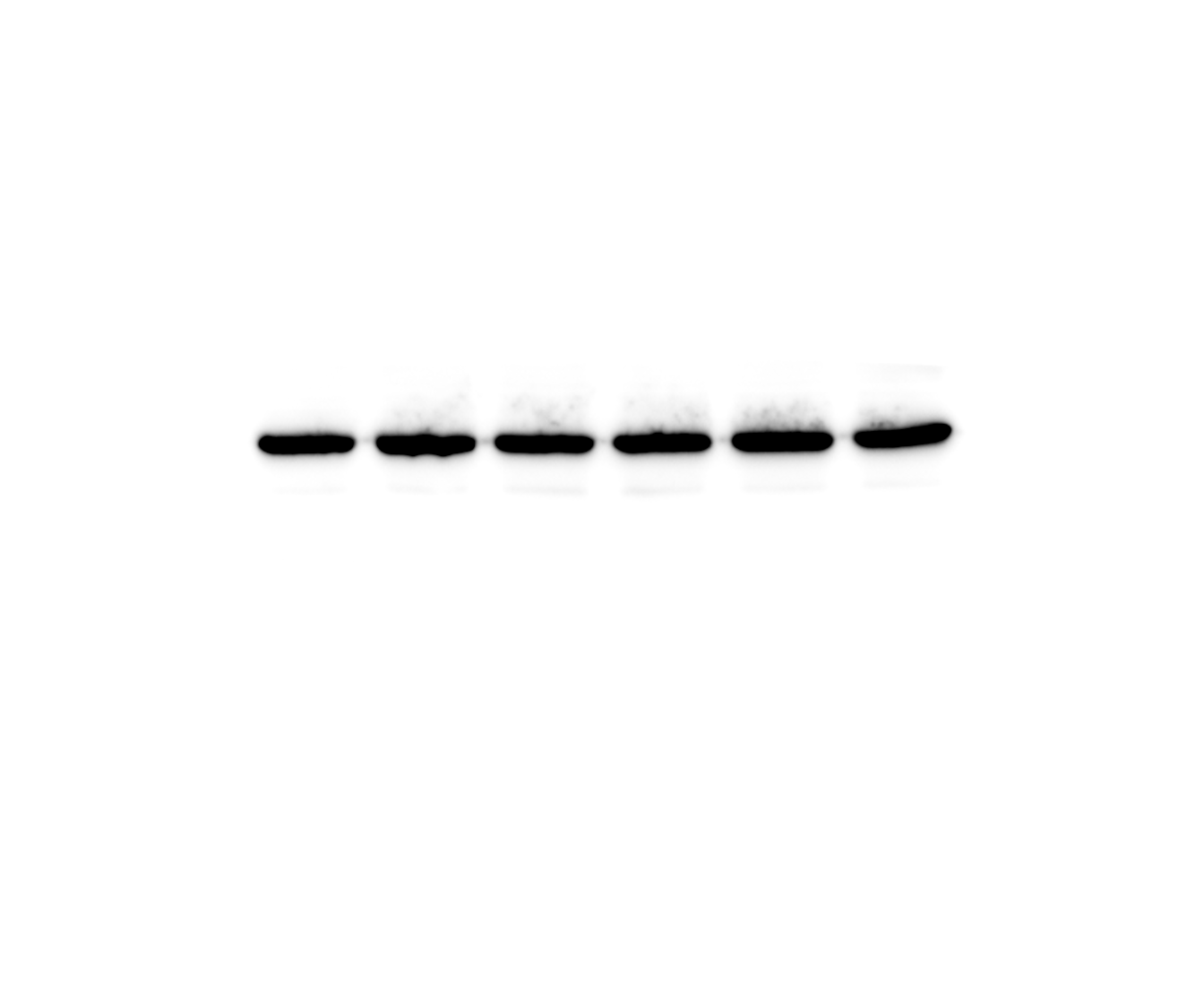

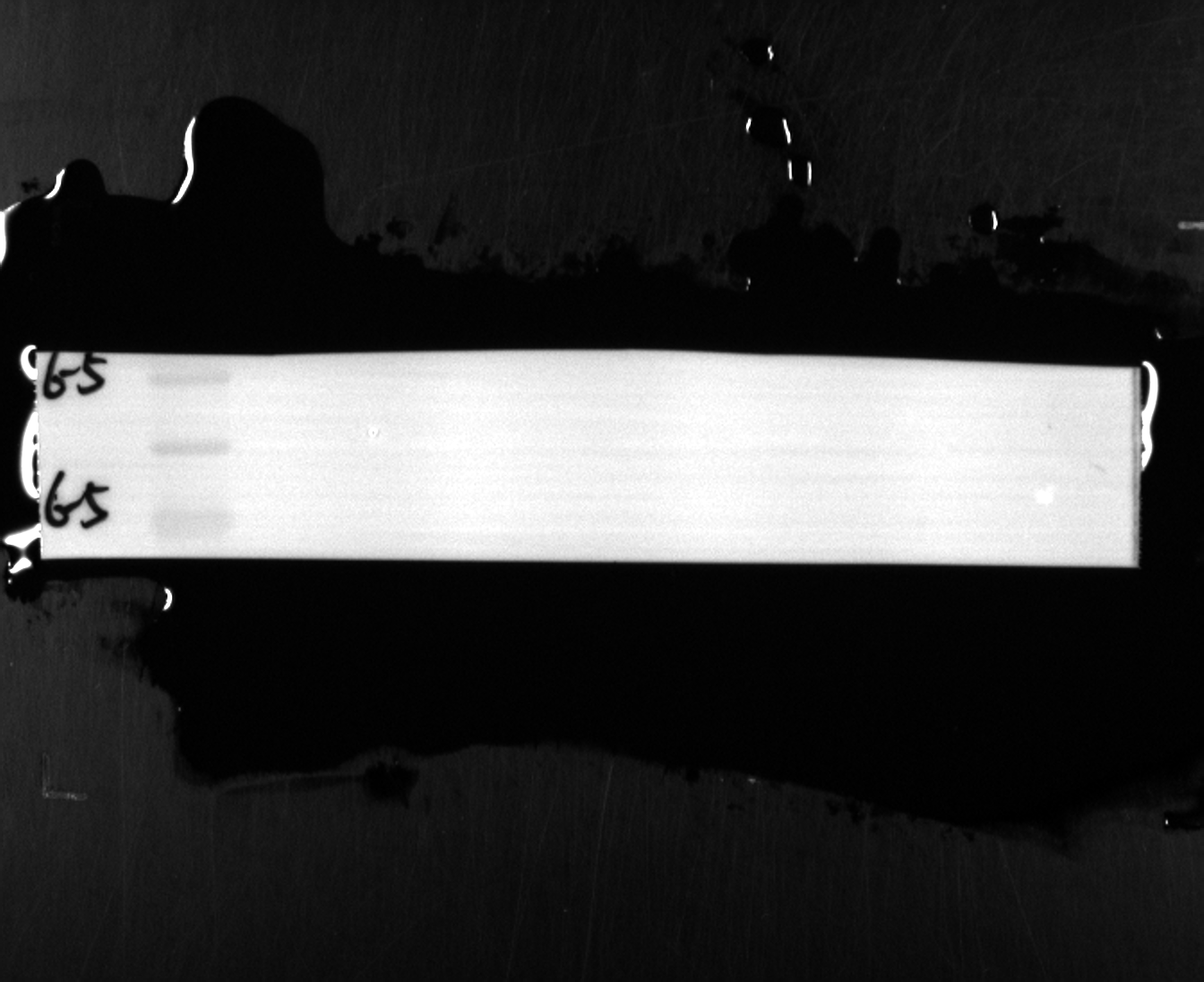

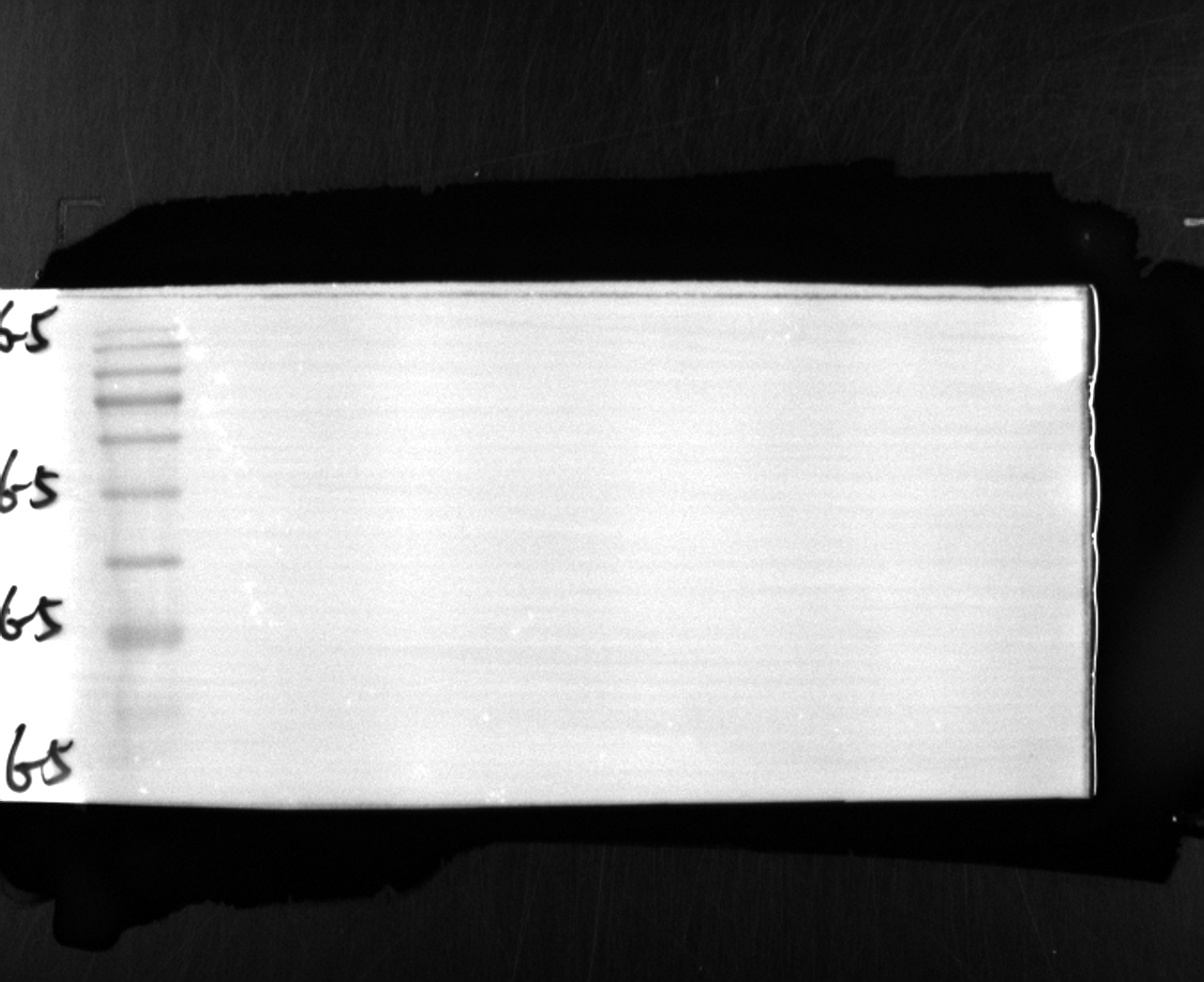

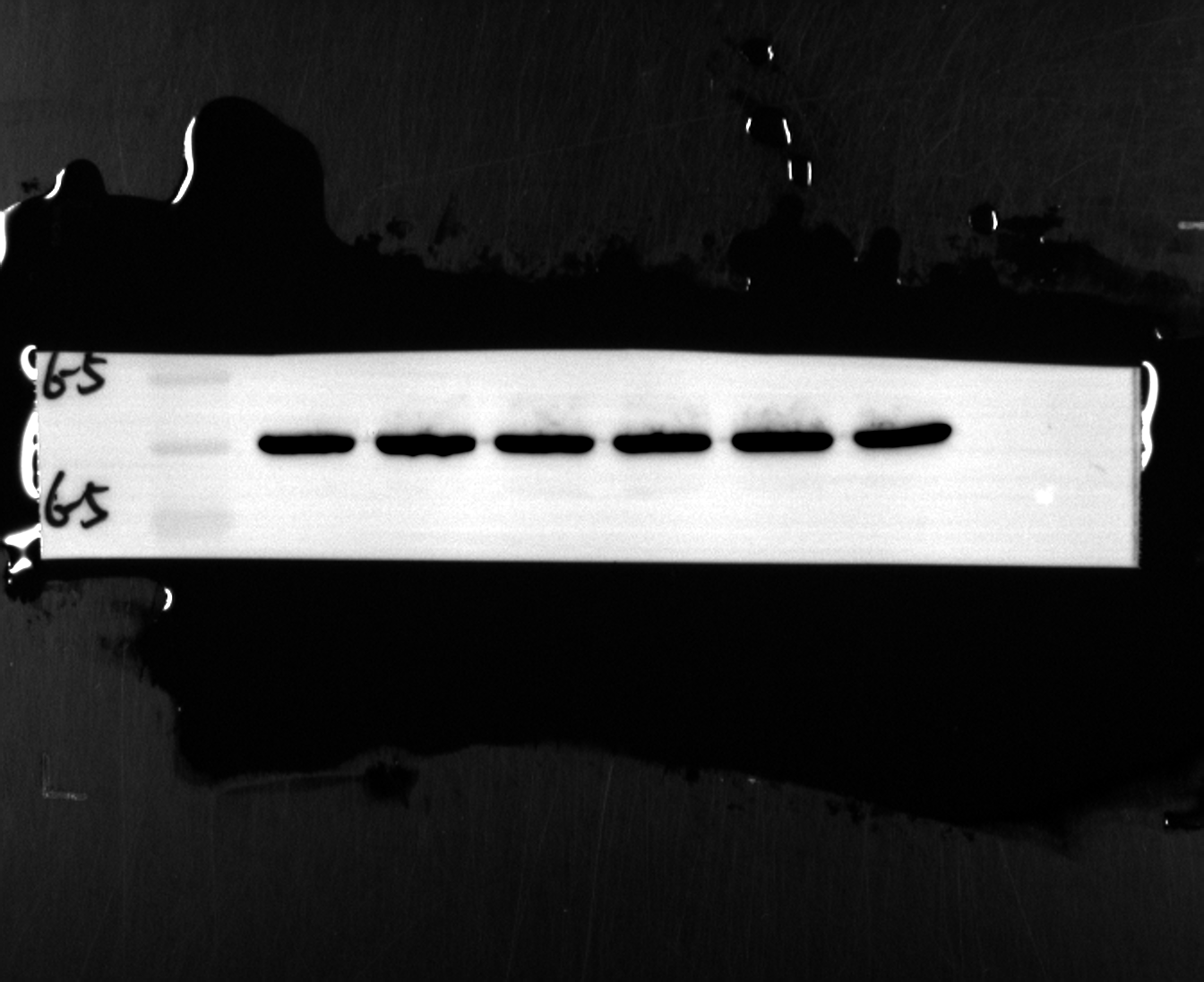


Figure 5D GSDMD


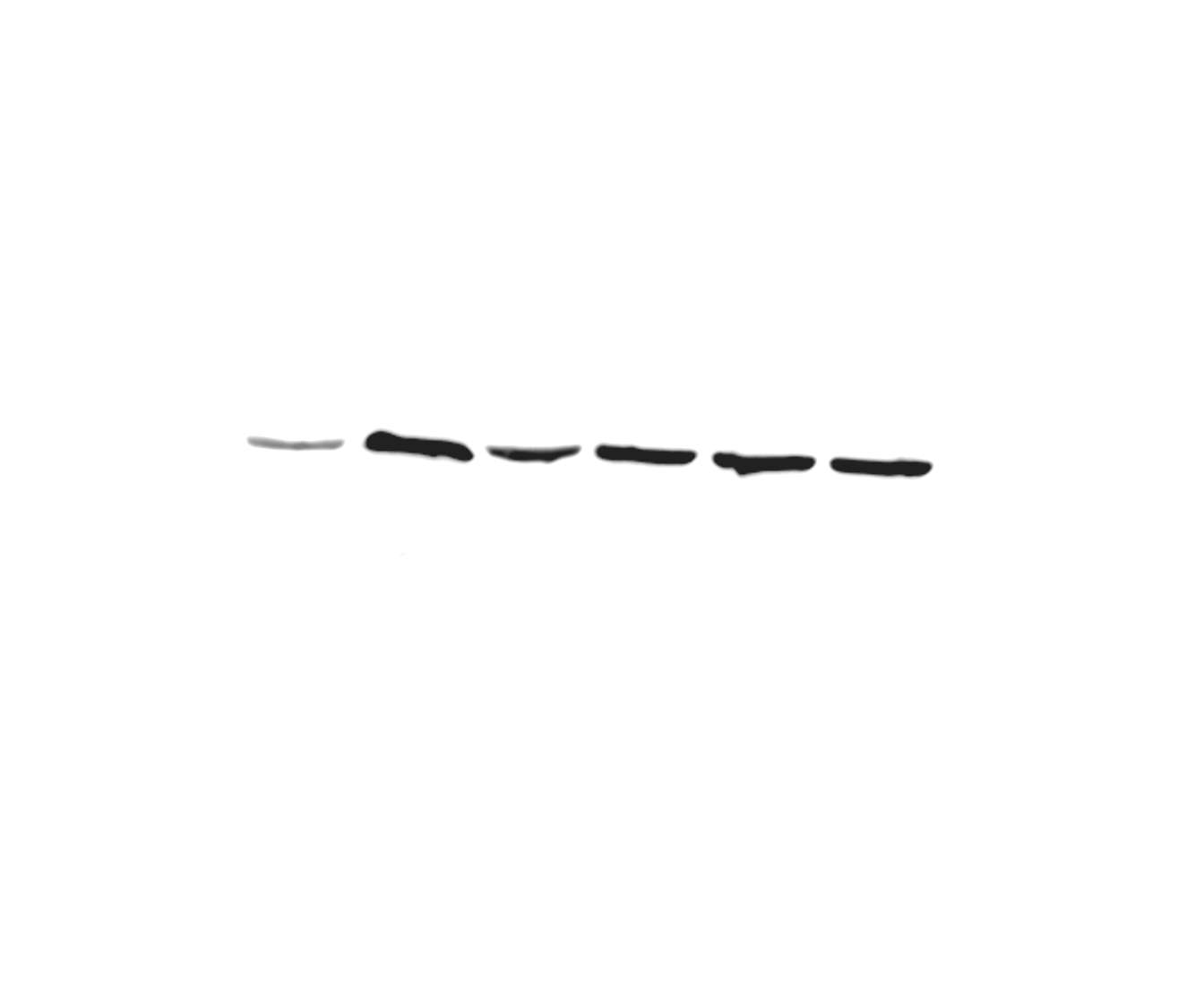

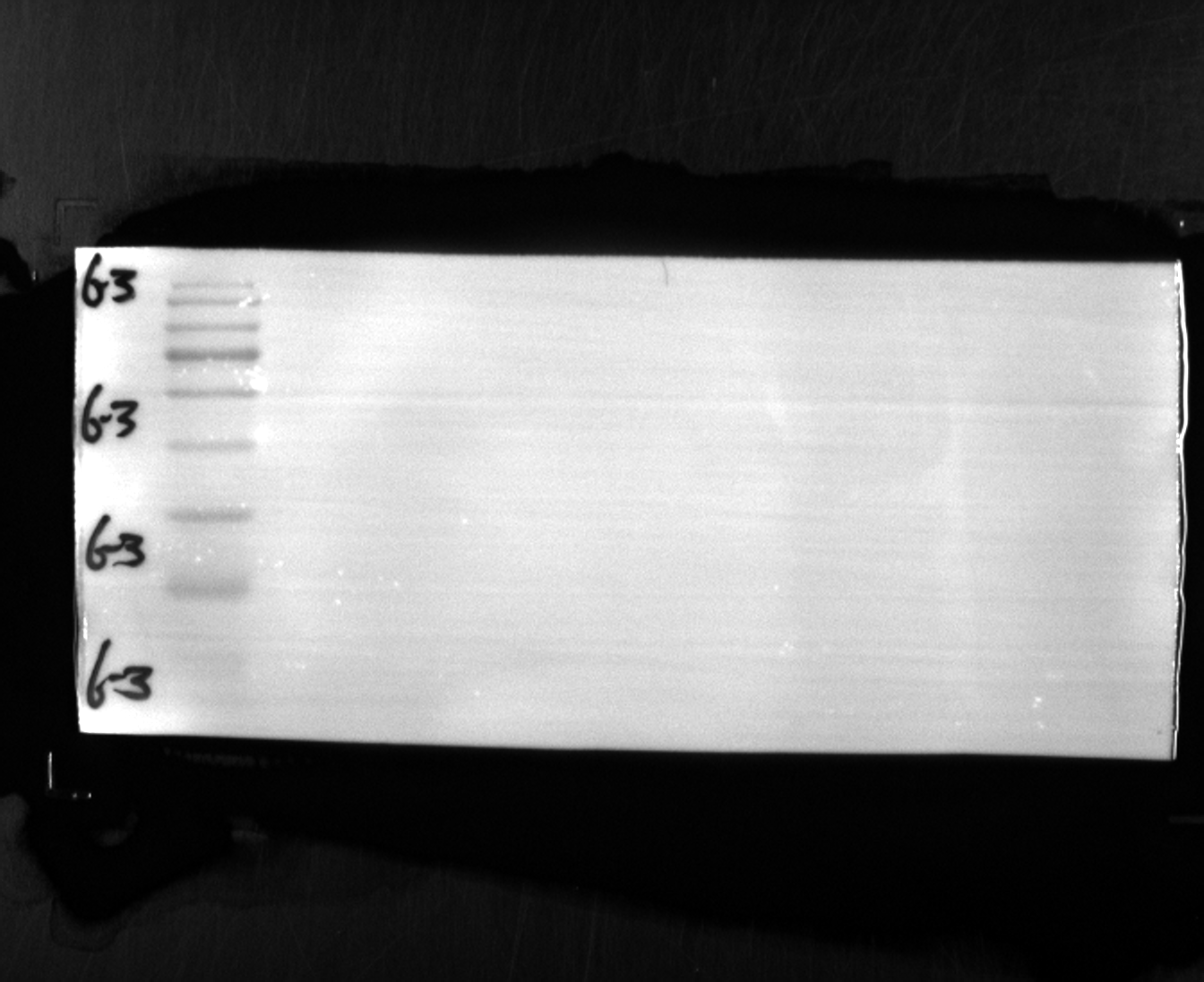

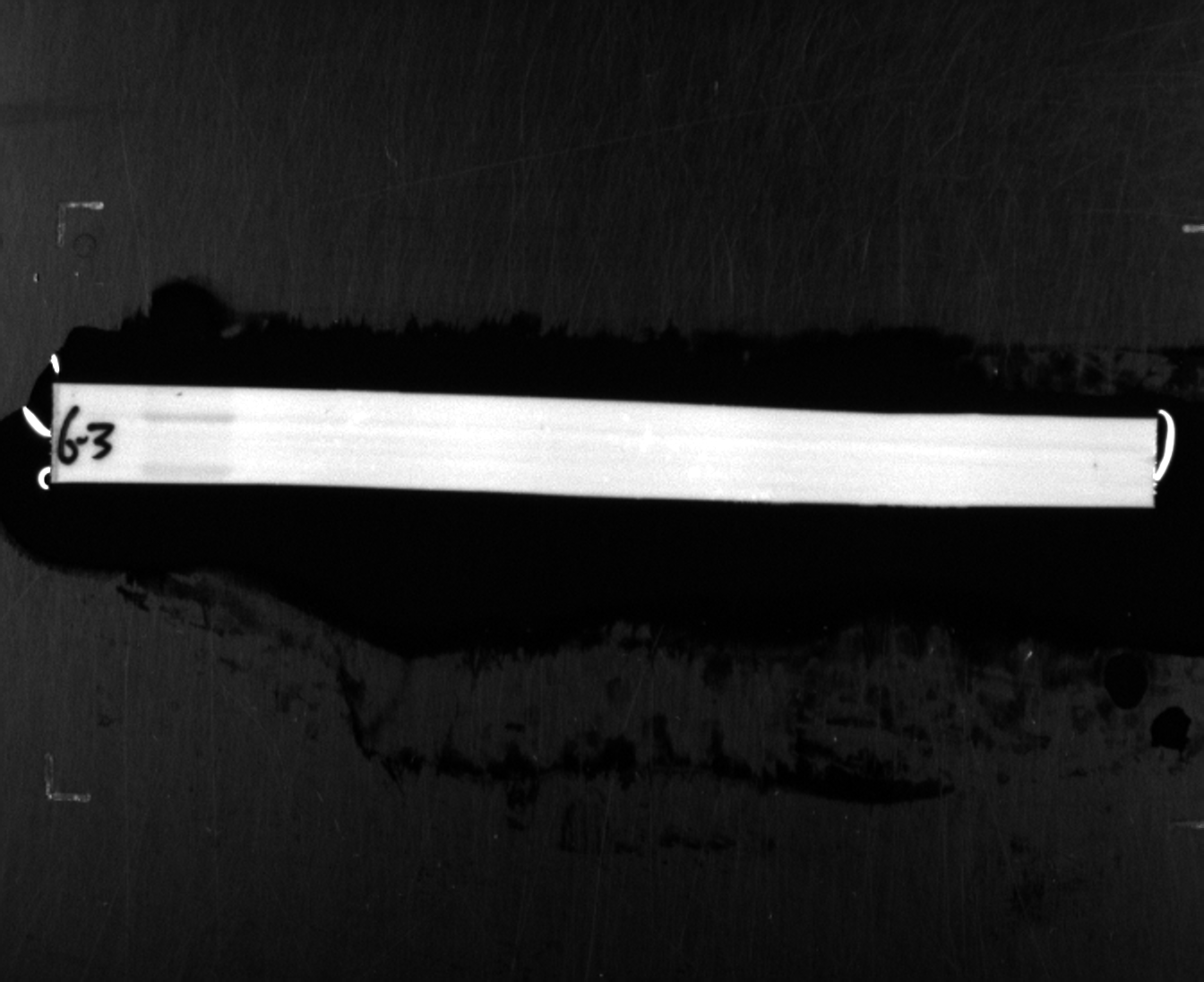

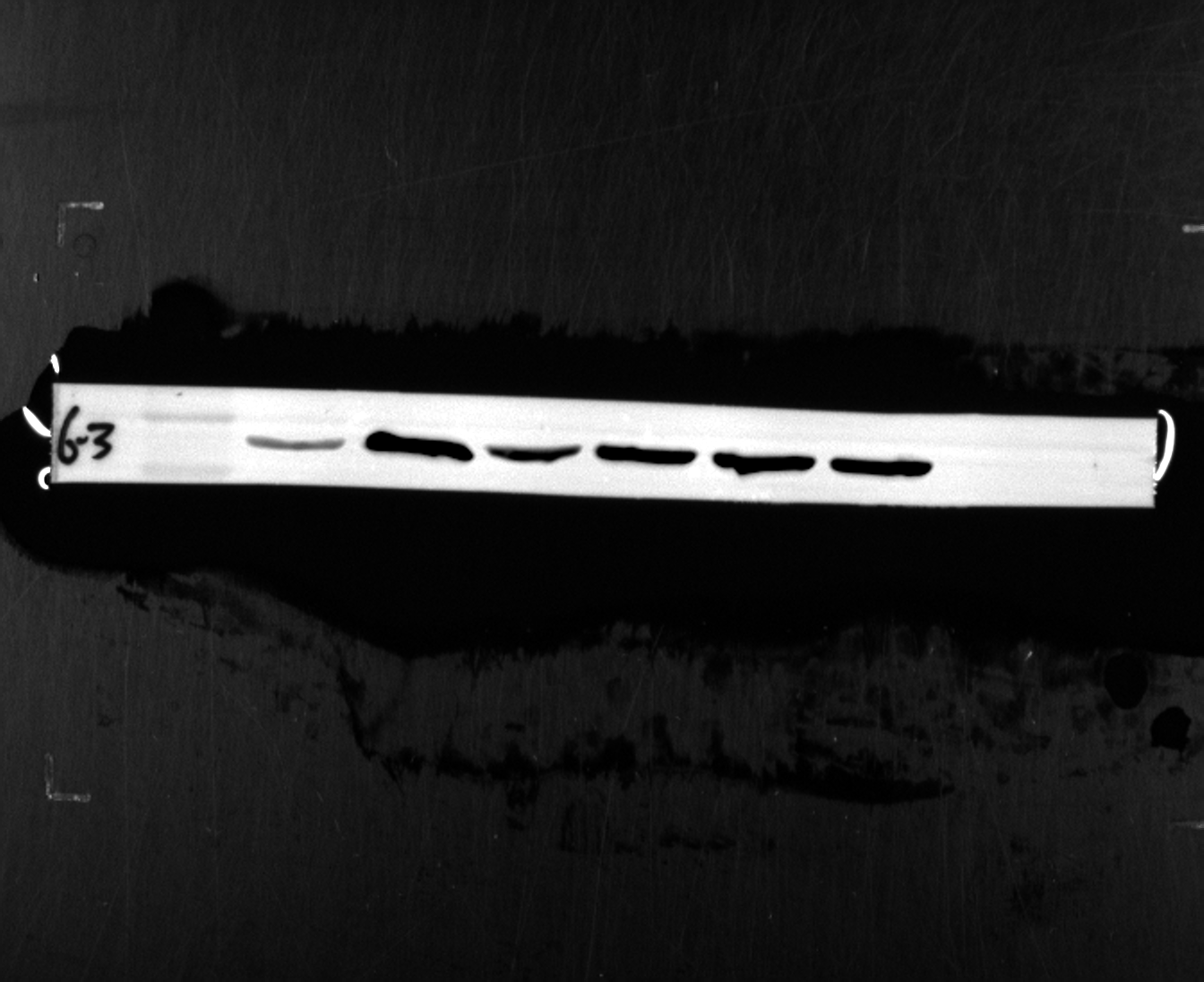


Figure 5D GAPDH 1


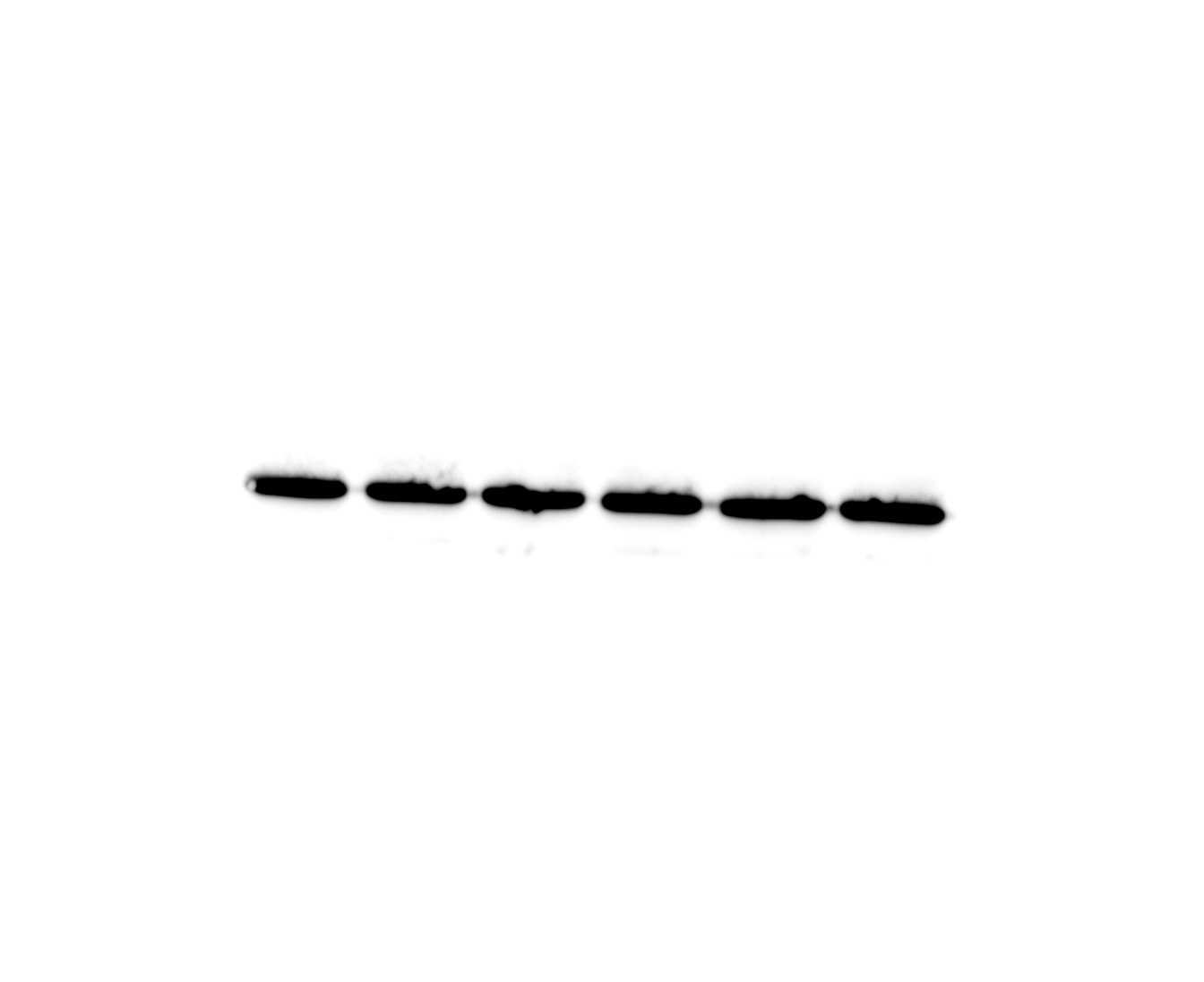

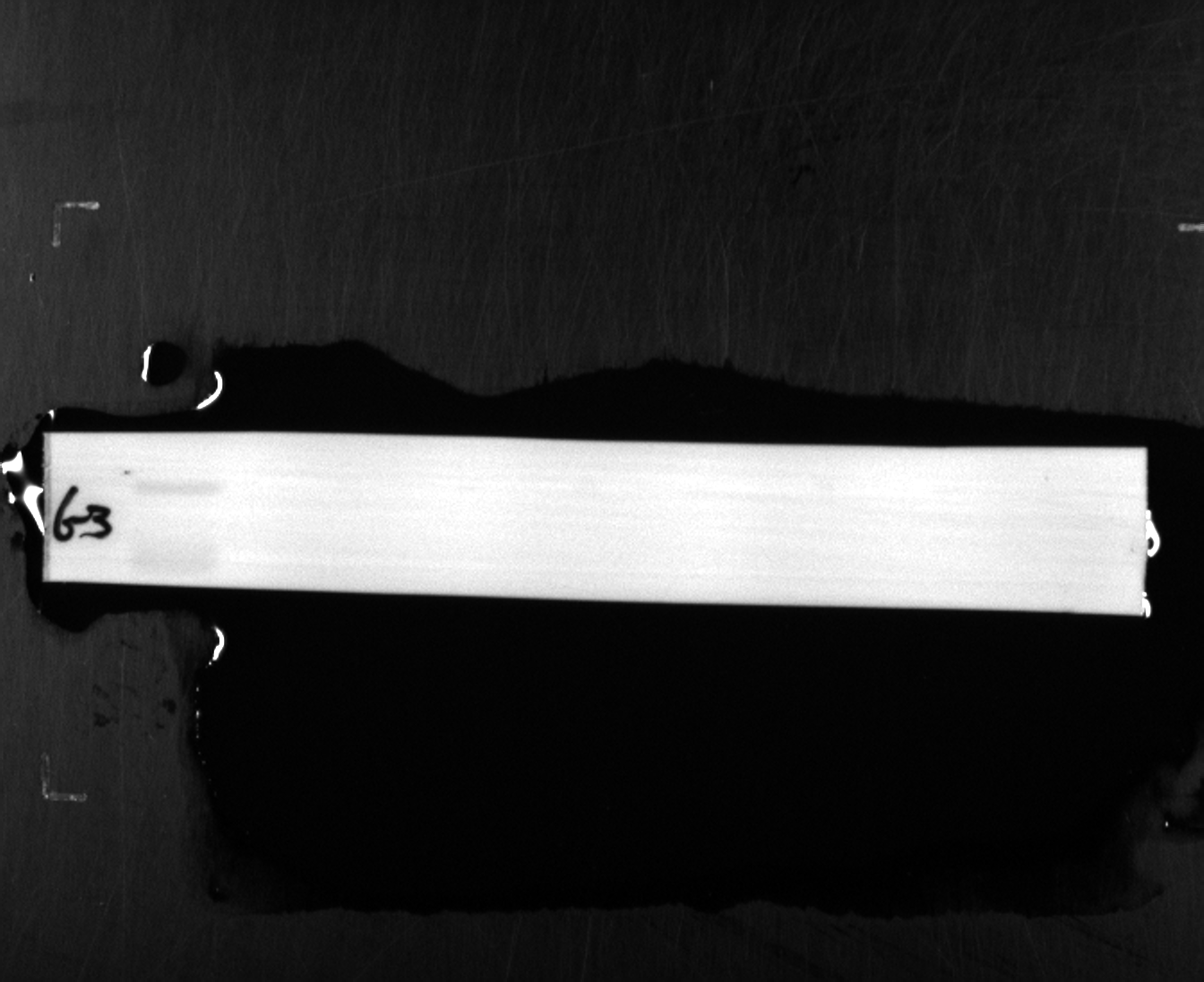

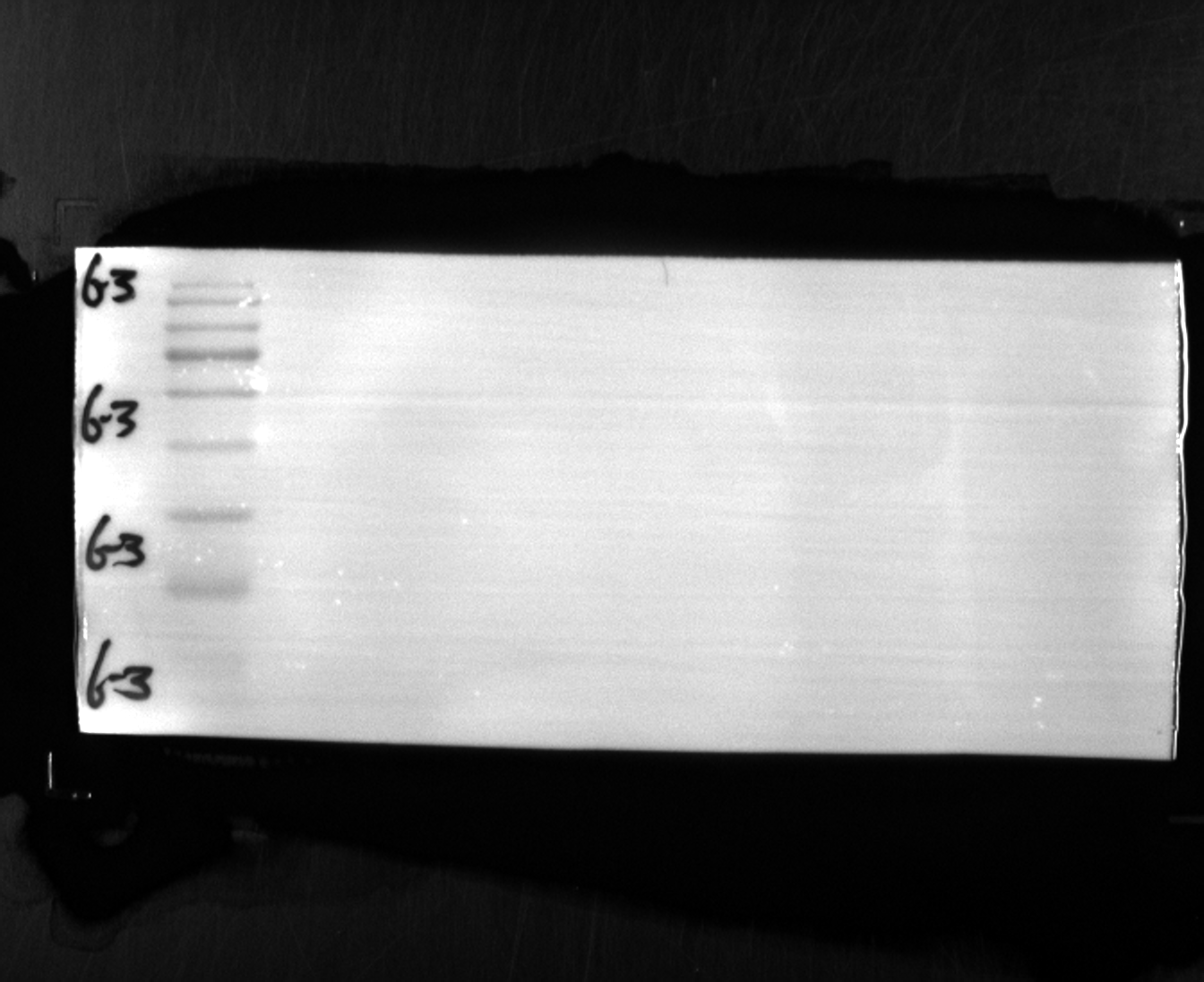

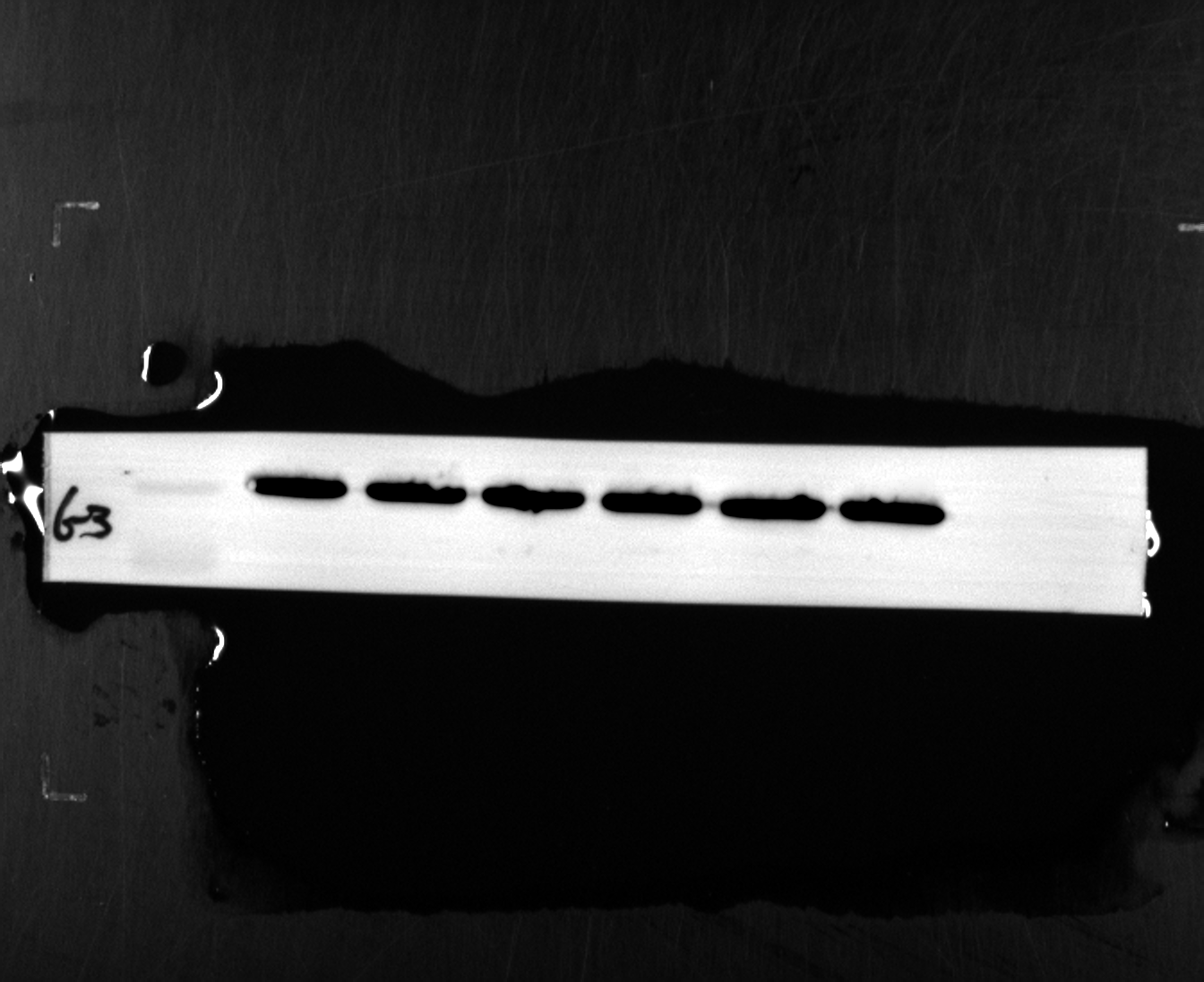


Figure 5D NLRP3


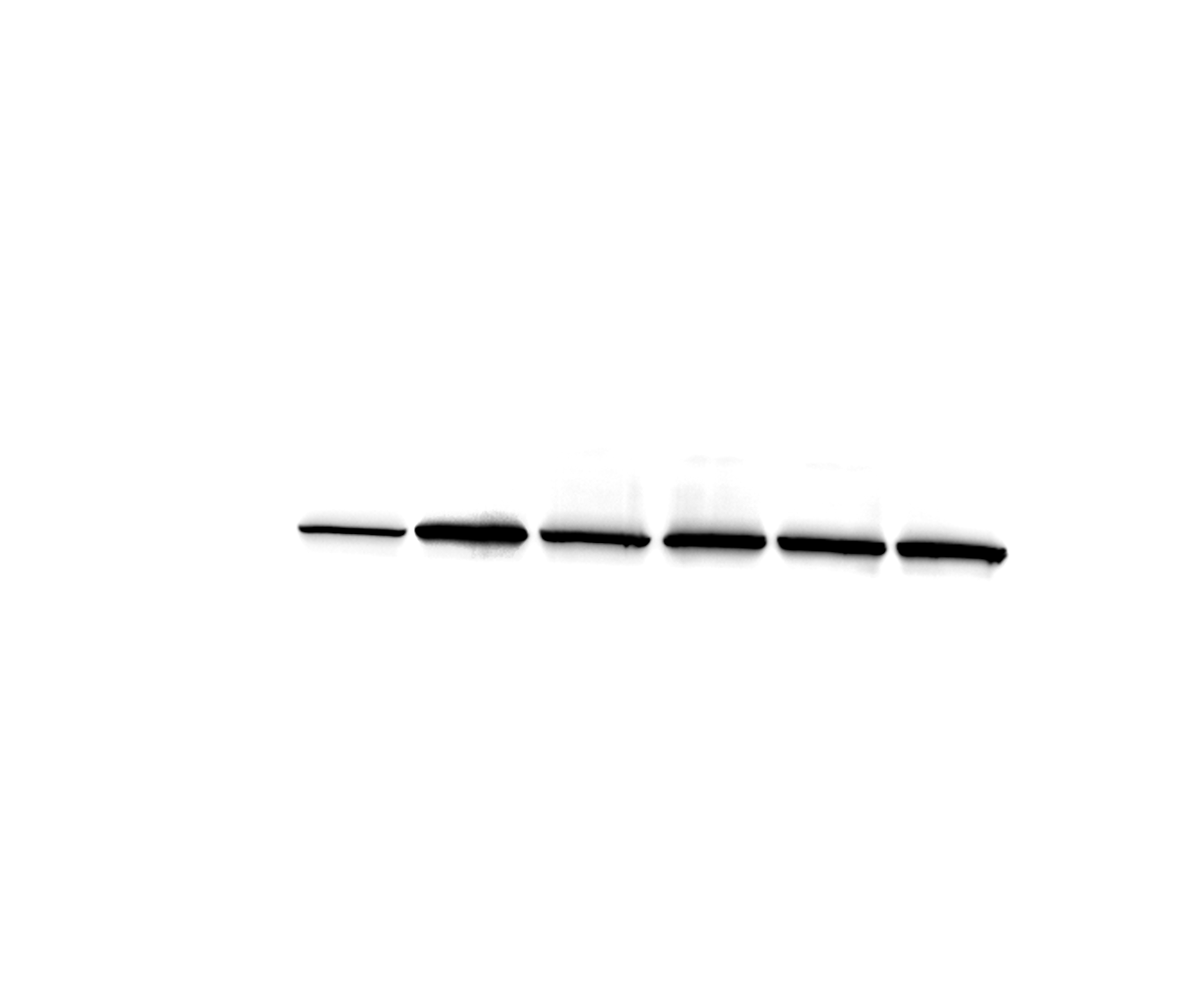

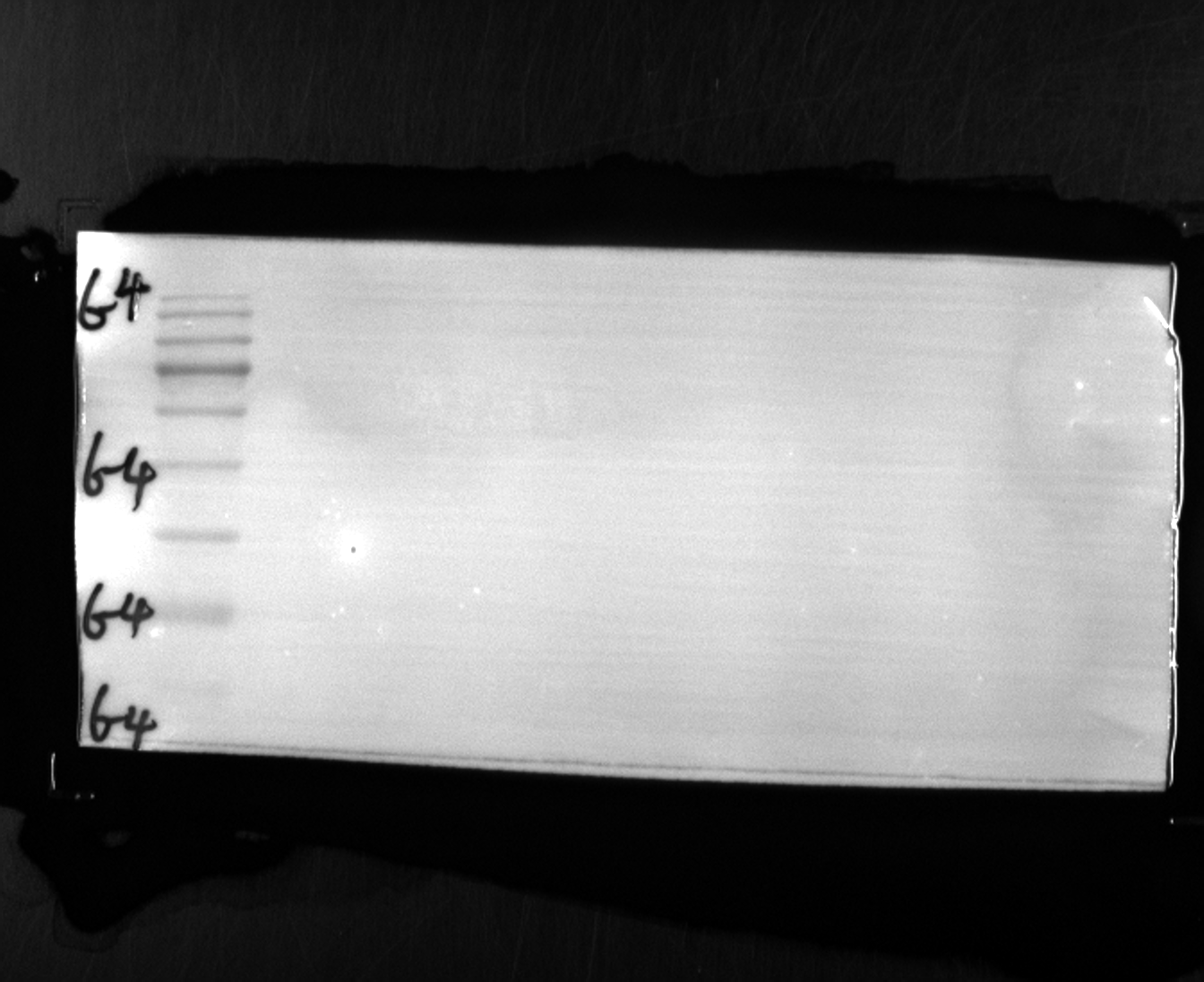

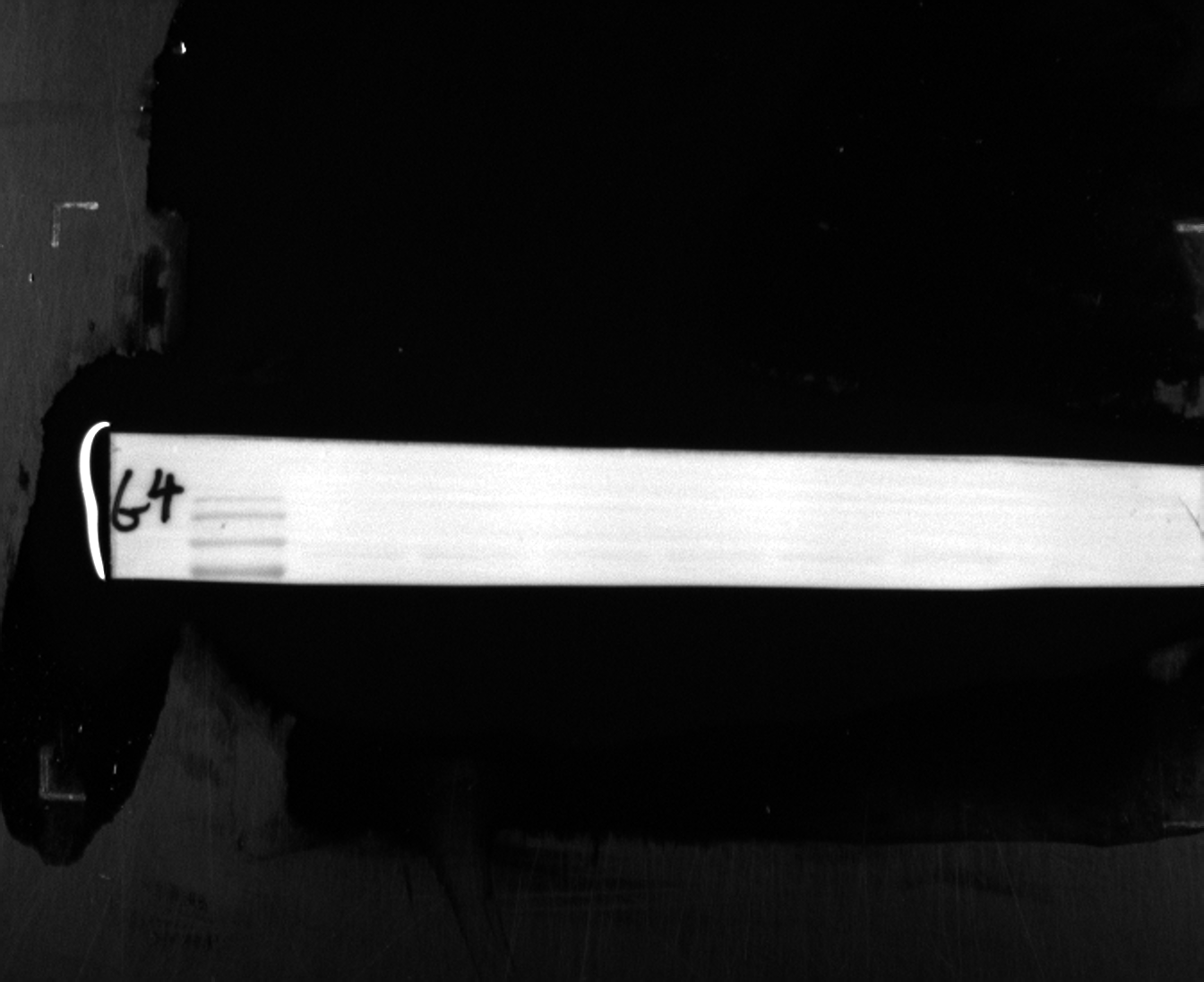

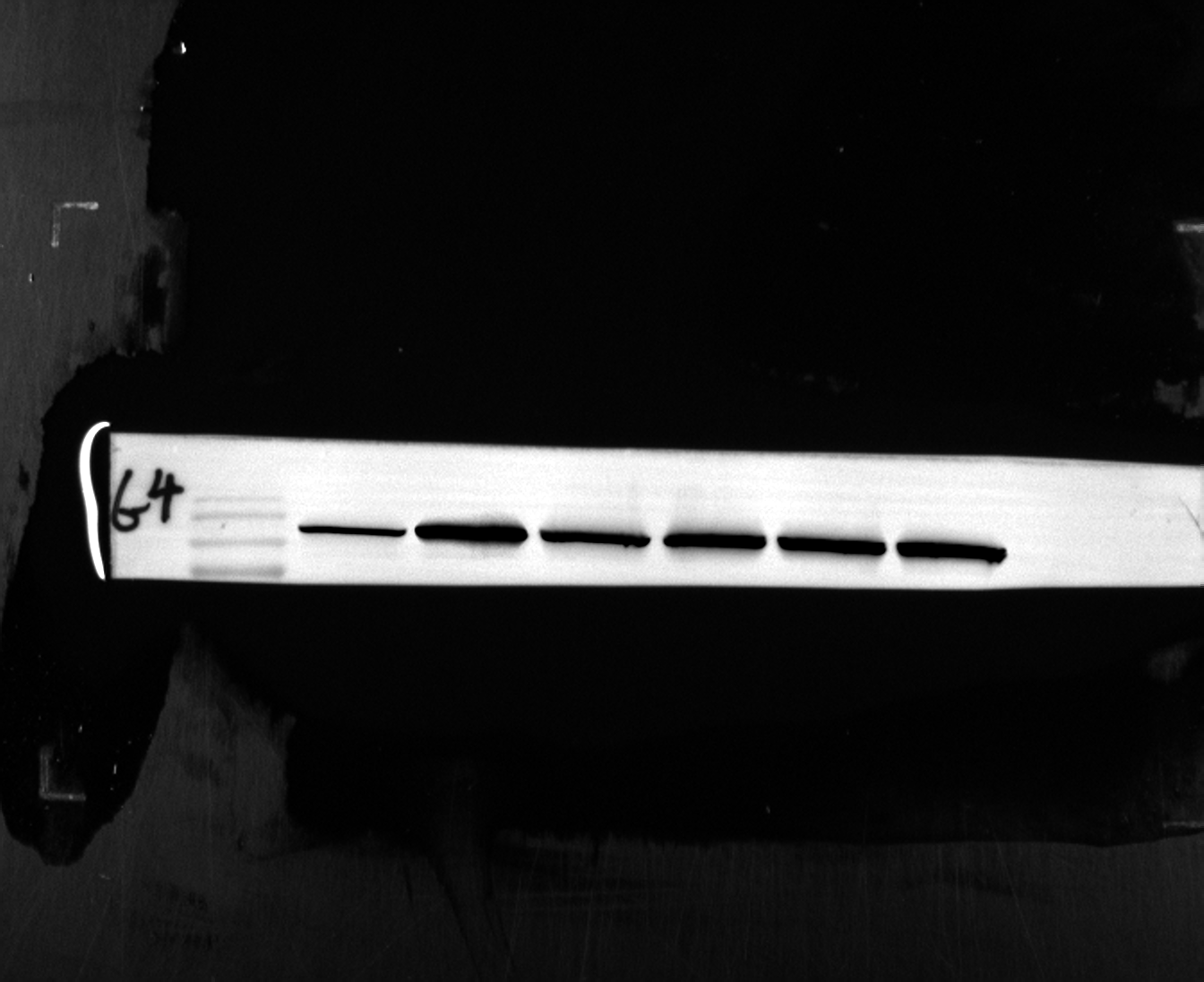


Figure 5D Capase-1


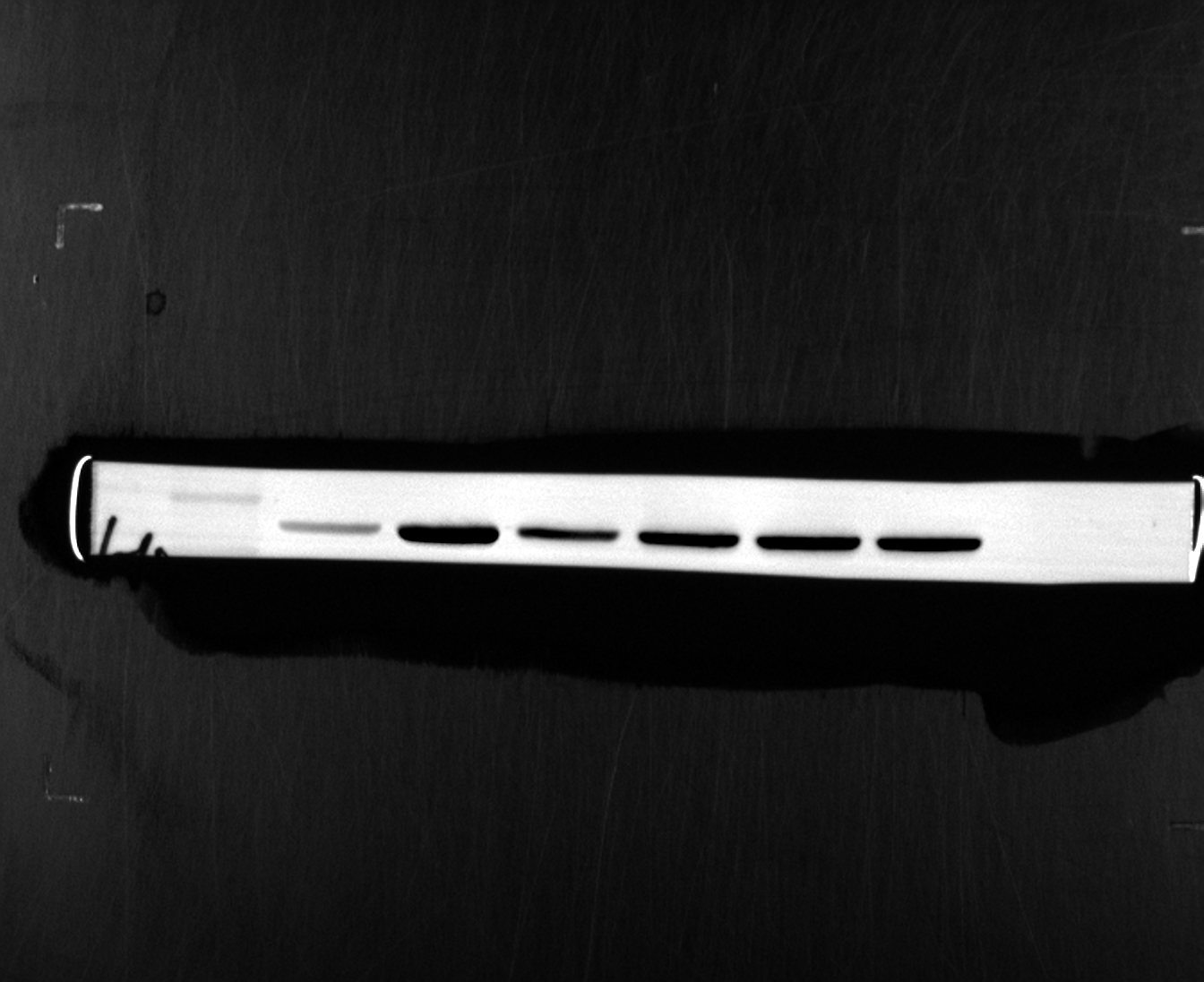

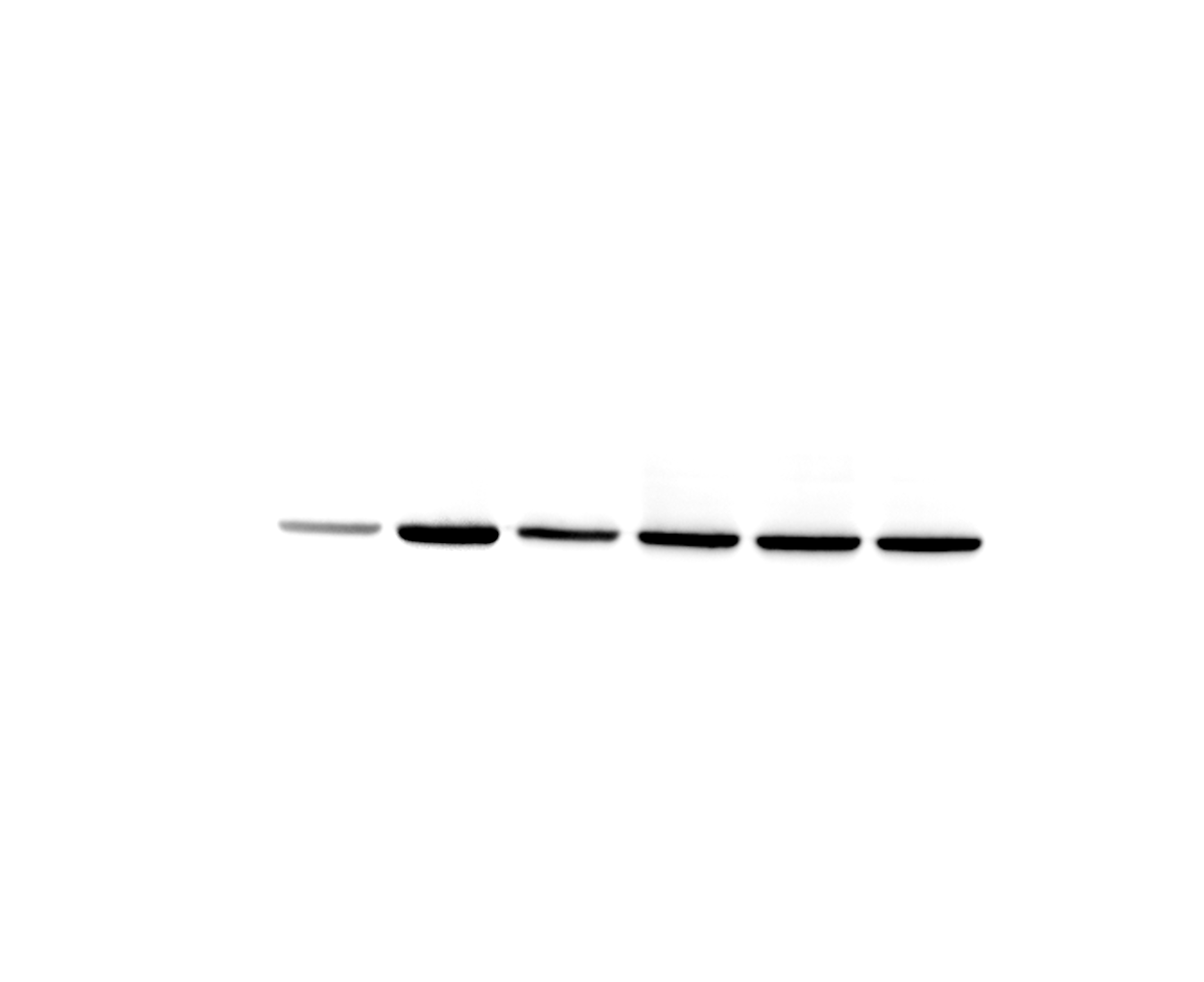

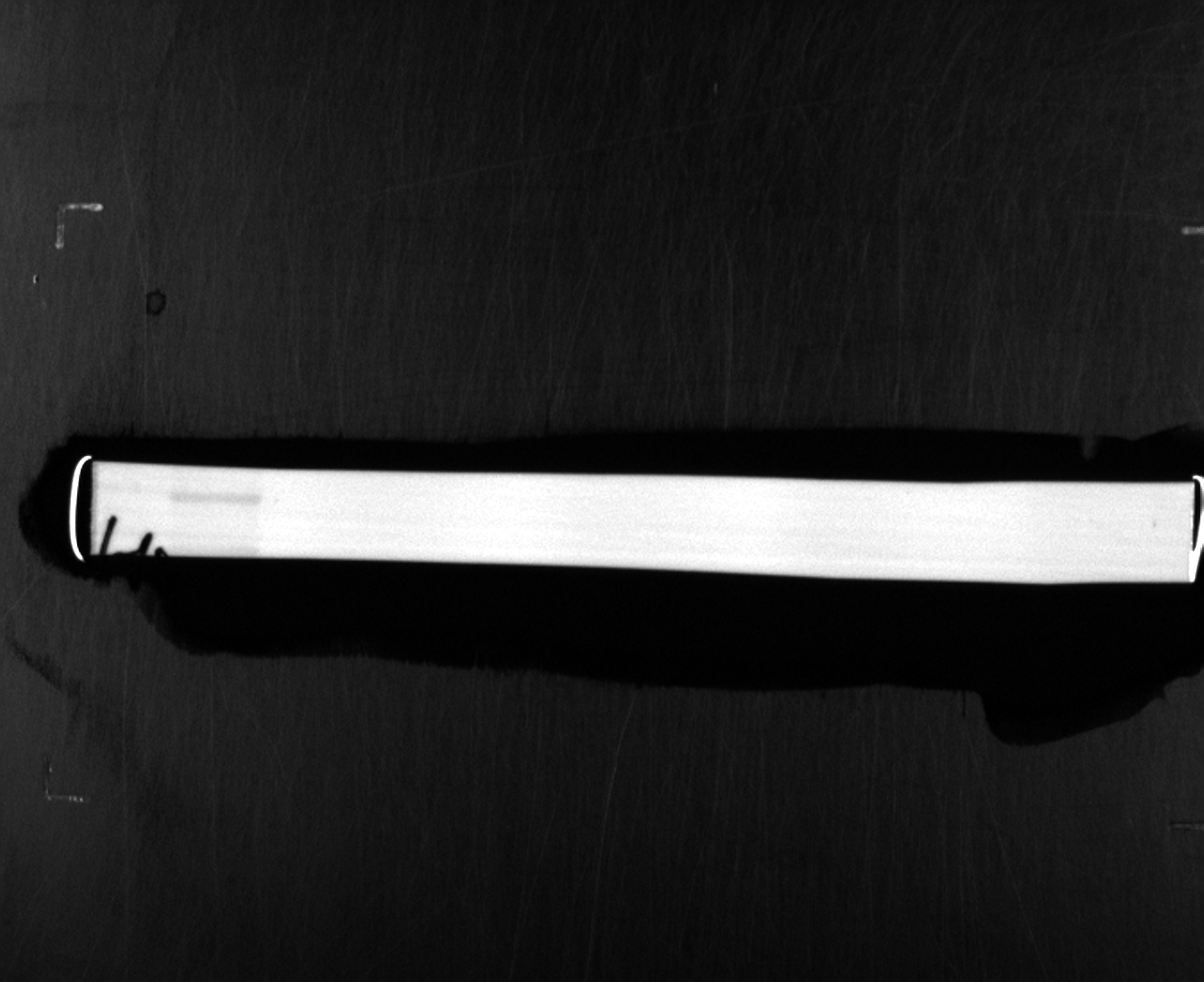

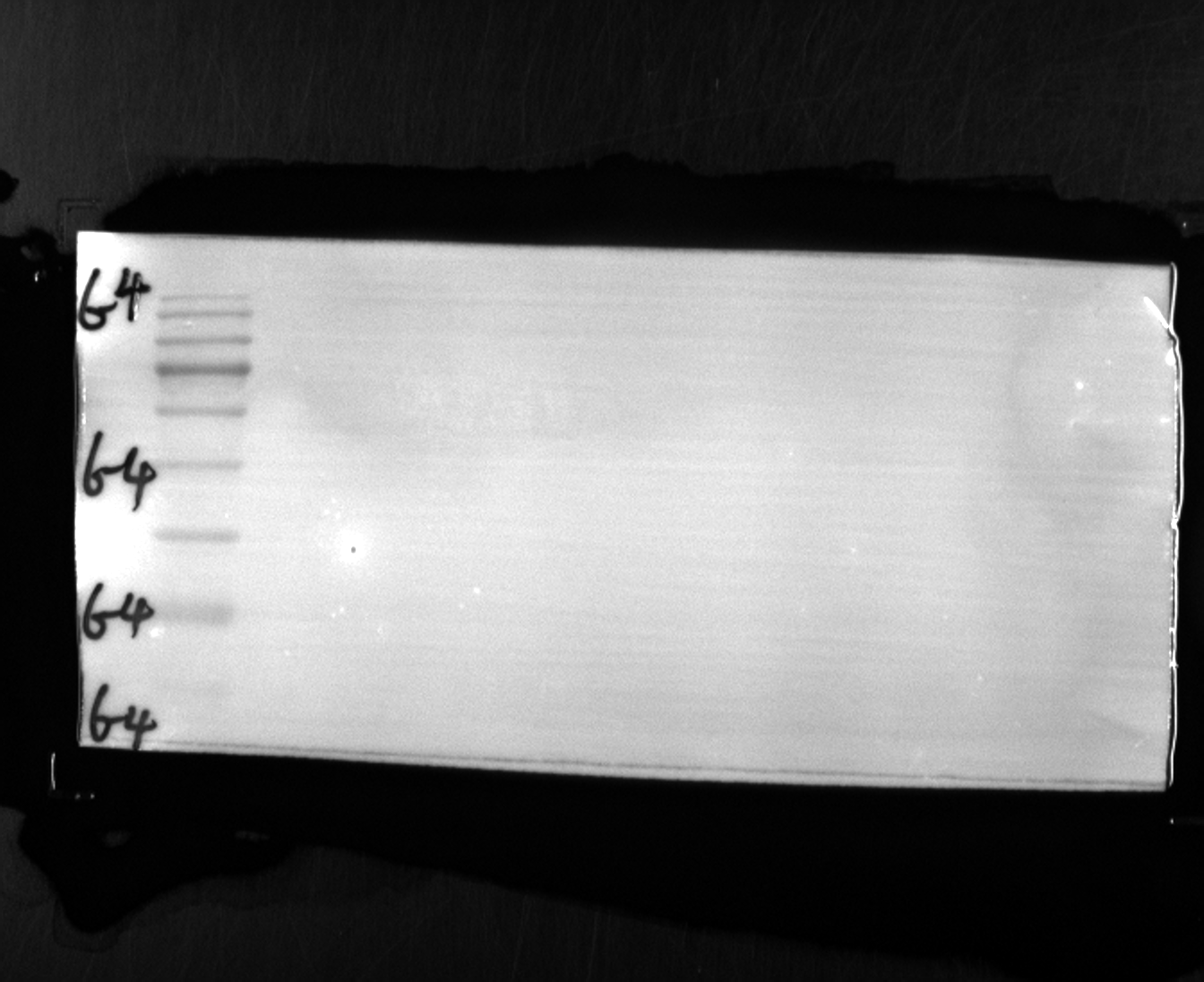


Figure 5D GAPDH 2


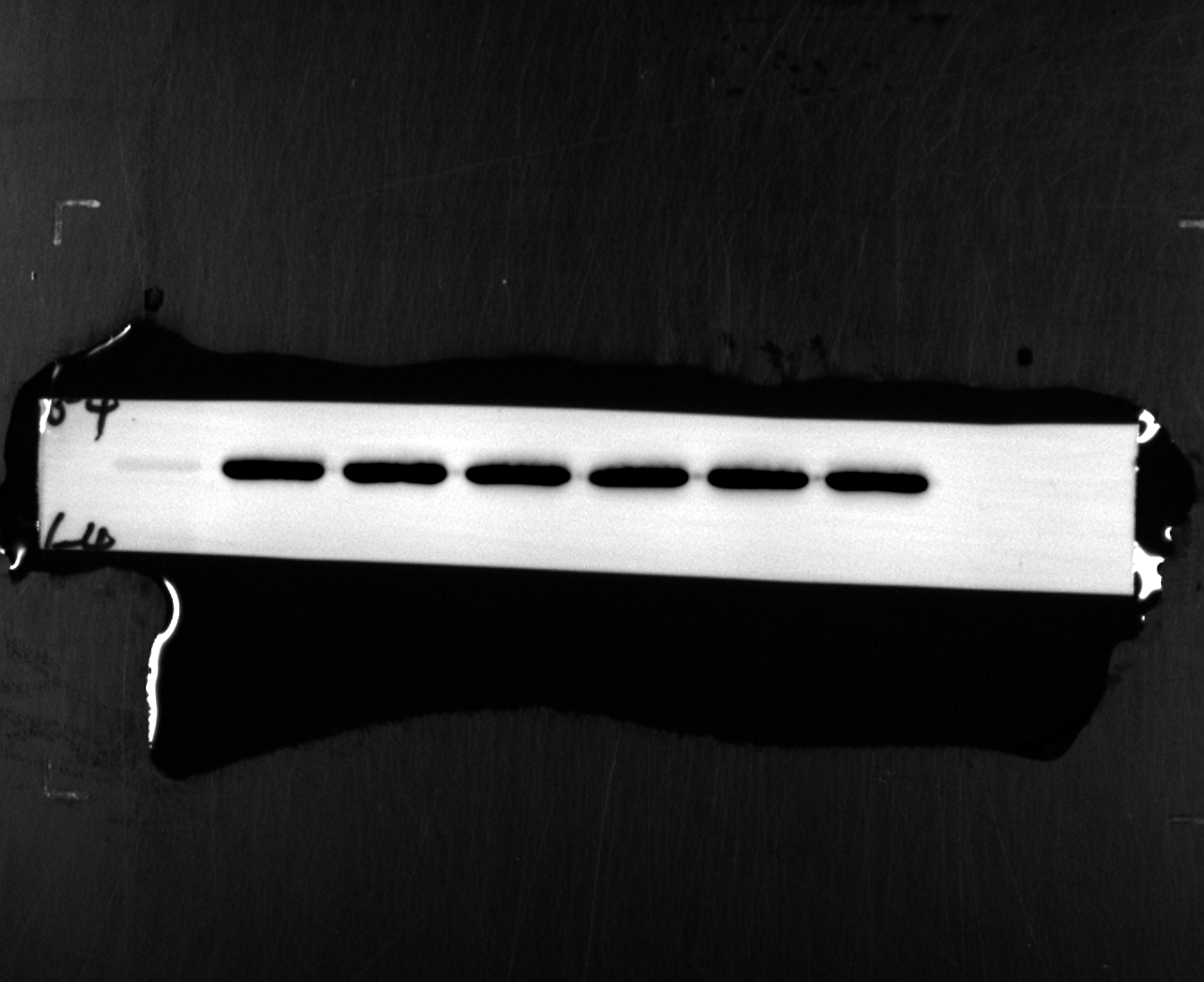

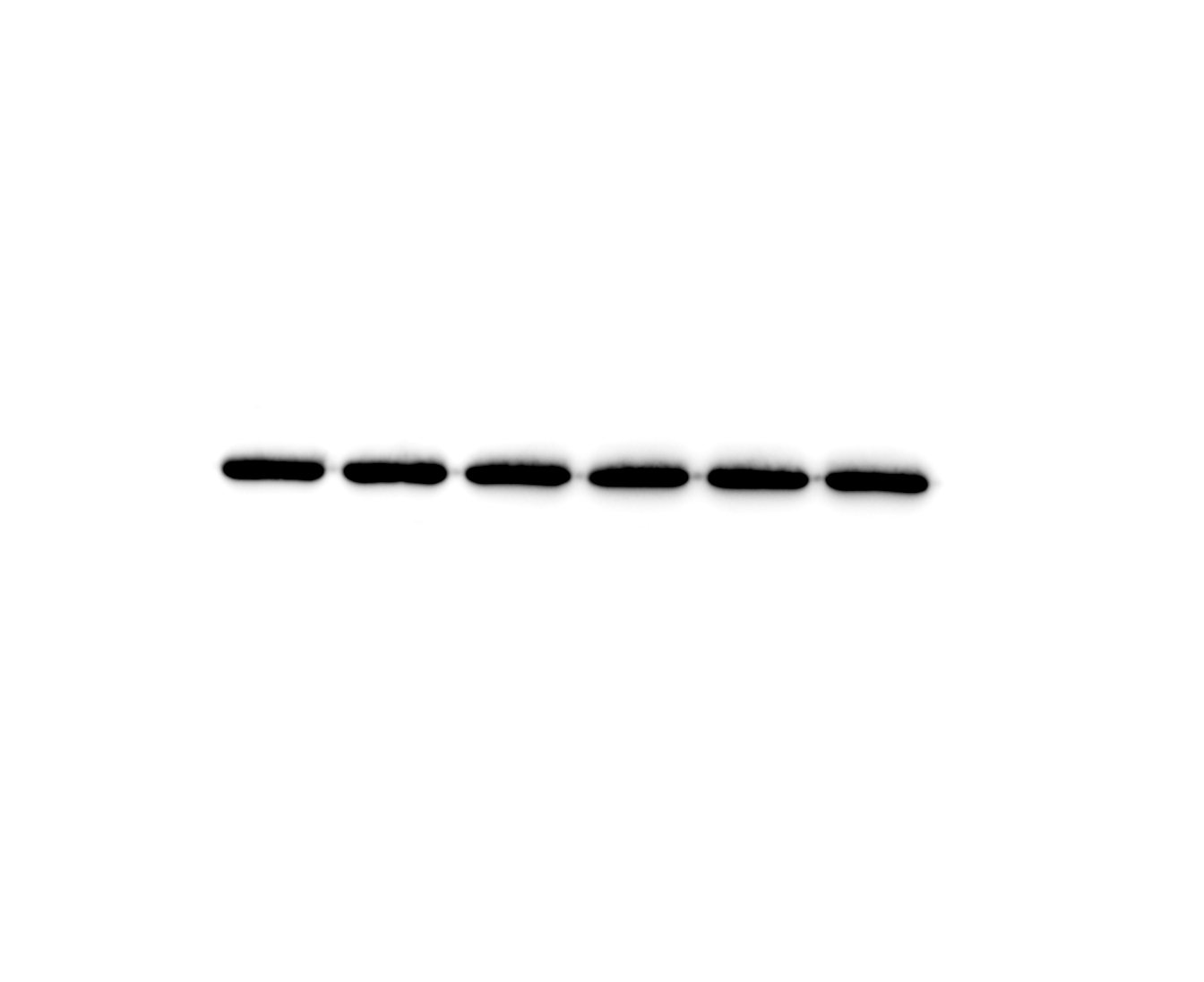

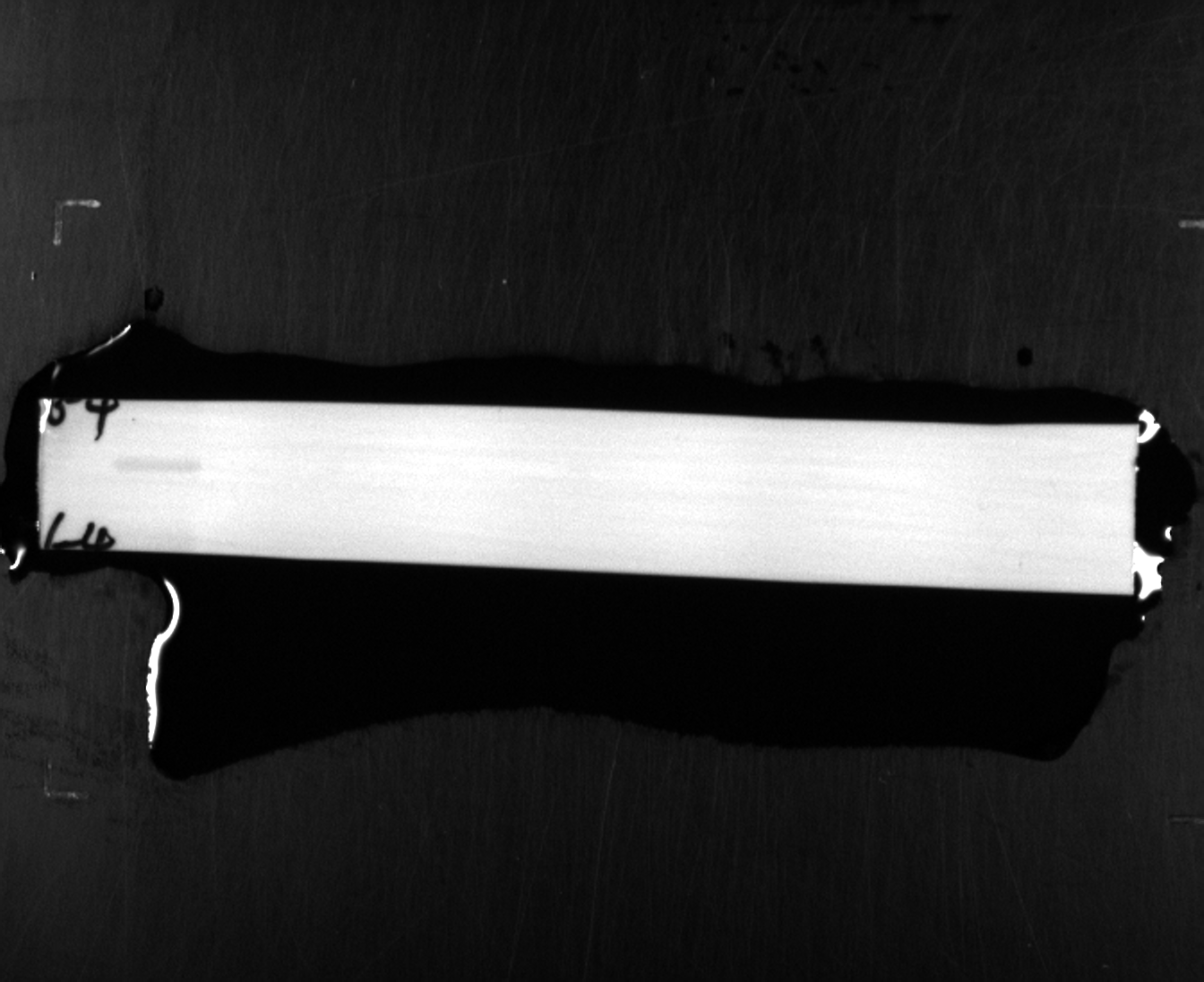

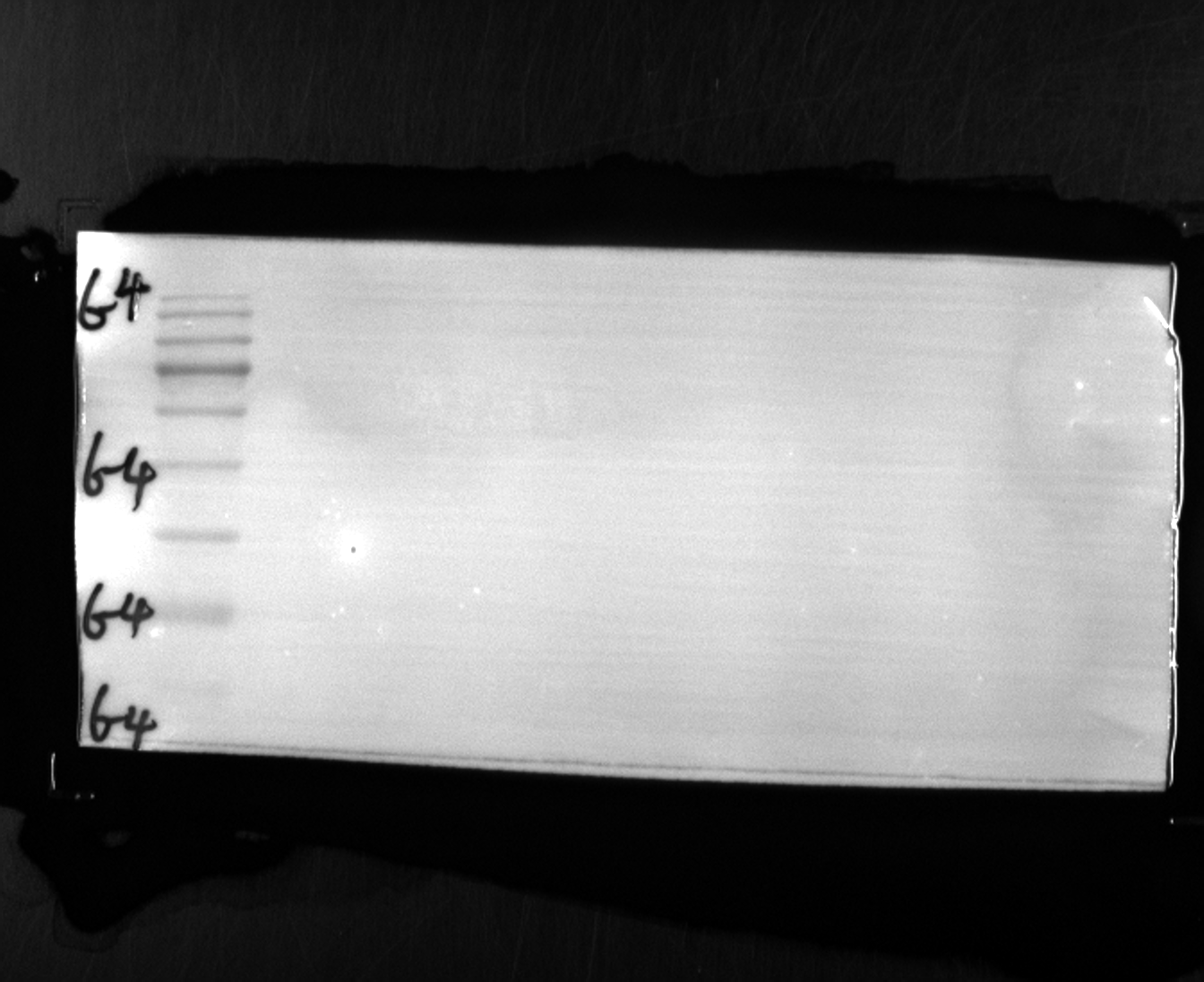

Supplement: Supplementary file 1 — Supplementary Material 1 [file 12886_2024_3396_MOESM1_ESM.docx]
